# How to Handle Speciose Clades? Mass Taxon-Sampling as a Strategy towards Illuminating the Natural History of *Campanula* (Campanuloideae)

**DOI:** 10.1371/journal.pone.0050076

**Published:** 2012-11-28

**Authors:** Guilhem Mansion, Gerald Parolly, Andrew A. Crowl, Evgeny Mavrodiev, Nico Cellinese, Marine Oganesian, Katharina Fraunhofer, Georgia Kamari, Dimitrios Phitos, Rosemarie Haberle, Galip Akaydin, Nursel Ikinci, Thomas Raus, Thomas Borsch

**Affiliations:** 1 Botanischer Garten und Botanisches Museum, Freie Universität Berlin, Berlin, Germany; 2 Florida Museum of Natural History, University of Florida, Gainesville, Florida, United States of America; 3 Institute of Botany, National Academy of Sciences, Erevan, Armenia; 4 Department of Biology, University of Patras, Patras, Greece; 5 Biology Department, Pacific Lutheran University, Tacoma, Washington, United States of America; 6 Department of Biology Education, Hacettepe University, Ankara, Turkey; 7 Department of Biology, Abant Izzet Baysal University, Bolu, Turkey; 8 Department of Biology, University of Florida, Gainesville, Florida, United States of America; University of Oxford, United Kingdom

## Abstract

**Background:**

Speciose clades usually harbor species with a broad spectrum of adaptive strategies and complex distribution patterns, and thus constitute ideal systems to disentangle biotic and abiotic causes underlying species diversification. The delimitation of such study systems to test evolutionary hypotheses is difficult because they often rely on artificial genus concepts as starting points. One of the most prominent examples is the bellflower genus *Campanula* with some 420 species, but up to 600 species when including all lineages to which *Campanula* is paraphyletic. We generated a large alignment of *petD* group II intron sequences to include more than 70% of described species as a reference. By comparison with partial data sets we could then assess the impact of selective taxon sampling strategies on phylogenetic reconstruction and subsequent evolutionary conclusions.

**Methodology/Principal Findings:**

Phylogenetic analyses based on maximum parsimony (PAUP, PRAP), Bayesian inference (MrBayes), and maximum likelihood (RAxML) were first carried out on the large reference data set (D680). Parameters including tree topology, branch support, and age estimates, were then compared to those obtained from smaller data sets resulting from “classification-guided” (D088) and “phylogeny-guided sampling” (D101). Analyses of D088 failed to fully recover the phylogenetic diversity in *Campanula*, whereas D101 inferred significantly different branch support and age estimates.

**Conclusions/Significance:**

A short genomic region with high phylogenetic utility allowed us to easily generate a comprehensive phylogenetic framework for the speciose *Campanula* clade. Our approach recovered 17 well-supported and circumscribed sub-lineages. Knowing these will be instrumental for developing more specific evolutionary hypotheses and guide future research, we highlight the predictive value of a mass taxon-sampling strategy as a first essential step towards illuminating the detailed evolutionary history of diverse clades.

## Introduction

A significant proportion of angiosperm diversity occurs in speciose clades with large numbers of species usually classified as big genera. Aiming at a better understanding of the genesis of biodiversity, such lineages offer unique opportunities to generate and test evolutionary or ecological hypotheses that are fundamental to explain species origin and diversification. Over time, the delimitation and size of such groups, however, fluctuated depending on the “lumping” vs. “splitting” philosophy of the respective taxonomists. Besides the controversial and much debated concept of generic boundary, more than 50 still traditionally circumscribed genera are currently acknowledged to comprise over 500 species and represent some 35% of the known angiosperm diversity [1], [2].

The bellflowers and allies are a well-known example of a plant group with considerable species diversity in the northern hemisphere. They comprise some 420 species in their present delimitation [3], reflected in the current widespread use of the name *Campanula* [hereafter “*Campanula*”]. When derived lineages that are currently recognized as individual genera based on selected morphological characters are included the number of species is 580–600 [hereafter “*Campanula* s.lat.”]. Most members of *Campanula* are annual to perennial herbs, with alternate leaves and pentamerous flowers [4], [5], [6]. The corolla is quite variable in shape, ranging from campanulate to infundibuliform or rotate, with many possible transition forms. The stamens are generally free with characteristic expansions at the base of the filaments forming a protecting lid over the nectariferous disk. The 3- to 5-locular, epigynous ovary exhibits an equal number of stigmatic lobes. Finally, the fruit is a capsule that dehisces by basal to apical pores or valves.

Large genera such as *Campanula* have long disconcerted systematists, who found them either highly fascinating or extremely frustrating because of the difficulty of studying them [7], [8], [9]. So far, comprehensive phylogenetic analyses that include all or most seemingly related species in large putative clades are rare and generally suffer from incomplete taxon sampling, which is known to generate a range of potential analytical problems [10], [11]. To compensate over the problem of missing taxa, most authors generally construct datasets that include only “representative” or “exemplar” taxa. Their selection is usually based on existing classification systems and morphological diversity. However, the predictive value of such pre-cladistic, classification-guided taxon sampling may strongly depend on the extent of homoplasy in morphological characters, and thus may significantly bias phylogenetic analyses.

In *Campanula*, for instance, most morphological characters are highly plastic and poorly help to delineate natural groups [12], [13]. As a result, the taxonomic delimitation of *Campanula* remains unclear, with incomplete and controversial infra-generic classification [14], [15], [16], [17]. Furthermore, none of the DNA-based phylogenetic analyses performed in the last decade [13], [18], [19], [20], [21], [22] provided a comprehensive phylogenetic hypothesis for the bellflowers that could serve as the basis for further attempts in evolutionary analysis and eventually an agreed modern classification system. While generally demonstrating the polyphyly of *Campanula* and many related taxa, a large number of species remained un-sampled. Indeed, none of the existing analyses had gone beyond including 20% of the described number of species, an average reaching rather 10%.

In this study, we aimed at considerably increasing the taxon sampling while keeping the workload and sequencing cost at a minimum level. We therefore applied mass taxon sampling by using a short DNA sequence and generated a large data set for *Campanula* and its allies, with some 310 species of *Campanula* (74%), not including subspecific or varietal entities, and overall 680 accessions (D680; Table S1). In order to test the effect of mass taxon-sampling over a typical sampling guided by pre-cladistic classification, we compared different parameters including tree topology, branch support, and age estimates for nodes between our large dataset (D680) and a much reduced data set (D088) that included the type species of all genera and infrageneric taxa in our study group (Table S2). Additionally, we analyzed a phylogeny-guided dataset of similar size (D101) that included representatives of all subclades recovered from the larger analysis (D680). This allows to test ideas derived from simulation-based results of taxon-addition effects on phylogenetic tree inference achieved in the last years [23], [24], [25], [26], [27] in an empirical context of a large species level data set.

For efficient mass sampling analyses, we used a genomic region with high phylogenetic signal per informative character [28], [29], [30], a requirement fulfilled by chloroplast introns with their mosaic-like structure of helical and stem-loop elements [31]. Unlike coding genes such as *rbcL* or *nr18S* [32], introns have so far never been employed to construct large data sets. Within the *petD* region, we have sequenced a group II intron with well-known secondary structure and molecular evolution [33], and proven phylogenetic utility at the species level [34]. We are aware that mass taxon-sampling using a single (or few) markers may not fully resolve relationships of closely related species but argue that it will be fundamental for developing adequate evolutionary hypotheses that subsequently can be tested.

Using the phylogenetic information provided by the three datasets, the aims of the study are: (1) to test the effects of mass sampling versus lower taxon representation on several phylogenetic estimates including tree shape, branch robustness, and node ages calculation; and (2) to infer an overall phylogenetic hypothesis for *Campanula* and allies, outlining avenues for further research.

## Materials and Methods

### Study Group, Sampling Strategy, Molecular Biology Protocols

#### Study group

Based on previous phylogenetic studies [13], [18], [19], [20], [21], the following subfamilies/tribes/genera [3] have been chosen as outgroups: Lobelioideae-Lobelieae (*Grammatotheca*, *Lobelia*, *Solenopsis*, *Hippobroma*, *Isostoma*); Lobelioideae-Lysipomieae (*Siphocampylus*); Lobelioideae-Delisseeae (*Brighamia*); and Cyphioideae (*Cyphia*). For the ingroup, in addition to the Campanuleae, accessions from all other tribes of the Campanuloideae have been sampled: the Cyanantheae (*Cyananthus*, *Platycodon*, *Codonopsis*, *Cyclocodon*, *Ostrowskia*, and *Canarina*), Wahlenbergieae (*Wahlenbergia*, *Nesocodon*, *Prismatocarpus*, and *Roella*), Edraiantheae (*Edraianthus*, *Feeria*, *Michauxia*, *Trachelium*), Jasioneae (*Jasione*), Musschieae (*Musschia*), Campanuleae (*Adenophora*, *Azorina*, *Favratia*, and *Hanabusaya*), Theodorovieae (*Sachokiella*, *Theodorovia*), Peracarpeae (*Githopsis*, *Heterocodon*, *Legousia*, and *Triodanis*), and Phyteumeae (*Asyneuma*, *Petromarula*, *Phyteuma*, and *Physoplexis*). Most samples were determined or confirmed by specialists belonging to our group of authors (e.g. TR for Greek campanulas, GP, GA, and NI for Turkish ones, or MO for Caucasian ones). Information on voucher specimens, and Genbank numbers of *petD* accession newly generated for this study, are given in Table S1.

#### Sampling strategy

**Figure 1:**
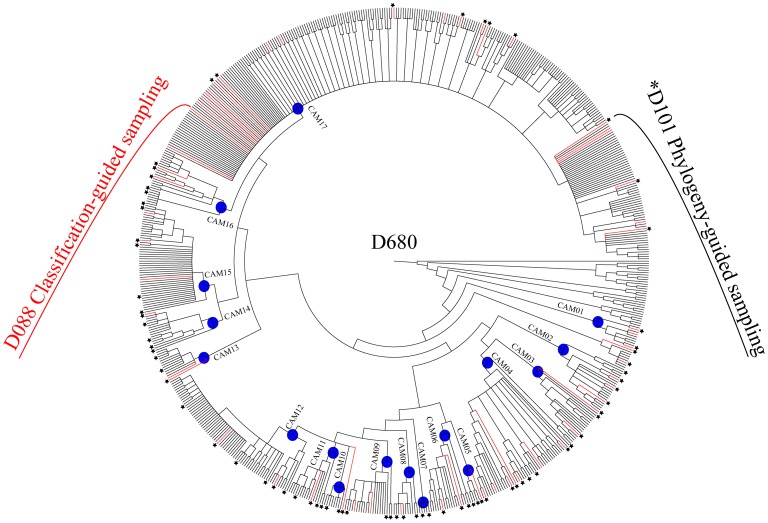
Overview of the sampling strategy. The circular cladogram represents the Maximum Parsimony strict consensus tree inferred from the mass sampling (MS, D680). Dotted lines (red) indicate accessions sampled for the classification-guided sampling (CS, D088). Asterisks refer to accessions sampled for the phylogeny-guided sampling (PS, D101). Blue dots indicate crown groups for the respective "Cam" clades containing at least one accession of *Campanula* (Cam01 to Cam17; see text). LOBE = Lobelioideae; CYPHI: Cyphioideae; CA-CYA: Campanuloideae-Cyanantheae; CA-WAH: Campanuloideae-Wahlenbergieae.

To test for the effect of different sampling schemes on the inferred phylogenetic hypothesis and on divergence time estimates, we performed all molecular analyses on three different datasets. We first generated a large data set with 680 accessions (D680), based on “mass sampling” (MS) of taxa and including some 74% of the diversity ascribed to *Campanula* (310 out of 420 species; [3]). We then pruned the large matrix, to generate data sets resembling a “classification-guided sampling” (CS) and a “phylogeny-guided sampling” (PS). In the first case (CS), we selected 42 type species for the respective subgenera/sections described in *Campanula* (Table S2), along with a single representative of the paraphyletic genera embedded in *Campanula* s.lat. (Table S1). The final CS-based dataset contained 88 accessions (D088) and could be considered as obtained by an “a priori”, classification-informed sampling strategy. In the second case (PS), we selected only a limited number of taxa as representatives of those clades that were inferred from analyzing D680. In our case, the 101-taxon matrix (D101) effectively was created “a posteriori” but can be used to test for the effect of low taxon density while keeping the phylogenetic diversity optimally represented. An overview of all sampling strategies is given in Fig. 1.

#### Molecular biology protocols

Total DNA extraction, PCR amplification, and sequencing of the *petD* region of cpDNA followed protocols described in Borsch et al. [18]. Sequences were aligned using Muscle [35], with additional manual corrections in PhyDe [36], on the premise of hypothesized microstructural events (motif-based alignment). Indels were coded as binary characters with SeqState [37] and added at the end of the matrix. Subsequent phylogenetic analyses were performed by excluding a microsatellite region of 15 characters located in position 736–750 of the D680 final alignment (12 characters in D101 and D088).

### Phylogenetic Inference, Molecular Dating

#### Phylogenetic inference

Aligned matrices were analyzed using the respective maximum parsimony (MP), Bayesian inference (BI), and maximum likelihood (ML) approaches (Table 1). Phylogenetic trees were further edited with FigTree [38]. The MP analyses, using a Fitch criterion, were performed using version 4.0b10 of PAUP [39]. Heuristic searches were conducted with a ratchet batchfile, including 200 iterations, each of them with 25% of the positions randomly weighted (weight = 2), and 100 random additions, generated with PRAP [40]. Branch support was calculated with the bootstrap (BS) method, using 10,000 replicates, TBR branch swapping, 10 random-additions, multrees option OFF, and resampling all characters. In the same way, jackknife (JK) values were computed with 36.788% of characters deleted in each replicate.

The BI analyses were conducted with MRBAYES [41], using six simultaneous runs of Metropolis-coupled Markov Chain Monte Carlo (MC3), under a GTR+G+I model of sequence substitution selected using the Akaike Information Criterion in MRMODELTEST [42], and a binary model (Lset coding = variable) applied to the coded gaps. Each chain was run in parallel for 10 million generations, saving one tree each 10,000^th^ generation, keeping a default temperature parameter value of 0.2. The MC3 runs were repeated twice, and the first 10 per cent of the saved trees were discarded as burn-in after checking for (i) stationarity on the log-likelihood curves; (ii) similarity of the respective majority-rule topologies and final likelihood scores; (iii) the values of standard deviation of split frequencies (<0.001); and (iv) the value of the potential scale reduction factor (close to 1). The remaining trees were used to produce a majority-rule consensus tree and to calculate the posterior probability (pp) values.

**Table 1:** Characteristics of the respective phylogenetic analyses for the three datasets (D088, D101, and D680).

	D088	D101	D680
**Characters**			
Total aligned length	1239	1264	1486
Number of coded indels	138	151	243
**Parsimony Analyses**			
Parsimony-informative characters (%)	368 (29,5)	405 (32,0)	622 (41,8)
N trees	708	1799	18852
Length	1381	1587	2503
Consistency Index	0,644	0,601	0,499
Retention Index	0,843	0,827	0,928
Rescaled Index	0,543	0,497	0,463
Number and percent of supported nodes (BS>50)	56 (65%)	75 (76%)	265 (39%)
Number and percent of supported nodes (JK>50)	57 (66%)	77 (78%)	296 (44%)
**Bayesian Analyses**			
Model of sequence evolution (Akaike)	GTR+I+G	GTR+I+G	GTR+I+G
Number of saved trees	10000	10000	10000
Number and percent of supported nodes (pp>50)	61 (71%)	81 (82%)	327 (48%)
**Maximum Likelihood Analyses**			
Model of sequence evolution (default)	GTR+G	GTR+G	GTR+G
Likelihood score of best tree	−8646,156467	−9859,839022	−15871,72173
Number and percent of supported nodes (BS>50)	63 (73%)	80 (81%)	290 (43%)

BS = bootstrap, JK = jackknife, pp = posterior probability.

Finally, the ML analyses were performed with RAxML [43], using the default model of sequence evolution, with the following parameters: (1) 10 to 100 runs using a fast hill-climbing algorithm for the optimal ML tree calculation (option d with GTRGAMMA) and (2) 1000 BS replicates using a fast hill-climbing algorithm for BS calculation (option a with GTRCAT).

#### Molecular dating

A likelihood-ratio (LR) test, performed by comparing the likelihood scores of the respective trees with and without a clock [44], revealed the absence of rate constancy in the respective datasets (D680: LR = 945, df = 678, P<0.001; D101: LR = 426, df = 99, P<0.001; D088: LR = 402, df = 86, P<0.001). Consequently, divergence times were estimated by using the penalized likelihood (PL) method implemented in r8s [45], [46].

Optimal smoothing values were calculated for each dataset by a cross-validation procedure, and 1000 phylograms were generated from bootstrap resampling in RAXML to calculate node ages for the BI majority-rule cladogram. Nodal ages obtained from the 1000 phylograms were summarized with the “profile” command, and the resulting standard deviations were used to derive 95% confidence intervals for the point estimates obtained using the BI majority-rule cladogram.

Two nodes constraints were used to generate a phylogram: (1) a maximum age of 80 million years was set for the root, based on previous studies that inferred the approximate age of the split between Rousseaceae and the lineage leading to the Campanulaceae to be 80 mya [47], [48]; and (2), a fossil constraint was placed at the node of the most recent common ancestor of *Campanula pyramidalis* and *Campanula carpatica*, following Cellinese et al. [19]. The Campanulaceae have a very poor fossil record. However, one reliable account exists for *Campanula* in the form of fossilized seeds of *C. palaeopyramidalis* dating from the Miocene (16.5–17.5 mya) [49]. Values of the respective dated nodes and confidence intervals were visualized with the R package Phyloch [50].

Finally, in order to quantify the pairwise differences between the respective age and branch support values obtained for the different datasets, at both the crown and stem nodes for 22 selected clades (44 nodes; Table 2), we performed a Wilcoxon signed rank test, using the Stats package in R [50].

**Table 2:** Branch support and age estimates for selected outgroups, sister clades, and main *Campanula* clades (CAM01 to CAM17) discussed in this study.

		D088						D101						D680					
Clades	Node	Age Estimate		Branch Support				Age Estimate		Branch Support				Age Estimate		Branch Support			
		Mean	95% CI	MP_BS	MP_JK	BI	ML_BS	Mean	95% CI	MP_BS	MP_JK	BI	ML_BS	Mean	95% CI	MP_BS	MP_JK	BI	ML_BS
LOBE	S	76,20	74,09–78-26	100	100	1	100	75,71	72,60–78,01	100	100	1	100	75,96	38,44–78,42	100	100	1	100
	C	41,64	29,67–49,53					33,73	26,85–37,37					28,95	16,49–40,55				
CYPHI	S	60,61	51,09–66,54	100	100	1	100	58,31	53,77–65,00	100	100	1	100	60,12	32,13–68,32	100	100	1	100
	C	23,67	12,69–40,36					19,63	11,13–25,61					18,98	12,49–31,14				
CA-CYA	S	56,88	19,79–64,68	100	100	1	100	54,20	49,22–62,47	100	100	1	100	56,56	27,18–62,43	100	100	1	100
	C	40,12	28,57–49,72					35,09	28,51–44,41					35,17	19,22–41,59				
CA-WAH	S	42,23	30,85–50,58	100	100	1	100	38,96	35,96–44,52	100	100	1	100	42,52	25,35–49,22	100	100	1	100
	C	26,84	19,23–38,45					25,81	21,99–33,86					30,36	18,55–36,31				
Campanula	S	42,23	30,85–50,58	74	83	0,91	68	38,96	35,96–44,52	72	82	0,87	64	42,52	25,35–49,22	80	91	0,99	83
s.l.	C	40,03	28,19–47,65					36,65	34,29–42,89					39,96	24,81–47,91				
Cam01	S	40,03	28,19–47,65	100	100	1	100	36,65	34,29–42,89	100	100	1	100	39,45	24,81–47,91	100	100	1	100
	C	15,73	9,39–25,22					13,4	8,65–19,98					12,90	8,37–22,68				
Cam02	S	33,26	20,72–44,46	100	100	1	100	29,71	24,97–35,11	99	100	1	100	31,71	16,72–38,53	99	100	1	100
	C	11,52	3,99–16,29					18,58	13,42–25,75					18,36	9,89–23,68				
Cam03	S	33,31	20,72–44,46	100	100	1	100	27,78	24,97–35,11	100	100	1	100	29,90	16,72–38,53	100	100	1	100
	C	11,10	3,76–20,23					9,53	4,29–12,47					10,57	6,15–16,53				
Cam04	S	33,31	20,72–44,46	100	100	1	100	27,78	24,97–35,11	100	100	1	100	29,90	16,72–38,53	100	100	1	100
	C	16,28	7,97–25,72					13,9	10,96–17,93					18,86	9,49–21,97				
Cam05	S	31,95	20,66–43,35	n/a	n/a	n/a	n/a	30,49	27,00–37,19	n/a	59	0,55	55	32,52	20,37–40,35	n/a	62	0,66	64
	C	n/a	n/a					29,97	25,19–35,77					32,10	n/a				
Cam06	S	31,95	20,66–43,35	100	100	1	100	30,49	27,00–37,19	95	98	1	99	32,52	20,37–40,35	96	99	1	98
	C	8,62	1,51–23,39					12,72	5,70–19,95					9,13	5,70–17,48				
Cam07	S	n/a	n/a	n/a	n/a	n/a	n/a	29,38	24,80–34,16	100	100	1	100	30,86	18,58–35,81	100	100	1	100
	C	n/a	n/a					0,66	0,02–2,46					0,22	0,02–1,69				
Cam08	S	25,38	11,90–31,36	n/a	n/a	n/a	n/a	25,41	20,63–29,64	100	100	1	100	26,30	18,35–31,67	99	100	1	100
	C	n/a	n/a					9,32	3,52–13,35					7,55	3,29–14,73				
Cam09	S	22,19	9,55–29,42	100	100	1	100	22,88	19,19–25,57	100	100	1	100	23,11	18,18–28,16	100	100	1	100
	C	4,89	1,27–9,04					12,94	8,73–18,06					13,10	4,60–17,55				
Cam10	S	n/a	n/a	n/a	n/a	n/a	n/a	18,84	16,50–20,35	100	100	1	100	18,54	16,50–21,83	100	100	1	100
	C	n/a	n/a					2,93	0,03–5,07					2,07	0,04–6,49				
Cam11	S	16,5	6,82–19,38	n/a	n/a	n/a	n/a	18,84	16,50–20,35	62	63	0,98	78	18,54	16,50–21,83	58	64	0,99	68
	C	n/a	n/a					17,96	16,50–19,01					17,76	16,50–18,27				
Cam12	S	16,5	6,82–19,38	100	100	1	100	18,84	16,50–20,35	99	100	1	100	18,54	16,50–21,83	99	100	1	100
	C	8,13	2,38–10,04					10,37	6,27–13,09					11,13	5,85–14,91				
Cam13	S	37,55	24,61–46,69	54	59	0,78	84	32,91	28,05–38,47	56	62	0,76	86	35,04	19,21–42,54	52	62	0,81	87
	C	35,11	n/a					29,7	n/a					28,22	13,92–35,88				
Cam14	S	25,59	14,11–35,01	n/a	n/a	n/a	n/a	21,84	16,63–27,58	94	95	1	97	21,71	8,94–26,74	90	95	1	95
	C	n/a	n/a					19,88	12,01–25,20					19,85	9,76–26,18				
Cam15	S	25,59	14,11–35,01	100	100	1	100	21,84	16,63–27,58	99	99	1	100	21,71	8,94–26,74	98	99	1	99
	C	11,88	2,67–23,97					7,02	3,83–9,78					2,36	0,83–12,80				
Cam16	S	31,22	19,30–38,67	93	97	0,92	76	24,68	18,59–30,37	55	62	0,97	84	28,53	8,62–32,15	57	68	0,99	92
	C	28,51	18,24–35,96					23,26	18,86–29,81					25,33	6,64–29,77				
Cam17	S	31,22	19,30–38,67	96	97	1	94	24,68	18,59–30,37	88	92	1	96	26,53	8,62–32,15	73	88	1	89
	C	20,06	4,7–29,07					6,16	3,64–8,91					4,57	2,65–10,71				

LOBE = Lobelioideae; CYPHI: Cyphioideae; CA-CYA: Campanuloideae-Cyanantheae; CA-WAH: Campanuloideae-Wahlenbergieae. MP_BS = Bootstrap values obtained under the Maximum Parsimony criterion; MP_JK = Jackknife values obtained under the Maximum Parsimony criterion; BI = posterior probability values obtained under Bayesian Inference; ML_BS = Bootstrap values obtained under the Maximum Likelihood criterion; S = Stem node; C = Crown node.

## Results

### Sequence Data

The final alignment of the 680 *petD* sequences (D680), containing 16 outgroups, was 1486 base pairs (bp) long, plus 243 coded indels. The CS-based dataset (D088), with 72 ingroup accessions, was 1239 bp long, plus 138 coded indels. Finally, the PS-based dataset (D101), with 85 ingroup taxa was 1264 bp long, plus 151 coded indels.

### Phylogenetic and Dating Analyses

**Figure 2:**
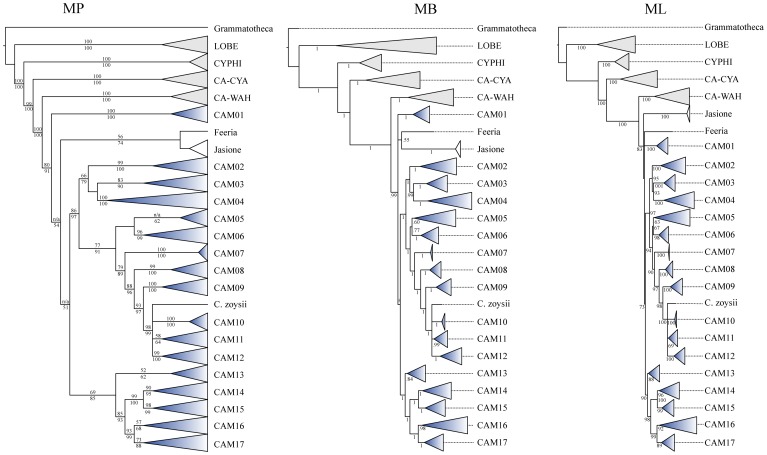
Comparison of the respective phylogenetic trees inferred from the mass sampling (MS, D680), using Maximum Parsimony (MP), Bayesian inference (BI), and Maximum Likelihood (ML). Clades have been transformed into triangles using the "collapse" option in TreeEdit. Gray triangles indicate the respective outgroup and sister clades; blue triangles refer to “Cam” clades containing at least one accession of *Campanula* (Cam01 to Cam17; see text). Numbers below branches are the respective MP-jackknife (MP), posterior probability (BI), and ML-bootstrap (ML) values; numbers above branches are MP-bootstrap values (MP).

Parsimony ratchet analyses performed on the complete dataset (D680) inferred 18852 most parsimonious (MP) trees, with the following metrics: Length (L) = 2503, Consistency Index (CI) = 0.499, and Retention Index (RI) = 0.928 (Fig. 2, Table 1). The total number of interior nodes with a significant bootstrap support (>50%) was equal to 265 (39%). When performed on the reduced datasets D088 and D101 (Figs. S4, S8), parsimony analyses provided MP trees with a greater CI (0.644 and 0.601, respectively) and a higher percentage of resolved nodes (D088∶65%; D101∶76%) compared to the complete dataset. Independent Bayesian analyses (four independent runs keeping 10000 trees per run) of the respective datasets (Figs. S1, S5, S9), performed under the GTR+G+I model of nucleotide substitution, yielded congruent topologies and similar posterior probability (pp) values for each separate 50% majority-rule consensus tree. The proportion of resolved nodes (pp>0.5) varied from 48% (D680) to 73% (D088) and 83% (D101) (Table 1). Maximum Likelihood (ML) analyses performed under the GTR+GAMMA model of sequence evolution (Figs. S2, S6, S10) produced trees with the following scores: D680: -ln = −15871,72173; D101: -ln = −9859,839022; D088: -ln = −8646,156467 (Table 1). The percentage of resolved interior nodes, calculated with the di2multi option and a tolerance value of 10^−4^ in the R package Ape, ranged from 43 (D680) to 73 (D088) and 81 (D101) (Table 1). Overall, the number of interior nodes increased towards the reduced dataset, and for the given dataset, MP reconstruction tended to be more conservative (lower number of supported internal nodes). Furthermore, the drastic reduction of taxa also resulted in a decrease of the proportion of parsimony informative characters, ranging from 41.8% in D680 to 32.0% in D101, and 29.5% in D088.

**Figure 3:**
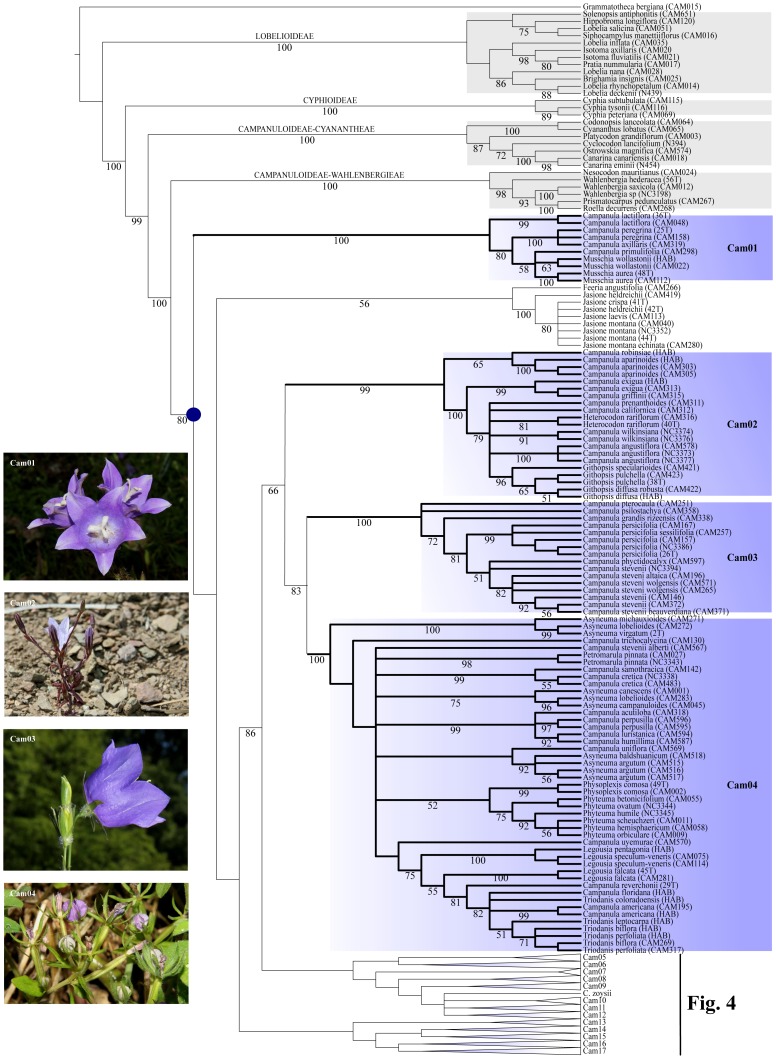
Maximum Parsimony Strict consensus tree of *Campanula* and relatives (D680). Part of the cladogram showing detailed relationships for outgroup and sister lineages, and clades Cam01, *Jasione*-*Feeria*, and Cam02 to Cam04. Values below branches indicate bootstrap support for the sustained clades. Gray boxes indicate the respective outgroup and sister clades; blue boxes refer to “Cam” clades containing at least one accession of *Campanula* (Cam01 to Cam17; see text). A blue dot indicates the crown node of *Campanula* s.lat. Pictures are representative specimens for clades Cam01 (*Campanula primuliifolia*), Cam02 (*Campanula exigua*), Cam03 (*Campanula persicifolia*), and Cam04 (*Legousia falcata*). All photos from Guilhem Mansion.

**Figure 4:**
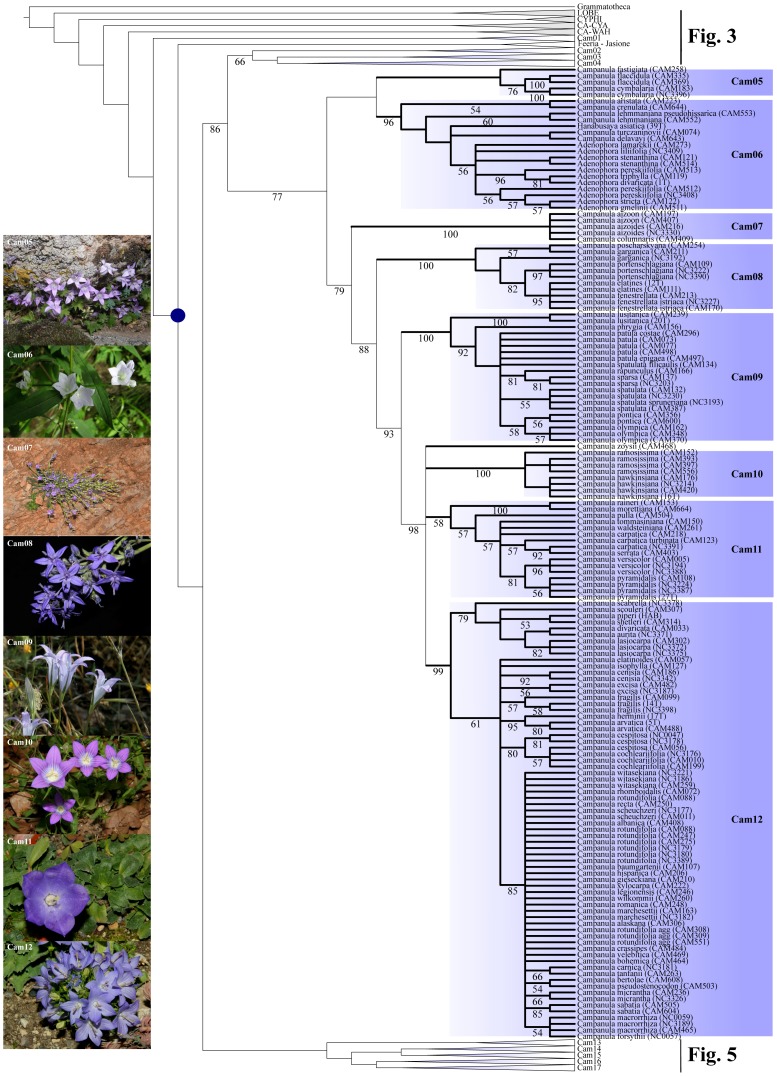
Maximum Parsimony Strict consensus tree of *Campanula* and relatives (D680). Part of the cladogram showing detailed relationships for clades Cam05 to Cam12. Values below branches indicate bootstrap support for the sustained clades. Gray boxes indicate the respective outgroup and sister clades; blue boxes refer to “Cam” clades containing at least one accession of *Campanula* (Cam01 to Cam17; see text). A blue dot indicates the crown node of *Campanula* s.lat. Pictures are representative specimens for clades Cam05 (*Campanula cymbalaria*), Cam06 (*Adenophora stricta*), Cam07 (*Campanula aizoon*), Cam08 (*Campanula fenestrellata*), Cam09 (*Campanula spatulata*), Cam10 (*Campanula ramosissima*), Cam11 (*Campanula raineri*), and Cam 12 (*Campanula Isophylla*). All photos from Guilhem Mansion, except Cam 05 (Nursel Inkici), Cam06 (Si-Feng Li), and Cam07 (Georgia Kamari & Dimitrios Phitos).

**Figure 5:**
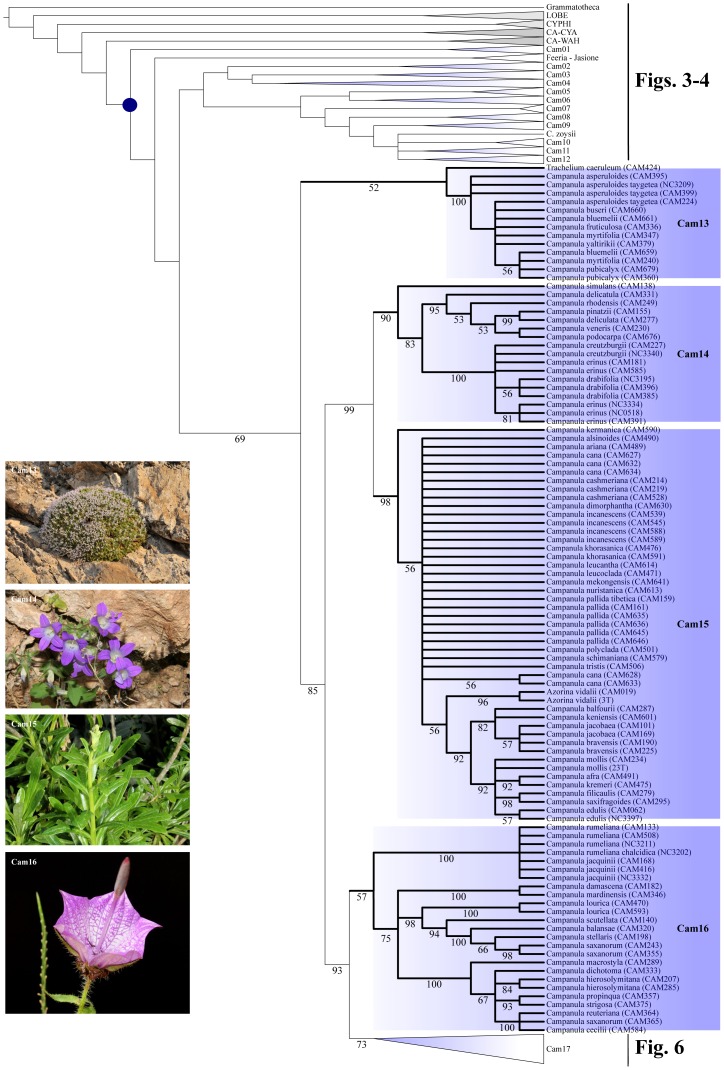
Maximum Parsimony Strict consensus tree of *Campanula* and relatives (D680). Part of the cladogram showing detailed relationships for clades Cam13 to Cam16. Values below branches indicate bootstrap support for the sustained clades. Gray boxes indicate the respective outgroup and sister clades; blue boxes refer to “Cam” clades containing at least one accession of *Campanula* (Cam01 to Cam17; see text). A blue dot indicates the crown node of *Campanula* s.lat. Pictures are representative specimens for clades Cam13 (*Campanula asperuloides*), Cam14 (*Campanula draboides*), Cam15 (*Azorina vidalii*), and Cam16 (*Campanula macrostyla*). All photos from Guilhem Mansion, except Cam13 (Georgia Kamari & Dimitrios Phitos) and Cam16 (Galip Akaydin).

**Figure 6:**
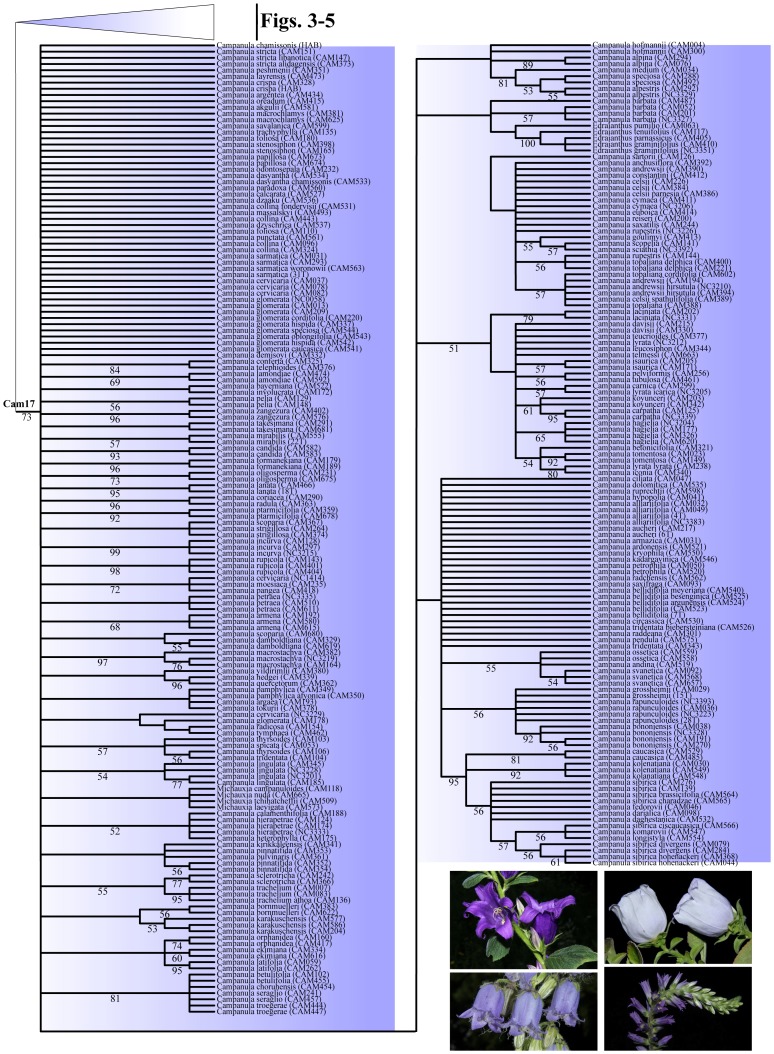
Maximum Parsimony Strict consensus tree of *Campanula* and relatives (D680). Part of the cladogram showing detailed relationships for clade Cam17. Values below branches indicate bootstrap support for the sustained clades. Pictures are representative specimens for clade 17 (clockwise from upper left: *Campanula latifolia*, *C. incurva*, *C. spicata*, and *C. barbata*). All photos from Guilhem Mansion.

For presenting the phylogenetic results, we followed the general structure depicted by the MP analyses of the D680 dataset, and mentioned when necessary the minor discordances to trees obtained with other methods (Figs. 2, 3, 4, 5, 6). The strict consensus tree, rooted with 16 accessions of Lobelioideae and Cyphioideae (*Grammatotheca* chosen as the most external outgroup for the Bayesian inferences), overall depicted sister relationships between a “Wahlenbergioid” clade, including representatives of tribe Wahlenbergieae (*Wahlenbergia*, *Nesocodon*, *Prismatocarpus*, and *Roella*), and a “Campanuloid” clade, comprising all accessions of the respective Campanuleae, Edraiantheae, Jasioneae, Musschieae, Theodorovieae, Peracarpeae, and Phyteumeae (Figs. 2, 3, 4, 5, 6; BS 100). *Campanula* as circumscribed taxonomically was broadly polyphyletic, forming a large *Campanula* s.lat. clade. The latter was arbitrarily subdivided into 17 generally well-supported “Cam” clades containing at least one accession of *Campanula* (Cam01 to Cam17; Figs. 1, 2, 3, 4, 5, 6; Table 2). In four cases (clades Cam05, Cam11, Cam13, and Cam16) BS support for branches was below 60%, with nonetheless corresponding JK values above 62%, and BS values up to 92% in the ML reconstruction. For instance, the bootstrap difference between MP and ML estimates for the respective branches sustaining both Cam13 and Cam16 was 35% (Table 2). The size of the 17 Cam clades showed great variation and ranged from two species in Cam10 (three in Cam05 and Cam07) to some 162 species in Cam17. A *Jasione* – *Feeria* clade was only weakly supported by the MP and BI analyses (BS = 71, JK = 73, pp = 0.57), but not by the ML ones (Fig. 2). Finally, all analyses performed on D088 inferred 15 out of the 17 Cam clades: clades Cam07 and Cam10 were not recovered while clades Cam05, Cam08, Cam11, and Cam14 were monotypic (Table 2). Furthermore, some nodes (Cam16 and Cam17) showed strongly different support values relative to the particular sampling scheme (e.g. D088-Cam16: BS = 93; D101-Cam16: BS = 55; Table 2).

**Figure 7:**
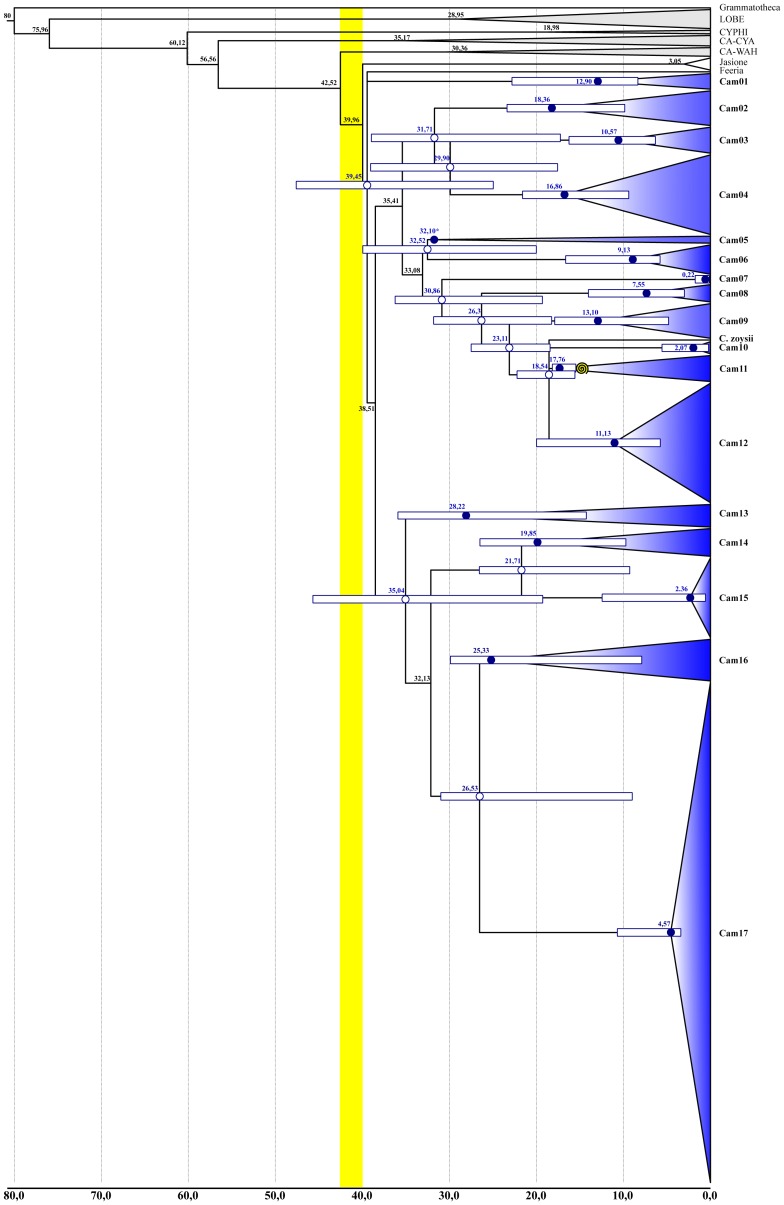
Chronogram of *Campanula* and relatives (D680) inferred from the penalized-likelihood method implemented in r8s, and dated using one fossil constraint (yellow spiral). The yellow box refers to the time span between the stem and crown node of *Campanula* s.lat. Clades are represented by triangles proportional in size to the number of included accessions. Gray triangles indicate the respective outgroup and sister clades; blue triangles refer to “Cam” clades containing at least one accession of *Campanula* (Cam01 to Cam17; see text). White bars represent 95% confidence intervals (CI) for the respective node ages (blue: crow ages; white: stem ages). An asterisk indicates nodes for which CI could not be calculated. Ma = Mega Annuum or Million years; LOBE = Lobelioideae; CYPHI: Cyphioideae; CA-CYA: Campanuloideae-Cyanantheae; CA-WAH: Campanuloideae-Wahlenbergieae.

Divergence time values estimated for the respective stem and crown nodes of selected clades are shown in Table 2 and Figs 7, S3, S7, and S11. In the following, unless otherwise stated, 95% confidence intervals are indicated in brackets after the mean values.

Finally, because the trees inferred for the D088 analyses greatly differed in general topology, branch support, and clade circumscription (see below), the Wilcoxon signed rank test was only performed between D101 and D680 estimates. Both node age and branch support values were found to be significantly different between the two datasets (age estimates: W = -385; P = 0.025; branch support: W = -45, P = 0.009), with lower median estimation for D101.

## Discussion

### Mass versus Classification-guided and Phylogeny Guided Sampling Strategies

The pros and cons of taxon vs. character sampling and its direct impact on the quality of phylogenetic reconstruction has long been debated [23], [27], [51], [52]. In theory, the addition of taxa should enhance the number of potential tree topologies, improve the phylogenetic accuracy, and potentially reduce the effect of long-branch attraction by dispersing homoplasy across the tree. Additionally, when more taxa are sampled, supplementary internal nodes and substitutions can be detected, ultimately improving branch length estimates [23], [25], [26], [53]. In contrast, increasing the number of nucleotides tends to resolve nodes with better statistical support, but with lower phylogenetic accuracy or higher systematic error if the number of taxa is not sufficient [23], [24], [52]. To one extreme, such an approach can dramatically increase support for the wrong topology. Overall, as far phylogenetic accuracy is concerned, empirical studies and simulations tend to support a much greater beneficial effect of increasing taxon sampling over the number of characters.

In this study, we generated a nearly fully sampled taxon set as a reference to evaluate the impact of different reduced sampling strategies on selected parameters including tree topology, branch support, percentage of supported nodes (BS/JK>50), and time estimate (Table 2). The goal was to evaluate the effects of (i) a drastic under-sampling of taxa, and (ii) the qualitative effects of two small datasets different in composition but similar in size (D086 and D101).

When evaluating the effects of under-sampled datasets, we found that the MS-based dataset (D680) produced more trees with a smaller proportion of supported internal nodes (D680: BS 39, JK 44; D101; BS 76, JK 78; D088: BS 65, JK 66) and a greater number of homoplasies (D680: CI = 0.499; D101; CI = 0.601; D088: CI = 0.644). As far as the different composition of reduced data sets was concerned, two significantly different trees were inferred. On the one hand, the classification-guided sampling failed to recover all 17 major *Campanula* clades, and gave a different tree shape with a very heterogeneous representation of lineages when compared to the MS-based approach. Indeed, no support or time information could be inferred for 6 crown nodes (Cam05, Cam07, Cam08, Cam10, Cam11, Cam14; Table 2) because the clades were either lacking or resolved as monotypic. To the contrary, a large number of the included type species (38%) of various supraspecific taxonomic entities appeared in the otherwise unresolved clade Cam17 (Figs. 6, S4, S5, S6, S7). Furthermore, the topological differences also had limiting effects on branch support calculation or age inference, and overall prevented direct statistical comparisons between D086 and D680 (see Results). For instance, in the CS-based analysis Cam16 contained only two species (*C. rumeliana* and *C. jacquini*), and is well-supported (BS 100). In the MS-based reconstruction, Cam16 is different in composition (16 species), and hardly supported (BS 57). Thus, despite a nearly complete inclusion of type taxa above the species rank (42 type species for respective sections and subgenera) the CS-based approach inferred a biased tree topology, overall suggesting strong homoplasy among morphological characters and their states. This should be tested by adding characters to a multi-gene data set that could better approximate the organismic phylogeny and by the development of a corresponding morphological matrix. However, our results have also further implication on the use of morphogenera as “natural” evolutionarily predictive units in biodiversity analysis and macroecology. While there is a recent, unresolved debate in zoology [54], [55], case studies in plants are largely unavailable. The biased tree resulting from the CS-based approach as well as the high polyphyly of *Campanula* confirmed by mass sampling provides a striking example that angiosperm morphogenera as currently used may not be good entities. *Campanula* may in fact just exemplify the tip of an iceberg, underscoring the need of efficient phylogenetic tools to include as many species and genera as possible in future attempts to base biodiversity studies on evolutionarily more meaningful units.

On the other hand, the PS- and MS-based analyses generated similar topologies, but with statistically different branch support (P = 0.009) and age estimates (P = 0.025). On the whole, large taxon-sampling produced an important accumulation of new branches in the phylogenetic tree, resolving clades with better circumscription and branch support Nevertheless, this approach also resulted in the increase of accessions with highly similar or identical sequences, eventually forming large polytomies (e.g. clades Cam12 and Cam17). The presence of such unresolved clades however can also be the reflection of particular biological events, including reticulate evolution or rapid diversification of lineages [56], [57], [58], [59], whose detection is of essential interest for the comprehension of such a large group of plants.

To conclude, our current approach favoring mass taxon sampling with a single efficient marker already allowed an important increase of the phylogenetic accuracy of the investigated group. Indeed, the large and polyphyletic genus *Campanula* is here subdivided into 17 major clades that will be discussed in more detail below. Our analyses also depicted species-rich and phylogenetically unresolved groups, along with unbalanced sister clades, overall opening new doors to more evolutionary-oriented studies. To better understand the evolutionary diversification at the species level and also to thoroughly revise their taxonomy by evaluating alpha species concepts, each of these major clades will certainly constitute a study group that can be independently worked on.

### A Comprehensive Phylogenetic Framework as a Basis for Evolutionary Studies and Species Diversity Assessment in Campanula and Allies

In this part of the discussion, unless further noticed, we refer to the more conservative MP-based topology and corresponding bootstrap support values for branches (BS). Chromosome numbers mainly follow Lammers’ compilation [3]. Age estimates for branches at respective stem (S) and crown (C) nodes, and corresponding 95% confidence intervals, are based on the r8s results for the complete dataset (D680). Keeping in mind that those inferred values are minimum ages with sometimes large confidence intervals, we cautiously provide in the following discussion tentative hypotheses concerning the origin and diversification of the respective *Campanula* clades.

#### * Clade cam01 (S: 39,45 Ma [24,81–47,91]/C: 12,90 Ma [8,37–22,68])

This well-supported clade (BS 100, JK 100; Fig. 3; Table 2) comprises two out of three species of the Madeiran endemic *Musschia* [60], and four of *Campanula*, namely *C. axillaris*, *C. lactiflora*, *C. peregrina*, and *C. primulifolia*. This so-called "Musschia clade" was early depicted by Eddie et al. [20], and includes here one additional species endemic to Turkey (*C. axillaris*). Our *petD* data strongly favor sister relationships between *C. axillaris* and *C. peregrina* on the one hand, and between *C. primulifolia* and *Musschia*, on the other. The latter relationship is congruent with the *trnLF* signal [13], and depict interesting geographical links between the eastern and western Euro-Mediterranean area. Dating analyses further suggest that the estimated time of divergence between *C. primulifolia* and *Musschia* (c. 9 Ma [2.82–11.82]) overlaps with the time span of the volcanic island archipelago emergence, starting c. 15 Ma [61], and possibly favors a neoendemic origin for *Musschia* [62]. Interestingly, despite the acquisition of striking new vegetative and floral features in the insular neoendemic [63], the single dispersal of *Musschia* common ancestor was not followed by episodes of intensive diversification, as often observed in volcanic islands [64]. Alternatively, potential episodes of extinctions could have erased an early occurring radiation in *Musschia*.

From a taxonomic point of view, our data do not support the inclusion of both *C. peregrina* and *C. primulifolia* in *Echinocodonia*, as suggested by Kolakovskii [65]. Furthermore, karylogical evidence also contradicts such a combination, with *C. peregrina* having n = 13 and *C. primulifolia*, n = 18. Overall, the great morphological and cytological diversity (*C. lactiflora*: n = 17, 18; *Musschia aurea*: n = 16) found in this geographically widespread clade, with overall rather low diversification on oceanic islands, could suggest active episodes of extinction during the last ten million years. More detailed analyses, using likelihood-based biogeographic methods [66] and lineage through time inference should be performed to test such hypotheses.

#### * Clade cam02 (S: 31,71 Ma [16,72–38,53]/C: 18,36 Ma [9,89–23,68])

This strongly supported clade (BS 100, Fig. 3, Table 2) contains 12 species of North American distribution, seven of them being annual (*Githopsis diffusa*, *G. pulchella*, *G. specularioides*, *Heterocodon rariflorus*, *C. angustiflora*, *C. griffinii*, *C. exigua*), and five perennial (*C. aparinoides*, *C. californica*, *C. prenanthoides*, *C. robinsiae*, and *C. wilkinsiana*). In our analyses, *C. robinsiae*–*C. aparinoides* form a first diverging clade, while *C. exigua*–*C. griffinii* is sister to a last clade including all remaining taxa. The *petD* topology is by large congruent with smaller clades obtained from combined cpDNA analyses that included either six [21] or eight species [67]. Our results, nonetheless, do not support the inclusion of *C. scouleri* in this clade [21], [67] a fact that could be better interpreted as a misidentification between *C. scouleri* and *C. prenanthoides*, both species having somewhat similar corollas.

Interestingly, three out of the four bell-flowers endemic to California (the rare *C. sharsmithiae* from the Shasta Mountains of North California is missing), all annuals, morphologically similar, and with strong affinities to serpentine soils, do not form a clade. Indeed, further cytological and palynological data also support the genetic separation between *C. angustiflora* (n = 15; 6-porate pollen) and the *C. exigua*–*C. griffinii* clade (n = 17; pantoporate pollen) [68]. *Campanula angustiflora* is embedded in an internally rather unresolved clade otherwise comprising both slender, chiefly cleistogamous, and xerophytic annuals (*Githopsis* and *Heterocodon*), along with more shade-tolerant, chasmogamous perennials (*C. californica*, *C. prenanthoides*, and *C. witasekiana*).

Overall, the origin of the American clade Cam02 can be inferred in the Early to Middle Oligocene (32.91 Ma [19.09–38.91]), and current lineages started to diverge in the Early Miocene (c. 20.45 Ma [11.49–25.76]). It seems premature, without rigorous biogeographic reconstruction to conclude to either a single long distance dispersal event or a more progressive series of geodispersal events from Eurasia to the Americas.

#### * Clade cam03 (S: 29,90 Ma [16,72–38,53]/C: 10,57 Ma [6], [15]–[16], [53])

This clade, generally undervalued by recently published phylogenetic trees (up to three species in Roquet et al. [13]), shows strong support for the crown group (BS 100; Fig. 3) and presently contains six species and 10 subspecies of bluebells occurring in the Asian part of Turkey and Caucasus, *C. persicifolia* extending its range to central and southern Europe. Except for the two early diverging biennials *C. psilostachya* and *C. pterocaula*, all species in this clade are perennial. *Campanula psilostachya* is a Turkish endemic that was at some time of its taxonomic history included in *Asyneuma*, based on its small funnel shaped corolla with divided lobes [6], or considered to be morphologically related to *C. americana* [69]. It presently resides in clade Cam03 so that both hypotheses are not supported by the current gene tree, which rather suggests strong relationships with *C. pterocaula*, another Turkish species with broadly campanulate flowers. The attractive species *C. persicifolia* and *C. latiloba* also share large campanulate corollas, and mainly differ by the cauline leaf width (linear in *C. persicifolia* vs. broadly lanceolatate in *C. latiloba*), the capsule dehiscence mechanism (apical in *C. persicifolia* vs. median in *C. latiloba*) and the size of their distribution range. While *C. persicifolia* is widely distributed throughout Europe, *C. latiloba* is a Euxine element of Turkey. Both species are frequently cultivated in gardens. The use of *C. persicifolia* as an ornamental plant dates back to the 16^th^ century [69]. Our analysis further depicts strong sister relationships between *C. stevenii* (4 subspecies included) and *C. phyctidocalyx*, both species with usually one-flowered ascending-erect stems, a long ribbed calyx and a funnel-shaped, moderately-sized corolla, differing only by the ovary shape. Interestingly, two additional subspecies of *C. stevenii* (subsp. *albertii* and subsp. *turczaninovii*) fall in the respective clades Cam04 and Cam06, overall suggesting the polyphyly of *C. stevenii* in its current concept.

#### * Clade cam04 (S: 29,90 Ma [16,72–38,53]/C: 18,86 Ma [9,49–21,97])

This large and well-supported clade (BS100, Fig. 3, Table 2) is quite unresolved and includes seven campanuloid genera and 11 species of *Campanula*. Overall, this group can be considered a large paraphyletic *Asyneuma*, with two early diverging *Asyneuma* lineages, respective the unresolved *A. michauxioides*–*A. lobelioides*–*A. virgatum* clade, and the monotypic *A. trichocalycina* clade, and a third group with low support (BS 52) containing remaining accessions of *Asyneuma* plus other genera. Within the last clade, some particular assemblages are further delimited with confidence, including e.g. a disjunct European/American clade encompassing *Legousia*, *Triodanis*, and three species of *Campanula* (BS 81), a mostly Iranian clade containing *C. acutiloba*, *C. humillima*, *C. luristanica*, and *C. perpusilla* (BS 100), depicted for the first time, or *the C. samothracica*–*C. cretica* clade (BS 100).

*Asyneuma* is a group of mostly perennial, robust and erect herbs with deeply divided corollas, ranging from SE Europe to E Asia, most of the specific diversity being encountered in the Middle-East [3], [70]. While the inclusion of *Asyneuma* in a paraphyletic *Campanula* has been long established [12], [13], its polyphyly is suggested here for the first time. Indeed, the most detailed study so far done for that group [71], including eight species of *Asyneuma*, overall supported a monophyletic genus by transferring the problematic *A. comosiforme* into *Campanula*.

The geographically disjunct *Campanula*–*Legousia*–*Triodanis* clade shows a paraphyletic genus *Legousia* with respect to a derived North American clade, overall suggesting a single dispersal to the Americas from a *Legousia*-like Mediterranean ancestor during the Late Miocene (11,78 Ma [4,71–14,63]). This single introduction was quickly followed by the diversification of several lineages now represented by *Campanula* (incl. *Campanulastrum*), and *Triodanis*. Close relationships between the annual taxa of *Legousia* (4 species) and *Triodanis* (6 species) have long been suggested, the two genera being sometimes merged due to the scarcity of segregating morphological differences, including the degree of stem branching or the corolla shape [72], [73] or some similarities in chromosome numbers (x = 7, 8, and 10 present in both *Legousia* and *Triodanis*). Our results largely support and amend recent works [21], [67] that inferred a similar Eurasian - American disjunction (but without age estimates), and further show the lability of the respective annual and perennial conditions in the campanuloids. In the present case, the annual condition observed in both *Legousia* and *Triodanis* shows reversals to the perennial condition in the rare endemics *C. reverchonii* of Texas and *C. floridana* of Florida, or the Eastern North American *C. americana*. Mediterranean/American disjunct patterns have been exemplified for other plant groups, including the Betoideae, the mostly annual Chironiinae (Gentianaceae), *Lithospermum* (Boraginaceae), *Lotus* or *Lupinus* (Fabaceae) [74], [75], [76], [77], [78], [79].

Another Eurasian-American pattern can also been observed between a Himalayan *Asyneuma argutum* clade (two subspecies) and the circumboreal-American *Campanula uniflora*, the two entities having diverged in the Late Miocene (7.60 Ma [2.64–11.22]; Fig. S3). Also weakly supported by the *petD* reconstruction, the position of *C. uniflora* into an *Asyneuma* lineage has been inferred by other studies [67], [71].

The strongly supported, mostly Iranian clade *C. acutiloba*–*C. humillima*–*C. luristanica*–*C. perpusilla* (BS 100) encompasses morphologically similar species, mostly separated by inconspicuous morphological traits [80]. Indeed, the sister clade *C. luristanica*–*C. humillima* denote strong genetic relationships between two species sometimes considered varieties of each other’s. In the same way, the rare *C. hermanii*, just known from the type locality, is morphologically separated from *C. humillima* by the presence of sub-succulent leaves, a quite labile character. Overall, the three last-mentioned “species” could represent only one, and reflect potential taxonomic redundancy.

Finally, clade Cam04 contains three Aegean endemics, *C. cretica*, *C. samothracica*, and *Petromarula pinnata*. The sister relationships between *C. cretica* and *C. samothracica*, sometimes considered as subspecies, are depicted here for the first time. Our data suggest a Miocene origin for this clade (14,24 Ma [8,19–17,02]), followed by a Pleistocene diversification (0,62 Ma [0,02–3,08]), overall suggesting very recent arrival of *C. cretica* in Crete. Recent studies [19], only including the Cretan endemic, inferred a putative age of 24 (±10) Ma for the *C. cretica* lineage, advocating that “this species represents another continental remnant that has not diversified in isolation”. At last, the phylogenetic position of *Petromarula*, which has been considered a sister lineage to the *Phyteuma*–*Physoplexis* clade, but with low support [19], is unresolved using *petD* sequences. This genus was first segregated from *Phyteuma* owing to the unique presence of pinnate leaves, quasi-absence of pollen collector hairs, and a showy club-shaped stigma.

#### * Clade cam05 (S: 32,52 Ma [20], [37]–[40], [35]/C: 32,10 Ma [n/a])

This low-supported clade (BS 66, Fig. 4, Table 2), found here for the first time, contains two annual species, namely *C. fastigiata*, ranging from Mediterranean Africa to Caucasus, and *C. flaccidula* from Middle-East, and the perennial *C. cymbalaria*, occurring in Greece (Chios island), Lebanon, and Turkey [81]. *Campanula fastigiata* was also described under either *Brachycodon* or *Brachycodonia* [14] to reflect potential morphological transition between *Campanula* and *Legousia*, an assumption not reflected by the present gene tree. In fact, *C. fastigiata* is inferred to be sister to a more eastern Mediterranean lineage, suggesting some potential W to E evolutionary patterns. The disparity in chromosome numbers found in the extant species, with 2 n = 18 (*C. fastigiata*), 28 (*C. flaccidula*), and 34 (*C. cymbalaria*), along with the presence of long phylogenetic branches sustaining the current clades, and the rather ancient age inferred for the whole lineage (32.52 Ma [20], [37]–[40], [35]), would also support strong variation in respective rates of speciation/extinction in that clade, a hypothesis that needs to be further tested. High levels of extinction could potentially explain the current disjunct distribution of *C. fastigiata* in both western and eastern Mediterranean regions. Finally, the present clade also supports a new switch from the annual to perennial condition, a rather common episode in *Campanula* evolution [82] the potential causes of which would deserve more investigations.

#### * Clade cam06 (S: 32,52 Ma [20], [37]–[40], [35]/C: 9,13 Ma [5,70–17,48])

This well-supported clade (BS 98, Fig. 4, Table 2) contains seven representatives of the Asian genus *Adenophora* (Asia), the monotypic *Hanabusaya* of Korea, and six bellflowers, most of them occurring in China and surrounding areas. The whole assemblage is largely paraphyletic with an otherwise monophyletic *Adenophora* (BS 67). Nonetheless, early study on Campanulaceae based on ITS sequence data [20] inferred a paraphyletic *Adenophora* (11 species included) to *Hanabusaya*, a hypothesis in some way supported by morphological evidence. Indeed, both genera share campanulate flowers with very prominent nectaries, and nodding, basally opening capsules [83]. Our current sampling of *Adenophora* is somewhat limited, the genus containing some 67 species [3], and diverge qualitatively from the aforementioned study, thus precluding conclusive remarks on potential cases of incongruence between the respective maternally and bi-parentally inherited molecular markers.

This well- resolved clade shows an early diverging lineage including *Campanula aristata* (Afghanistan to China) and *C. crenulata* (China), two high elevation plants occurring in alpine meadows or thickets. Morphologically, *C. crenulata* approaches *C. delavayi*, another Chinese species more frequent in pine forests, whose sister relationships with *C. stevenii* subsp. *turczaninovii* is poorly supported. The latter taxon mainly differs from other subspecies of *C. stevenii* by its chromosome number (2 n = 34 vs. 2 n = 32). Finally, both subspecies of *C. lehmanniana* (subsp. *lehmanniana* and subsp. *pseudohissarica*), from Kirgizstan and Tadzhikistan, are genetically similar, but their relationships with respect to other species of this clade remain poorly resolved.

#### * Clade cam07 (S: 30,86 Ma [18,58–35,81]/C: 0,22 Ma [0,02–1,69])

This strongly supported monophylum, exemplified here for the first time, is early diverging and sister to the respective Cam08–Cam12 assemblages (BS 100, Fig. 4, Table 2). *Campanula aizoides*, *C. aizoon*, and *C. columnaris* are three narrow-distributed, Greek endemic species, morphologically similar and characterized by their robust taproot, dense rosette of leaves, from which arises a thyrsoid inflorescence with large, tubular-campanulate flowers [84]. *Campanula aizoides* presents a striking bi-regional and disjunct distribution in western Crete (Lefka Ori) and northern Peloponnese (Mt Chelmos), whereas *C. aizoon* (Mts Parnassos and Giona) and *C. columnaris* (Mt Vardhousia) are found in some places of the mountain ranges of Central Greece (Sterea Ellas). The divergence age estimate at the lineage stem node is 30,86 Ma [18,58–35,81]), indicating an ancient separation of this Greek lineage from the Cam08–Cam12 sister clade. Interestingly, the whole lineage seem to have diversified very recently (c. 1.5 Ma), forming two mainland lineages and an insular one, contradicting a paleo-subendemic status postulated for the Cretan *C. aizoides* [19]. Alternatively, the three species could represent a single entity of an older lineage whose remnant populations in both mainland Greece and Crete may have escaped from extinction by taking refuge in and/or adapting to mountain habitats. Overall, the low genetic distances estimated for the respective taxa, the identical chromosome numbers (n = 8), weak morphological differences, and different ecological preferences [84] would better favor the second hypothesis.

#### * Clade cam08 (S: 26,30 Ma [18,35–31,67]/C: 7,55 Ma [3,29–14,73])

This well-supported monophyletic group (BS 100, Fig. 4, Table 2) contains five “isophyllous” species of *Campanula*, namely *C. garganica*, *C. elatines*, *C, fenestrellata*, *C. portenschlagiana*, and *C. poscharskyana*. The *Isophylla* group is morphologically (isophylly, both the basal and cauline leaves having cordate to ovate blade; erect capsules opening with basal pores) and karyologically (2 n = 34) well defined, and encompasses some 12 species disjunctly distributed in the sub-Mediterranean Adriatic Mountains [22], [85], [86]. *Isophylla* has been further divided into three morphological groups [87], and corresponding three well-supported, albeit non-sister ITS clades [22]. Our study also inferred the polyphyly of the isophyllous assemblage with Cam08 corresponding to the tentative “garganica” clade of Parks et al. [22], their “fragilis” and part of the “elatines” clades being imbedded in our Cam12 lineage (see below).

Despite great similarities between the respective *petD* (this study) and ITS [22] inference, some taxa show strongly incongruent topological position. Indeed, our current *petD* analysis does not support the sister relationships between *C. elatines* and *C. elatinoides*, the former being sister to *C. fenestrellata* and the latter included in clade Cam12, a result congruent with Borsch et al. [18]. The “elatines” group, treated under “garganica” by Damboldt [85], was described to encompass two narrowly-distributed alpine species (*C. elatines* and *C. elatinoides*), characterized by intermediate morphological characters between the “fragilis” and “garganica” clades [22]. Interestingly, isozyme evidence [88] support closer relationships between *C. elatinoides* and *C. isophylla* (fragilis clade), a result in line with our current inference (*C. elatinoides* and *C. isophylla* in clade Cam12). Furthermore, some ecological differences, including the strong affinity of *C. elatines* (Piemont) for gneiss or granite versus calcareous rocks for *C. elatinoides* (Insubrian Alps), would add further support for their phylogenetic divergence [22].

On the whole, Cam08, as currently circumscribed, is a genetically well-supported clade with strong morphological, karyological, and geographical structure. Indeed, most species are similar in habit and floral shape, share a diploid to hexaploid chromosome number based on x = 17, and mainly occur in the Transadriatic Mediterranean area.

#### * Clade cam09 (S: 23,11 Ma [18], [18]–[28], [16]/C: 13,10 Ma [4,60–17,55])

This clade shows high support for branches (BS 100; Fig. 4, Table 2) and contains 8 species (11 subspecies) with similar chromosomal valence (most derived from x = 10). Close relationships between *C. patula* (2 n = 20, 40), a species widespread in European woodlands and meadows, and the East-Mediterranean perennial geophyte *C. spatulata* (2 n = 20) were first revealed by Borsch et al. [18], within their *Campanula rotundifolia*-clade. The current increased sampling of Mediterranean species, such as the annual *C. lusitanica* (2 n = 18, 20), *C. phrygia* (2 n = 16), and *C. sparsa* (2 n = 20), and the biennial-perennial *C. olympica* (2 n = 20), *C. pontica* (2 n = n/a), and *C. rapunculus* (2 n = 20), reveals sister relationships between *C. lusitanica* and the rest of the species, a pattern supported by a more detailed ITS-based phylogenetic study [89]. Cano-Maqueda et al. [89] further included five annual, Iberian native species, which formed a well-supported clade including *C. lusitanica*, and sister to a *C. rapunculus*–*C. sparsa*–*C. patula* lineage. Surprisingly, *C. lusitanica* was inferred as sister to a *C. elatines*–*C. elatinoides* clade by the ITS study of Park et al. [22], a relationship not supported here. Discrepancies between the respective cp- and nrDNA based signals in this clade would deserve further studies.

Within the *C. lusitanica* sister clade, ML reconstruction moderately support sister relationships (BS 59; Fig. S2) between *C. phrygia* (2 n = 16) and the rest of the species (2 n = 20), overall suggesting some episodes of descending dysploidy in the lineage. Morphologically, *C. phrygia* shows some affinities with *C. sparsa*, both species sharing characteristic ribbed capsule opening by three apical to median pores [70]. Phylogenetic inference also moderately supports (BS 60) affinities between the northern Anatolian species *C. pontica* and *C. olympica*. The relationships between *C. patula* (3 subspp.) and *C. spatulata* (3 subspp.) remain unresolved. The origin of the Cretan endemic *C. spatulata* subsp. *filicaulis* was recently estimated to 17 (±8) Ma for a reduced *C. lusitanica*–*C. spatulata* subsp. *filicaulis* clade [19], The current study would support similar age for the divergence between *C. lusitanica* and its sister clade (13.10 Ma [4,60–17,55]), but a much younger origin for the *C. spatulata–C. filicaulis* lineage (stem node 8.60 Ma [1,14–12,90]), overall suggesting a more recent dispersal event in *C. spatulata* from the mainland to Crete, after the isolation of Crete, such as the very recent split between *C. erinus* and *C. creutzburgii* discussed under clade Cam14 below.

#### * Clade cam10 (S: 18,54 Ma [16,50–21,83]/C: 2,07 Ma [0,04–6,49])

This strongly-supported clade (BS 100; Fig. 4, Table 2) contains only two species, namely the annual *C. ramosissima* and the perennial *C. hawkinsiana*, recently included in the newly-described section *Decumbens* [90]. Based on ITS sequence data, Cano-Maqueda and Talavera [90] inferred a moderately-supported “Decumbens” clade (BS 67) showing sister relationships between the respective species pairs *C. decumbens*–*C. dieckii* (not included in the present study, both species treated as synonyms by Lammers [3]) and *C. ramosissima*–*C. hawkinsiana*. Morphologically, the four species share a similar general habit along with a glabrous style surmounted by three erect stigmas, an unusual character for *Campanula* [90]. Caryologically, the group remains rather variable with respective somatic chromosome numbers of 2 n = 20 (*C. ramosissima*), 22 (*C. hawkinsianaI*), 28 (*C. dieckii*), and 32 (*C. decumbens*) [90], [91], [92]. If confirmed by further molecular data, this clade would exemplify a new case of a lineage with current W-E disjunct distribution, with a *C. decumbens*–*C. dieckii* clade of annuals, endemic to the Iberian Peninsula, and a *C. ramosissima*–*C. hawkinsiana* clade occurring in the Eastern Mediterranean region.

#### * Clade cam11 (S: 18,54 Ma [16,50–21,83]/C: 17,76 Ma [16,50–18,27])

Moderately supported (BS 59; Fig. 4, Table 2), this clade contains a mixture of species assigned to either the “isophylloid” group, e.g. *C. morettiana*, *C. pyramidalis*, *C. tommasiniana*, *C. versicolor*, and *C. waldsteiniana*, or to the “rapunculoid” group, e.g. *C. carpatica*, *C. pulla*, *C. raineri*, and *C. serrata*. The isophylloid group encompasses morphologically intermediate taxa that either resembles members of section *Heterophylla* or section *Isophylla*, with occurrence of lateral and sterile shoots, heterogeneous leaf-blades (*Heterophylla*), mostly rotate corollas, and erect capsules (*Isophylla*) [85], [93].

The current *petD* inference depicts a clade somewhat congruent in topology with the ITS reconstruction of Park et al. [22]. A first diverging and strongly supported *C. morettiana*–*C. raineri* group (BS 99) indicates important genetic affinities between otherwise morphologically distinct species. Relationships between *C. waldsteiniana* and *C. tommasiniana*, early suggested by Damboldt (1965), and supported by Park et al. [22], do not find support in the *petD*-based phylogeny (Fig. 3). Finally, *C. carpatica* appears to be polyphyletic, and does not form a clade with *C. pulla*, as weakly suggested by the aforementioned ITS reconstruction (BS 53). Overall, despite similar chromosome numbers based on an x = 17 series, the morphological and phylogenetic circumscription of Cam11 still remains moderate, advocating for more detailed studies aimed at inferring potential synapormorphies for the respective isophylloid and rapunculoid groups.

#### * Clade cam12 (S: 18,54 Ma [16,50–21,83]/C: 11,13 Ma [5,85–14,91])

This well supported clade (BS 99; Fig. 4) corresponds to an enlarged version of the “*C. rotundifolia* clade” sensu Borsch et al. [18], and comprises two main entities. A first subclade (BS 79) with seven North American species of bellflowers is sister to a second large subclade (BS 61), encompassing the so-called “*C. rotundifolia* aggregate” or “alliance”, or section *Heterophylla* [86], [94].

Within the first subclade (BS 79) all species but *C. lasiocarpa* (trans-pacific distribution) are North American endemics. The composition of this group matches the “Rapunculus 1a clade” of Wendling et al. [67], to which the rare *C. shetleri* must be included. Despite some karyological homogeneity, most investigated species sharing a somatic number of 2 n = 34, the subclade appears morphologically heterogeneous. Nonetheless, a clade with low support for branches (BS 53) was depicted to comprise *C. piperi* and *C. shetleri*, two perennial species with more or less dentate margins of the mucronate leaves, occurring in alpine habitats of the northern California - southern Washington mountain ranges. More detailed biogeographic analyses remain necessary to understand the origin of this American clade, whose ancestor was hypothesized to have colonized the New World via the Beringian route [67].

The second subclade (BS 61; Fig. 4) includes most species assigned to section *Heterophylla* [95], a particular group of long-recognized campanulas (harebells) morphologically characterized by the presence of dimorphic leaves, with reniform and petiolate basal leaves and subsessile linear cauline ones, and a basal dehiscence of the capsule [4], [6], [14]. Phylogenetically, the subclade encompasses up to eight lineages, most of them monospecific, and unresolved with each other. A majority of these lines includes dwarf mountain species, morphologically well-circumscribed such as *C. cenisia*, *C. excisa*, *C. cespitosa*, and *C. cochleariifolia*, the latter two inferred as sister species (BS 82). Of interest is the presence in this subclade of some isophyllous species such as *C. elatinoides*, *C. fragilis*, and *C. isophylla*, as already mentioned under clade Cam08. From a taxonomic point of view, the presence of *C. isophylla* in the *Heterophylla* clade can render problematic the distinction of potential isophyllous and heterophyllous groups.

Finally, a large and well-supported subclade contains c. 23 species related to *C. rotundifolia*, which cannot be segregated based on *petD* phylogenetic reconstruction alone. Several explanations can be proposed to explain such polytomy. First, polyploidy is known to occur in this otherwise well-delimited karyological group (x = 17), some species exhibiting up to 6x valence levels, overall rendering the specific limits difficult to assign [96], [97]. Further, most *Heterophylla* species show great distributional range overlap thus increasing the likelihood of genetic exchanges via introgression or homoploid/polyploid hybridization. Last but not least, the inferred crown age of that clade (1,01 Ma [0,32–3.29]) suggest very recent diversification, and does not rule out the possibility of incomplete lineage sorting between clades. Taken as a whole, these evidences explain both the phylogenetic and taxonomic confusion in section *Heterophylla* and particularly *C. rotundifolia*, a species for which some 96 heterobasionyms have been published [3].

Overall, this subclade should be considered a large polyploid complex similar to the many ones exemplified in both the Mediterranean and Arctic-Alpine regions of Europe, including e.g. *Centaurium*, *Draba*, or *Primula* [98], [99], [100], [101], the detailed study of which would imply particular analytical strategy [102].

#### * Clade cam13 (S: 35,04 Ma [19], [21]–[42], [54]/C: 28,22 Ma [13,92–35,88])

This poorly supported clade (BS52; Fig. 5) shows sister relationships between one member of *Trachelium* (*T. caeruleum*) and seven species of *Campanula* (*C. asperuloides*, *C. bluemelii*, *C. buseri*, *C. fruticulosa*, *C. myrtifolia*, *C. pubicalyx*, and *C. yaltirikii*), all species sharing capitate inflorescences, narrow-infundibuliform corollas, and similar chromosome numbers (2 n = 34). Based on such combination of characters, some authors suggested to either include those campanulas into *Trachelium* [103] or to establish new genera such as *Diospharea* or *Tracheliopsis* [104]. Damboldt [15] questioned the separation of these genera from *Campanula* and finally put all these species into synonymy of *Campanula* section *Tracheliopsis*. The current phylogenetic hypothesis does not support either the generic or sectional delimitation, otherwise suggesting the separation of this group of species into two different lineages (Cam13: *C. asperuloides*, *C. buseri*, *C. myrtifolia*, *C. pubicalyx*; Cam16: *C. rumeliana*, *C. jacquinii*). The suggestion of Borsch et al. [18] to restrict *Trachelium* to the one or two species (i.e. following Lammers [3]) would imply to give a separate name to the current sister clade, and by extension to most of the clades described in this study.

#### * Clade cam14 (S: 21,71 Ma [8,94–26,74]/C: 19,85 Ma/[9,76–26,18])

This well-supported clade (BS90; Fig. 5, Table 2) nearly entirely encompasses the subgenus *Roucela* Dumort., a group of 12 small dichotomously branched annual species lacking calyx appendages, and showing disc-like capsules opening by three valves [105]. However, the inferred clade does not contain *Campanula scutellata*, a Balkan native species differing from all the remaining taxa by its large habit size and broad corolla. The placement of *C. scutellata* into *Roucela* has been questioned [105], but potential affinities with annuals of the subgenus *Megalocalyx* (see Cam16 below) have never been suggested. Other than *C. scutellata*, most *Roucela* species are endemic to narrow areas of Greece, the Aegean, and W Turkey, except the widespread, self-compatible *C. erinus* distributed throughout the Mediterranean Basin, from Macaronesia to Iran.

Clade Cam14 can be further divided into three lineages, with an early diverging *Campanula simulans* sister to two subclades, a general pattern congruent with a previous study by Roquet (unpublished thesis). *Campanula simulans* (2 n = 28) has been proposed by Carlström [105] to describe a Turkish species morphologically and cytologically related to *C. drabifolia* (2 n = 28) from southern Greece. Nonetheless, molecular data do not support sister relationships between these two species, *C. drabifolia* belonging to a well-supported subclade (BS 100) otherwise encompassing the Cretan endemic *C. creutzburgii* and the widespread *C. erinus*. The timing of diversification for this subclade (0.87 Ma [0.31–2.85]; Fig. S3) is congruent with the previous study by Cellinese et al. [19], who also inferred a recent split of 2.5±2 Ma between *C. erinus* and *C. creutzburgii*, suggesting a recent dispersal event from the mainland to Crete during the Pleistocene, after the isolation of Crete.

A second subclade (BS 95; Fig. 5) comprises five species with very narrow distributions, namely *Campanula delicatula* (SE Aegean, SW Turkey), *C. rhodensis* (endemic to Rhodos), *C. pinatzii* (endemic to Kasos, Karpathos, and Saria), *C. veneris* (endemic to Cyprus), and *C. podocarpa* (Aegean Islands and SW Turkey and Cyprus). The last two species are poorly resolved as sister lineages (BS <50; JK 52), *C. podocarpa* differing from other species of the subclade by its non-stellate calyx, and some particular edaphic affinities (serpentine tolerant). Interestingly, populations from Cyprus have been recently rediscovered (R. Hand, personal communication), and are genetically close to the Turkish accessions included here (G. Mansion, unpublished data). Species delimitation in this group is not easy [105], and some morphs cannot be identified properly (G. Parolly and G. Mansion, pers. obs.), further suggesting reticulate evolution in the group. A more detailed and collaborative study is currently on the way (A. Crowl et al., unpublished data).

#### * Clade cam15 (S: 21,71 Ma [8,94–26,74]/C: 2,36 Ma [0,83–12,80])

This strongly supported clade (BS 98, Fig. 5, Table 2) shows a largely unresolved clade with 16 Asian species unresolved or paraphyletic with respect to a mainly North-African clade. The latter was already depicted as a so-called “Azorina clade” by Borsch et al. [18], who overall pointed out the relationships between the Azorean endemic *Azorina*, the Cape Verdean endemics *C. bravensis* and *C. jacobaea*, and the E. African *C. edulis*. The current study gives a much more accurate picture of those relationships by defining two well-supported assemblages, sister to *Azorina*, that diversified during the Pleistocene (1.14 Ma [0.72–5.17], i.e. well after the emergence of the Azores archipelago (starting some 18 Ma ago [61]). The neoendemic genus *Azorina* has quickly diverged morphologically from *Campanula*, and is currently recognized by its shrubby aspect, its typical constricted flowers, and the presence of a flat nectar disk.

The first subclade (*C. balfourii*, *C. bravensis*, *C. jacobaea*, *C. keniensis*) (BS 82) depicts interesting biogeographical disjunction between a lineage from the Cape Verde Islands off western Africa, including the hexaploid species *C. bravensis* and *C. jacobea* (2 n = 54), and an eastern African lineage, with *C. balfourii* (Socotra) and *C. keniensis* (2 n = 54; Kenya). Disjunct distributions of plant groups between Macaronesia-NW Africa and E Africa-W Asia have been long recognized under the so-called “Rand Flora” [106], [107], and include e.g. the famous Canary Island *Dracaena draco* [108], *Phagnalon* [109], or *Canarina* (Campanulaceae; this study). This unexpected E-W relationships has been proposed as one possible explanation for the origin of the Cape Verde lineages by Leyens and Lobin [110], based on the chromosome number distinctiveness (2 n = 54).

The second subclade (*C. afra*, *C. mollis*, *C. edulis*, *C. filicaulis*, *C. kremeri*, *C. saxifragoides*) (BS 92; Fig. 5) contains six species mainly distributed in North Africa. The sister species *C. afra* and *C. kremeri* are morphologically very similar and have been treated as subspecies, or even synonyms [111], of *C. dichotoma* (not included here), with which they share the same chromosome number (2 n = 24) and similar geographical range (western North Africa, *C. afra* also described in southern Spain) [112]. In western Mediterranean Africa, the morphologically and karyologically polymorphic *C. filicaulis* [17], [113], with many potential dysploid and polyploid cytodemes described (2 n = 16, 24, 26, 48, 50, 52, 72), shows genetic affinities with *C. saxifragoides* (2 n = 14, 16). Finally, the phylogenetic position of the western Mediterranean *C. mollis* (2 n = 24, 26, 46, 48, 50, 52) and the eastern African *C. edulis* (2 n = 28, 56, 70) in this subclade remains unclear. Contandriopoulos et al. [113] interpreted the high polymorphism in chromosome numbers and morphotypes of both *C. filicaulis* and *C. mollis* to be the result of recent speciation events and incomplete lineage sorting, an assumption confirmed by the recent origin of the *Azorina*–*C. edulis* clade (stem node age = 1,30 Ma [0,98–4,64]; Fig. S3).

Overall, the African clade belongs to a larger assemblage including 16 additional species of primarily Asian origin. It is currently unclear whether these lineages are sister or paraphyletic with respect to each other. Most of the Asian species included here are perennial except for two annuals, namely *C. dimorphantha* (E Africa to Afghanistan and China) and *C. pallida* (Afghanistan to China). *Campanula dimorphantha* ( = *C. canescens* or *C. benthamii* [114]) is a widely distributed species, ranging from N Africa to Taiwan. Interestingly, this species produces both chasmogamous and cleistogamous flowers (the Chinese specimens being mostly cleistogamous), a reproductive strategy that could explain the current large range of this species. The other therophyte (*C. pallida*) also shows similar mating system and occurs from Afghanistan to Thailand. This species though is sometimes considered a perennial (*C. pallida* var. *tibetica*), and cleistogamous forms have also been described under a different species, *C. microcarpa* C. Y. Wu [115], overall adding some taxonomic confusion in the group. Among the remaining perennials, some form morphologically similar groups, including the Afghanistan-Pakistan endemics *C. leucantha*, *C. leucoclada*, and *C. polyclada*, with appendiculate calyces, or *C. cashmeriana*, *C. kermanica* and *C. khorasanica* sometimes treated as subspecies of *C. incanescens*. On the whole, the taxonomy of the Asian group is far from being resolved, most species being separated by inconspicuous characters. Furthermore, the recent time of divergence of the whole clade would suggest rapid episodes of diversification the polarity of which needs to be investigated.

#### * Clade Cam16 (S: 26,53 Ma [8,62–32,15]/C: 25,33 Ma [6,64–29,77])

This clade shows weak sister relationships (BS 57; Fig. 5, Table 2) between a lineage of two perennial species (*Campanula rumeliana* and *C. jacquinii*; BS 100), and an assemblage (BS 75) containing both annuals (11) and perennials (3). The strong affinitiy between *C. rumeliana* and *C. jacquinii* has already been suggested [116], but the absence of genetic relationships with the otherwise morphologically similar species (e.g. *C. asperuloides*, *C. buseri*, or *C. myrtifolia*) here included in Cam13, refutes their taxonomic inclusion in either *Diosphaera* or *Tracheliopsis*.

The second lineage (BS 75) shows further affinities between annual species of the respective subgenera *Sicyocodon* (*C. macrostyla*), *Megalocalyx* (*C. propinqua*, *C. strigosa*, *C. hierosolymitana*, *C*. *camptoclada*, *C. cecilii*, and *C. reuteriana*), *Roucela* (*C. scutellata*), and the perennials *C. damascena*, *C. mardinensis*, and *C. lourica*. Although most species of the subgenus *Megalocalyx* are very polymorphic and difficult to separate morphologically [111], they appear to have evolved in two lineages that originated in the early Miocene (24,67 Ma [6], [11]–[28], [75]). On the one hand, most species of *Megalocalyx* are sister to *C. macrostyla*, a singular species with a combination of characters not found in any other extant species of *Campanula*, subsequently classified in the monotypic subgenus *Sicyocodon* [15], [111]. Albeit partially unresolved, this clade depicts relationships between annuals currently occurring in the Near-East region, from Turkey to Egypt. On the other hand, an annual *C. scutellata*–*C. stellaris* lineage is sister to the Iranian perennial *C. lourica*. Both *C. scutellata* and *C. stellaris* differ by the presence (*C. scutellata*) vs. absence (*C. stellaris*) of calyx appendages, but exhibit particular stellate and accrescent calyces after fructification. *Campanula scutellata* has long been considered a particular species within subgenus *Roucela*, and must be clearly excluded from it. As mentioned for the annual species-rich clade Cam14, the possibility of reticulate evolution exists in the current clade, whose natural history inference would necessitate increasing taxonomic and geographic sampling, and more sensitive molecular markers.

#### * Clade cam17 (S: 28,53 Ma [8,62–32,15]/C: 4,57 Ma [2,65–10,71])

This huge and well-supported clade (BS 73; Fig. 6, Table 2), with some 195 species/subspecies of *Campanula* s.l., including the genus’ type species (*Campanula latifolia* L.), remains globally unresolved. In most cases, individuals from the same species were grouped as sisters, but there were also cases with high diversity such as *C. sibirica*, *C. barbata*, *C. spatulata*, or *C. lingulata*, where this study can guide future phylogeographic/speciation studies.

Several technical and biological explanations have been proposed for the phylogenetic inference of non-bifurcating trees, with soft or hard polytomies, including gene choice, rapid diversification of lineages, or reticulate evolution [117], [118]. The *petD* region has been used to resolve successfully phylogenetic patterns at different taxonomic levels [30], [33], [119]. Overall, the polytomy of the Cam17 lineage has also been exemplified by the *trnLF* [13] and *rpl*16 (unpublished data) regions. While the combined use of different markers poorly resolved such lineage [21], [67], it has to be awaited how the addition of information from genomic regions with high level of hierarchical phylogenetic signal will improve the situation. Organellar and nuclear genomic compartments should thereby be analyzed independently to test for possible incongruence.

At the organismal level, the inferred timing of lineage diversification, combined with the accumulation of taxa in particular regions of the eastern Mediterranean and Middle-East (most accessions in Cam17 come from Greece, Turkey, and the Caucasus), would support recent patterns of hyper-diversification. This hypothesis needs to be tested with comprehensive biogeographic methods and estimations of lineage through time accumulation for the entire clade. Finally, the occurrence of particular events known to disrupt phylogenetic bifurcation, such as incomplete sorting of lineages, or hybridization and introgression associated or not with genome duplication, cannot be ruled-out in the present case. Overall, we feel that a combination of the aforementioned factors (low phylogenetic information and noise) might provide the most likely explanation for the current comb-like structure of clade Cam17.

## Conclusions and Perspectives

In this study, we used comprehensive taxon-sampling including as many species as possible in order to provide a phylogenetic framework for *Campanula* and allies. The use of a group II intron sequence [120] allowed the efficient generation of a well-supported tree. There are several arguments suggesting that our approach of a mass sampling strategy should be the first step in any evolutionary study of highly-diversified clades.

Mass taxon-sampling was the only effective way to infer a satisfactory phylogenetic hypothesis for *Campanula* s.lat., recovering 17 well-supported clades as potential robust units for more detailed evolutionary studies. Even the dramatic accumulation of nearly identical sequences in some clades, otherwise containing morphologically well-differentiated species (e.g. Cam12 and Cam17), can be viewed as an indication of some underlying evolutionary processes including reticulation or shifts in species diversification rates (e.g. phenotypic evolution can be faster than the accumulation of nucleotide changes in the marker region). In this respect, mass sampling considerably advanced our knowledge on *Campanula* and allies.

Our results underscore the possible limits of a sampling scheme when guided by a pre-cladistic classification system. Comparison of data sets D088 and D680 showed that classification-guided sampling inferred biased topologies with either missing or non-satisfactorily circumscribed clades (e.g. most morpho-types in fact fall into the large and unresolved Cam17 clade). In this context, it seems that the inclusion of as many species as possible is the best approach to reconstruct realistic tree symmetry (tree shape), and thus constitutes a mandatory basis to understand morphological evolution and infer biogeographical patterns in highly plastic groups.

We determined that a phylogeny-guided taxon sampling (D101 vs. D680) inferred significantly different age estimates (P = 0.02) and BS values (P = 0.009) when compared to the D680 estimates. Therefore, despite the potential accumulation of homoplastic signal in some clades (e.g. Cam12 and Cam17), dense taxon-sampling (that eventually break long branches) overall led to better supported trees.

In a more intrinsic and theoretical context, the effects of taxon sampling on the accuracy of phylogeny inference and the estimation of various evolutionary parameters are still intensely discussed [23], [27], [121]. While case and simulation studies usually ask whether it is better to sample characters versus taxa to avoid long branch attraction and improve node support [23], [27], [121], [122], [123], they lack testing the effects of selective sampling on tree resolution and support with large sets of real data, and thus largely overlook the issue of correct tree shape. Our approach, testing nearly full taxon sampling in a species-rich clade versus selective strategies, highly overcame those issues.

Finally, the generation of large intron sequence data sets is promising to allow an efficient integration of evolutionary analysis and species diversity assessment that goes beyond DNA barcoding. Recent insights from a multiple sequence data set in epiphytic Cactaceae indicate that the most variable plastid spacer sequences may not contain the highest level of hierarchical phylogenetic signal [29], while plastid introns hold promise for both. Our study provides the largest so far constructed multiple sequence alignment for a group II intron in angiosperms. Future work can then test relative phylogenetic utility (and improve phylogenetic trees) and species identification potential of further genomic regions to be added using the same samples. Due to the presence of the *petD* group II intron as well as many other introns [30] as orthologs in all flowering plant and most land plants the mass sampling approach can be universally applied.

## Supporting Information

Figure S1 Bayesian majority-rule phylogram of *Campanula* and relatives (D680). Posterior probability values are indicated below branches. Gray boxes indicate the respective outgroup sister clades; blue boxes refer to “Cam” clades containing at least one accession of *Campanula* (Cam01 to Cam17; see text). A blue dot indicates the crown node of *Campanula* s.lat. LOBE = Lobelioideae; CYPHI: Cyphioideae; CA-CYA: Campanuloideae-Cyanantheae; CA-WAH: Campanuloideae-Wahlenbergieae.
(PDF)

Figure S2 Best Maximum Likelihood phylogram of *Campanula* and relatives (D680). Bootstrap support for clades are indicated below branches. Gray boxes indicate the respective outgroup sister clades; blue boxes refer to “Cam” clades containing at least one accession of *Campanula* (Cam01 to Cam17; see text). A blue dot indicates the crown node of *Campanula* s.lat. LOBE = Lobelioideae; CYPHI: Cyphioideae; CA-CYA: Campanuloideae-Cyanantheae; CA-WAH: Campanuloideae-Wahlenbergieae.
(PDF)

Figure S3 Chronogram of *Campanula* and relatives (D680) inferred from the penalized-likelihood method implemented in r8s, and dated using one fossil constraint (yellow spiral). The yellow box refers to the time span between the stem and crown node of *Campanula* s.lat. Gray boxes indicate the respective outgroup sister clades; blue boxes refer to “Cam” clades containing at least one accession of *Campanula* (Cam01 to Cam17; see text). Ma = Mega Annuum or Million years; LOBE = Lobelioideae; CYPHI: Cyphioideae; CA-CYA: Campanuloideae-Cyanantheae; CA-WAH: Campanuloideae-Wahlenbergieae.
(PDF)

Figure S4 Maximum Parsimony Strict consensus tree of *Campanula* and relatives (D088). Values below branches indicate bootstrap support for sustained clade. Gray boxes indicate the respective outgroup sister clades; blue boxes refer to “Cam” clades containing at least one accession of *Campanula* (Cam01 to Cam17; see text). A blue dot indicates the crown node of *Campanula* s.lat. LOBE = Lobelioideae; CYPHI: Cyphioideae; CA-CYA: Campanuloideae-Cyanantheae; CA-WAH: Campanuloideae-Wahlenbergieae.
(PDF)

Figure S5 Bayesian majority-rule phylogram of *Campanula* and relatives (D088). Posterior probability values are indicated below branches. Gray boxes indicate the respective outgroup sister clades; blue boxes refer to “Cam” clades containing at least one accession of *Campanula* (Cam01 to Cam17; see text). A blue dot indicates the crown node of *Campanula* s.lat. LOBE = Lobelioideae; CYPHI: Cyphioideae; CA-CYA: Campanuloideae-Cyanantheae; CA-WAH: Campanuloideae-Wahlenbergieae.
(PDF)

Figure S6 Best Maximum Likelihood phylogram of *Campanula* and relatives (D088). Bootstrap support for clades are indicated below branches. Gray boxes indicate the respective outgroup sister clades; blue boxes refer to “Cam” clades containing at least one accession of *Campanula* (Cam01 to Cam17; see text). A blue dot indicates the crown node of *Campanula* s.lat. LOBE = Lobelioideae; CYPHI: Cyphioideae; CA-CYA: Campanuloideae-Cyanantheae; CA-WAH: Campanuloideae-Wahlenbergieae.
(PDF)

Figure S7 Chronogram of *Campanula* and relatives (D088) inferred from the penalized-likelihood method implemented in r8s, and dated using one fossil constraint (yellow spiral). The yellow box refers to the time span between the stem and crown node of *Campanula* s.lat. Gray boxes indicate the respective outgroup sister clades; blue boxes refer to “Cam” clades containing at least one accession of *Campanula* (Cam01 to Cam17; see text). Ma = Mega Annuum or Million years; LOBE = Lobelioideae; CYPHI: Cyphioideae; CA-CYA: Campanuloideae-Cyanantheae; CA-WAH: Campanuloideae-Wahlenbergieae.
(PDF)

Figure S8 Maximum Parsimony Strict consensus tree of *Campanula* and relatives (D101). Values below branches indicate bootstrap support for sustained clade. Gray boxes indicate the respective outgroup sister clades; blue boxes refer to “Cam” clades containing at least one accession of *Campanula* (Cam01 to Cam17; see text). A blue dot indicates the crown node of *Campanula* s.lat. LOBE = Lobelioideae; CYPHI: Cyphioideae; CA-CYA: Campanuloideae-Cyanantheae; CA-WAH: Campanuloideae-Wahlenbergieae.
(PDF)

Figure S9 Bayesian majority-rule phylogram of *Campanula* and relatives (D101). Posterior probability values are indicated below branches. Gray boxes indicate the respective outgroup sister clades; blue boxes refer to “Cam” clades containing at least one accession of *Campanula* (Cam01 to Cam17; see text). A blue dot indicates the crown node of *Campanula* s.lat. LOBE = Lobelioideae; CYPHI: Cyphioideae; CA-CYA: Campanuloideae-Cyanantheae; CA-WAH: Campanuloideae-Wahlenbergieae.
(PDF)

Figure S10 Best Maximum Likelihood phylogram of *Campanula* and relatives (D101). Bootstrap support for clades are indicated below branches. Gray boxes indicate the respective outgroup sister clades; blue boxes refer to “Cam” clades containing at least one accession of *Campanula* (Cam01 to Cam17; see text). A blue dot indicates the crown node of *Campanula* s.lat. LOBE = Lobelioideae; CYPHI: Cyphioideae; CA-CYA: Campanuloideae-Cyanantheae; CA-WAH: Campanuloideae-Wahlenbergieae.
(PDF)

Figure S11 Chronogram of *Campanula* and relatives (D101) inferred from the penalized-likelihood method implemented in r8s, and dated using one fossil constraint (yellow spiral). The yellow box refers to the time span between the stem and crown node of *Campanula* s.lat. Gray boxes indicate the respective outgroup sister clades; blue boxes refer to “Cam” clades containing at least one accession of *Campanula* (Cam01 to Cam17; see text). Ma = Mega Annuum or Million years; LOBE = Lobelioideae; CYPHI: Cyphioideae; CA-CYA: Campanuloideae-Cyanantheae; CA-WAH: Campanuloideae-Wahlenbergieae.
(PDF)

Table S1 List of species, including voucher information and Genbank accessions, used in phylogenetic analyses. An asterisk indicates molecular sequence directly retrieved from Genbank.
(PDF)

Table S2 Overview of a potential infra-genetic classification of *Campanula* L. Type species used for the classification-guided sampling are indicated in bold green.
(PDF)
