# Supplementary figures and images for: How to Handle Speciose Clades? Mass Taxon-Sampling as a Strategy towards Illuminating the Natural History of Campanula (Campanuloideae)
Source: PLoS One. 2012 Nov 28;7(11):e50076. doi: 10.1371/journal.pone.0050076 (PMC3509159; doi:10.1371/journal.pone.0050076)

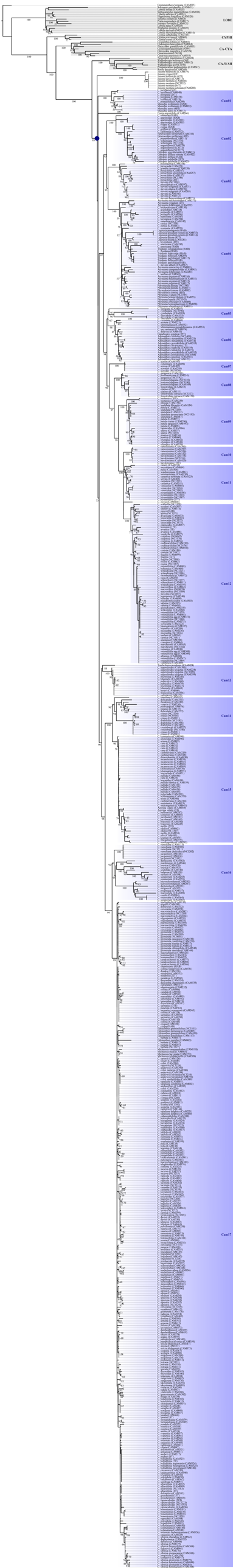

Supplement: Figure S2 — Best Maximum Likelihood phylogram of Campanula and relatives (D680). Bootstrap support for clades are indicated below branches. Gray boxes indicate the respective outgroup sister clades; blue boxes refer to “Cam” clades containing at least one accession of Campanula (Cam01 to Cam17; see text). A blue dot indicates the crown node of Campanula s.lat. LOBE = Lobelioideae; CYPHI: Cyphioideae; CA-CYA: Campanuloideae-Cyanantheae; CA-WAH: Campanuloideae-Wahlenbergieae. (PDF) [file pone.0050076.s002.pdf]

163

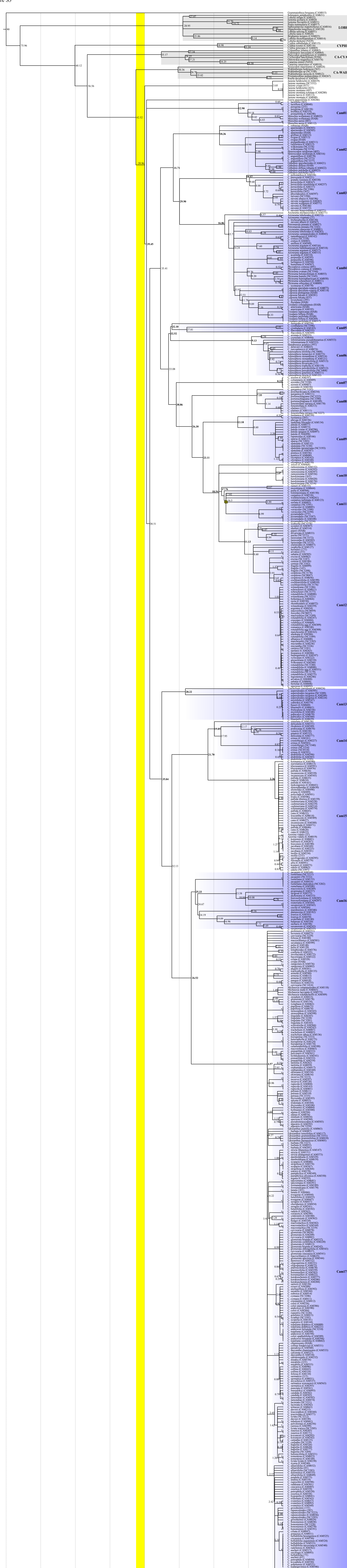

Supplement: Figure S3 — Chronogram of Campanula and relatives (D680) inferred from the penalized-likelihood method implemented in r8s, and dated using one fossil constraint (yellow spiral). The yellow box refers to the time span between the stem and crown node of Campanula s.lat. Gray boxes indicate the respective outgroup sister clades; blue boxes refer to “Cam” clades containing at least one accession of Campanula (Cam01 to Cam17; see text). Ma = Mega Annuum or Million years; LOBE = Lobelioideae; CYPHI: Cyphioideae; CA-CYA: Campanuloideae-Cyanantheae; CA-WAH: Campanuloideae-Wahlenbergieae. (PDF) [file pone.0050076.s003.pdf]

Figure S4

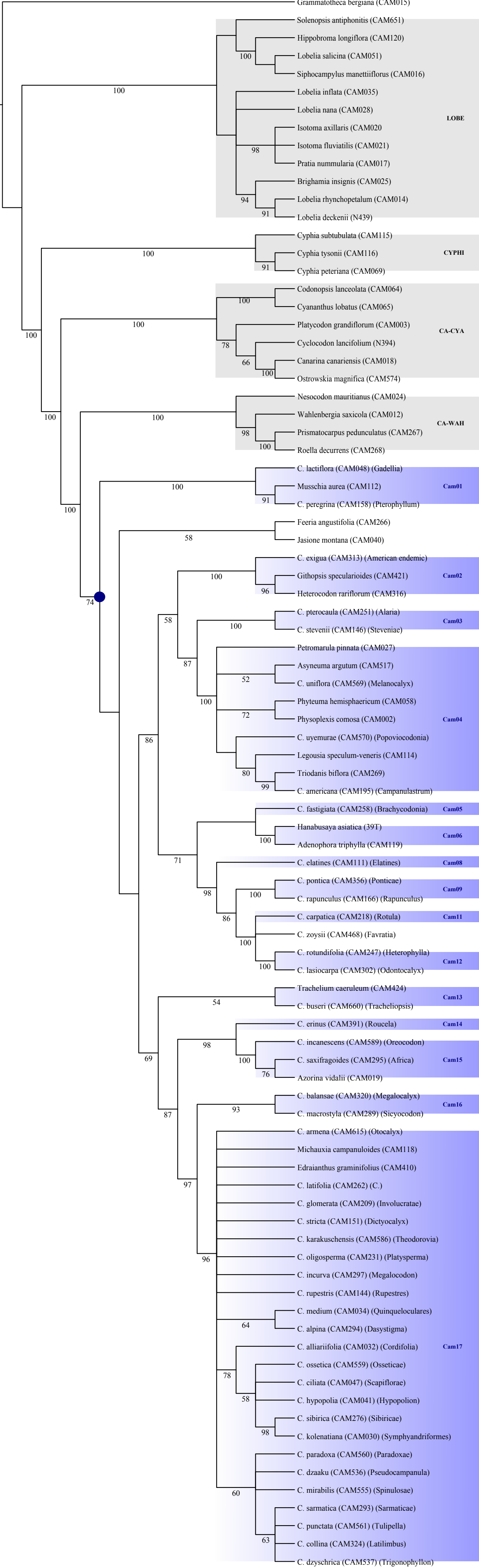

Supplement: Figure S4 — Maximum Parsimony Strict consensus tree of Campanula and relatives (D088). Values below branches indicate bootstrap support for sustained clade. Gray boxes indicate the respective outgroup sister clades; blue boxes refer to “Cam” clades containing at least one accession of Campanula (Cam01 to Cam17; see text). A blue dot indicates the crown node of Campanula s.lat. LOBE = Lobelioideae; CYPHI: Cyphioideae; CA-CYA: Campanuloideae-Cyanantheae; CA-WAH: Campanuloideae-Wahlenbergieae. (PDF) [file pone.0050076.s004.pdf]

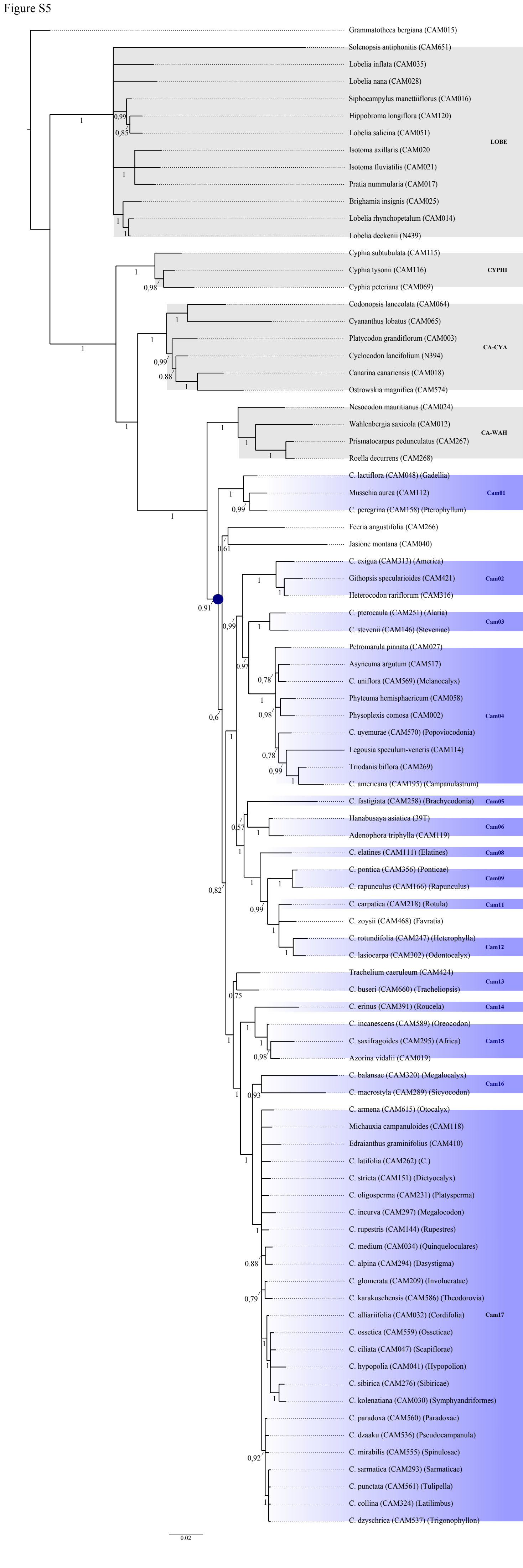

Supplement: Figure S5 — Bayesian majority-rule phylogram of Campanula and relatives (D088). Posterior probability values are indicated below branches. Gray boxes indicate the respective outgroup sister clades; blue boxes refer to “Cam” clades containing at least one accession of Campanula (Cam01 to Cam17; see text). A blue dot indicates the crown node of Campanula s.lat. LOBE = Lobelioideae; CYPHI: Cyphioideae; CA-CYA: Campanuloideae-Cyanantheae; CA-WAH: Campanuloideae-Wahlenbergieae. (PDF) [file pone.0050076.s005.pdf]

Figure S6

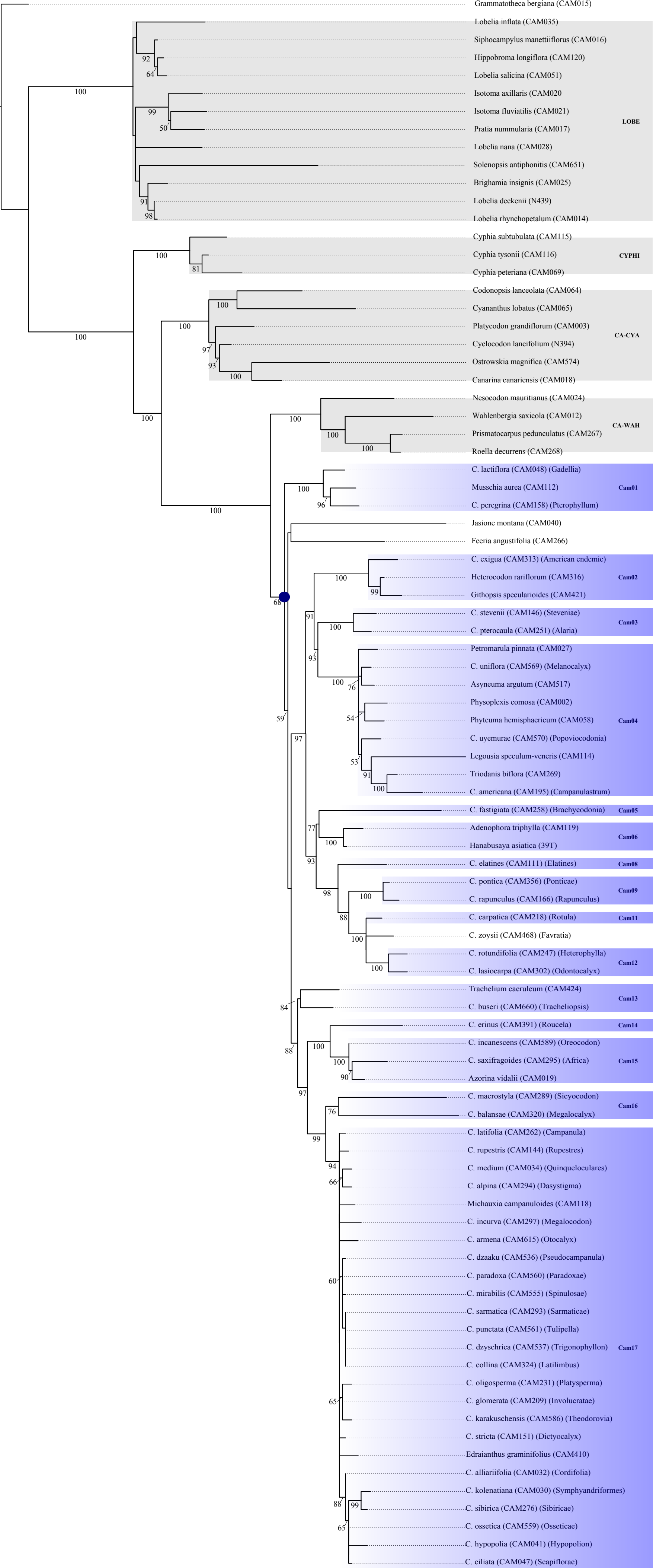

0.02

Supplement: Figure S6 — Best Maximum Likelihood phylogram of Campanula and relatives (D088). Bootstrap support for clades are indicated below branches. Gray boxes indicate the respective outgroup sister clades; blue boxes refer to “Cam” clades containing at least one accession of Campanula (Cam01 to Cam17; see text). A blue dot indicates the crown node of Campanula s.lat. LOBE = Lobelioideae; CYPHI: Cyphioideae; CA-CYA: Campanuloideae-Cyanantheae; CA-WAH: Campanuloideae-Wahlenbergieae. (PDF) [file pone.0050076.s006.pdf]

Figure S7

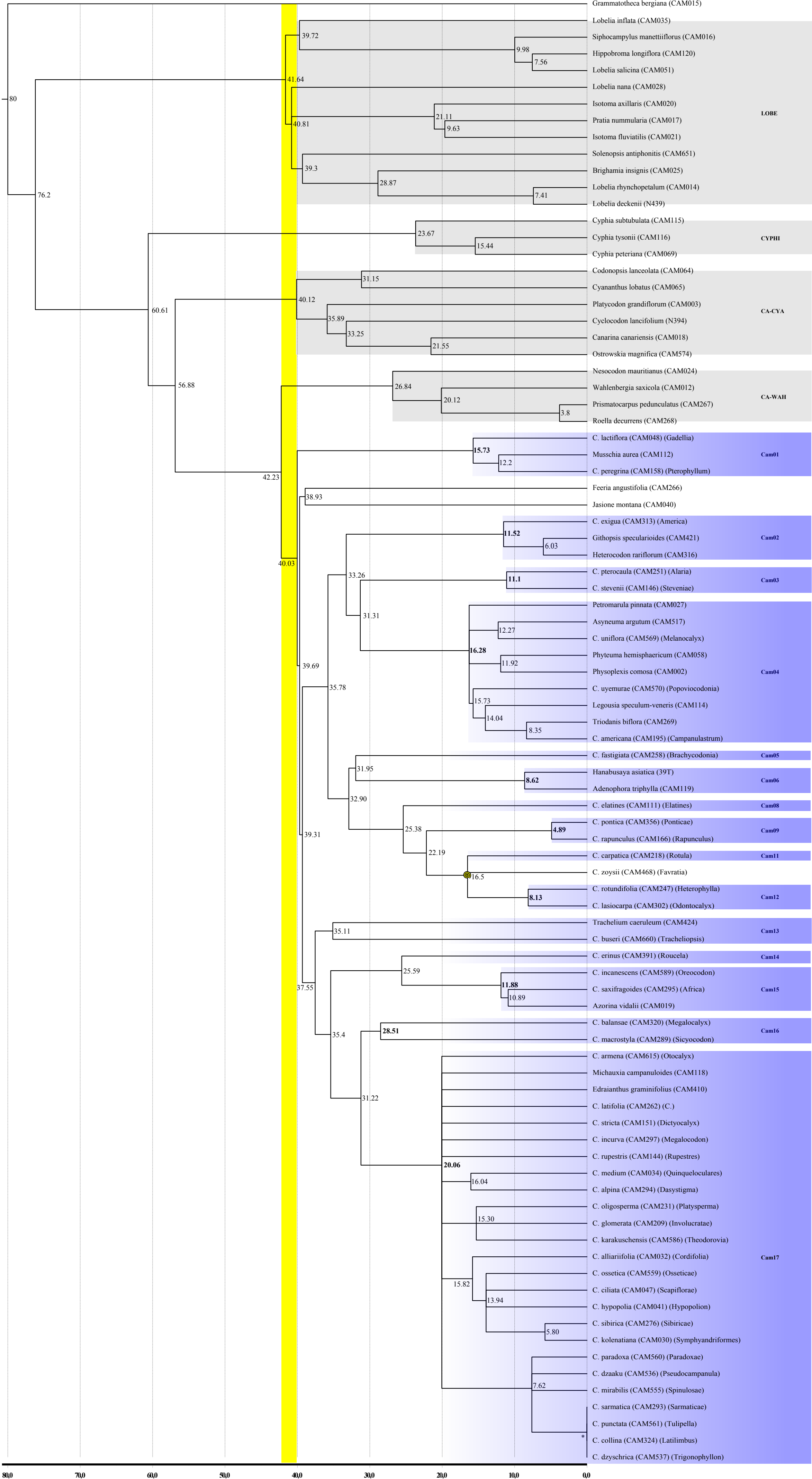

Supplement: Figure S7 — Chronogram of Campanula and relatives (D088) inferred from the penalized-likelihood method implemented in r8s, and dated using one fossil constraint (yellow spiral). The yellow box refers to the time span between the stem and crown node of Campanula s.lat. Gray boxes indicate the respective outgroup sister clades; blue boxes refer to “Cam” clades containing at least one accession of Campanula (Cam01 to Cam17; see text). Ma = Mega Annuum or Million years; LOBE = Lobelioideae; CYPHI: Cyphioideae; CA-CYA: Campanuloideae-Cyanantheae; CA-WAH: Campanuloideae-Wahlenbergieae. (PDF) [file pone.0050076.s007.pdf]

Figure S8

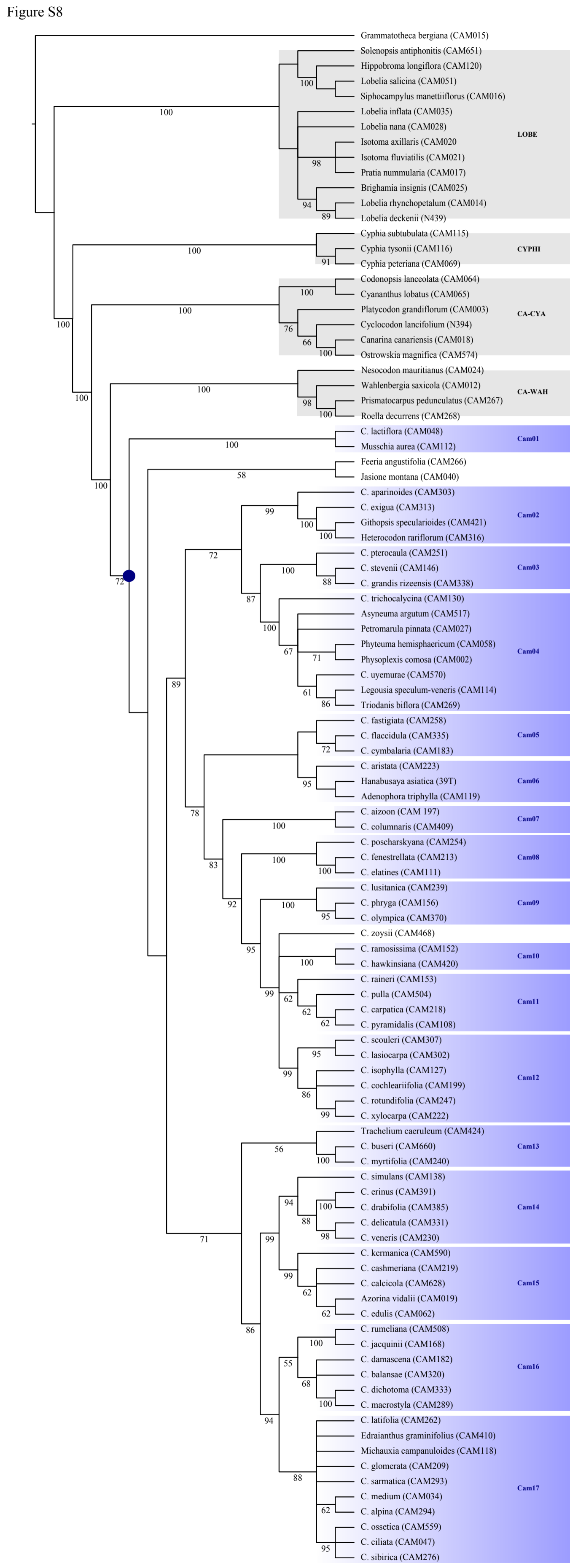

Supplement: Figure S8 — Maximum Parsimony Strict consensus tree of Campanula and relatives (D101). Values below branches indicate bootstrap support for sustained clade. Gray boxes indicate the respective outgroup sister clades; blue boxes refer to “Cam” clades containing at least one accession of Campanula (Cam01 to Cam17; see text). A blue dot indicates the crown node of Campanula s.lat. LOBE = Lobelioideae; CYPHI: Cyphioideae; CA-CYA: Campanuloideae-Cyanantheae; CA-WAH: Campanuloideae-Wahlenbergieae. (PDF) [file pone.0050076.s008.pdf]

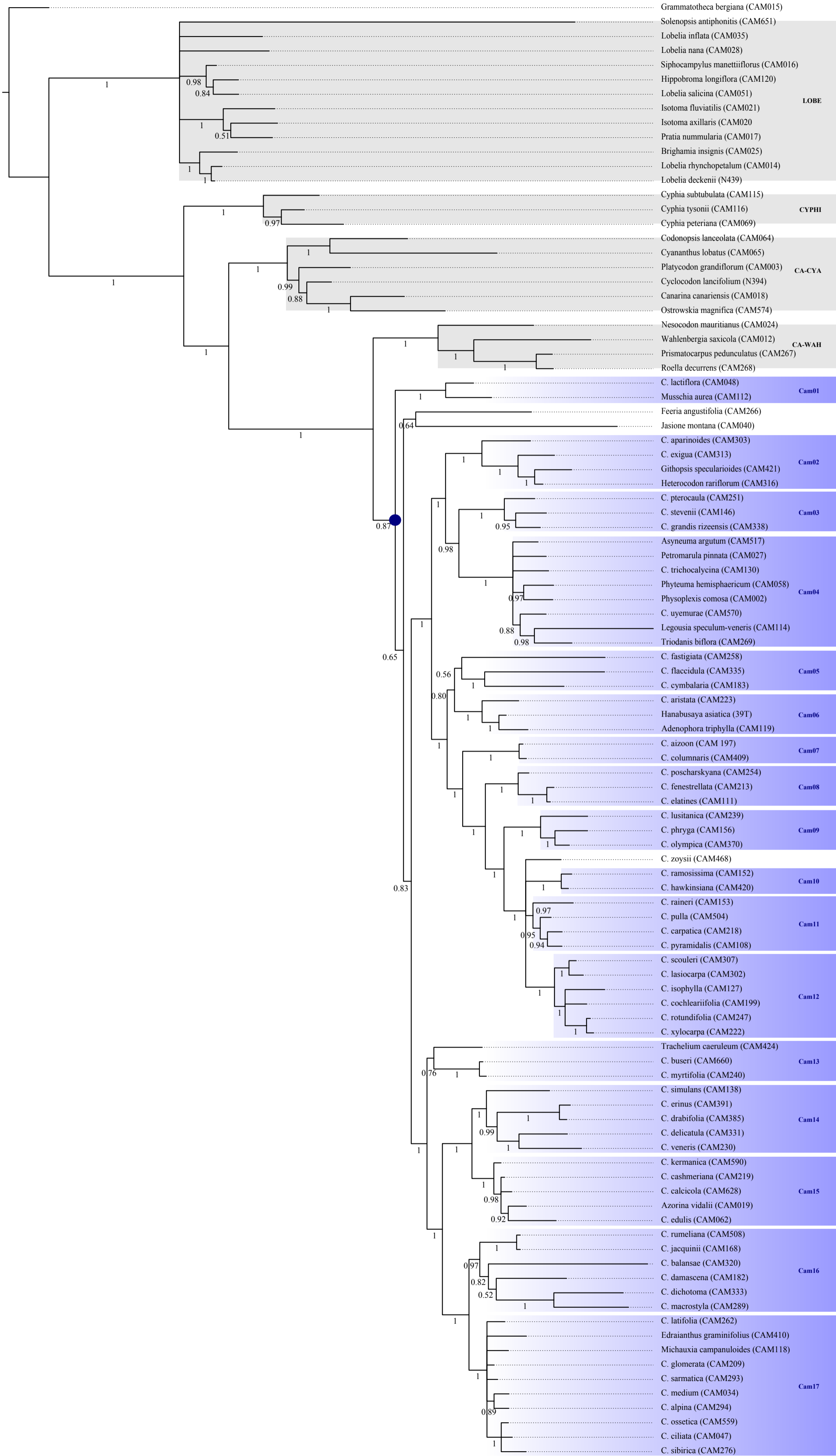

Supplement: Figure S9 — Bayesian majority-rule phylogram of Campanula and relatives (D101). Posterior probability values are indicated below branches. Gray boxes indicate the respective outgroup sister clades; blue boxes refer to “Cam” clades containing at least one accession of Campanula (Cam01 to Cam17; see text). A blue dot indicates the crown node of Campanula s.lat. LOBE = Lobelioideae; CYPHI: Cyphioideae; CA-CYA: Campanuloideae-Cyanantheae; CA-WAH: Campanuloideae-Wahlenbergieae. (PDF) [file pone.0050076.s009.pdf]

Figure S10

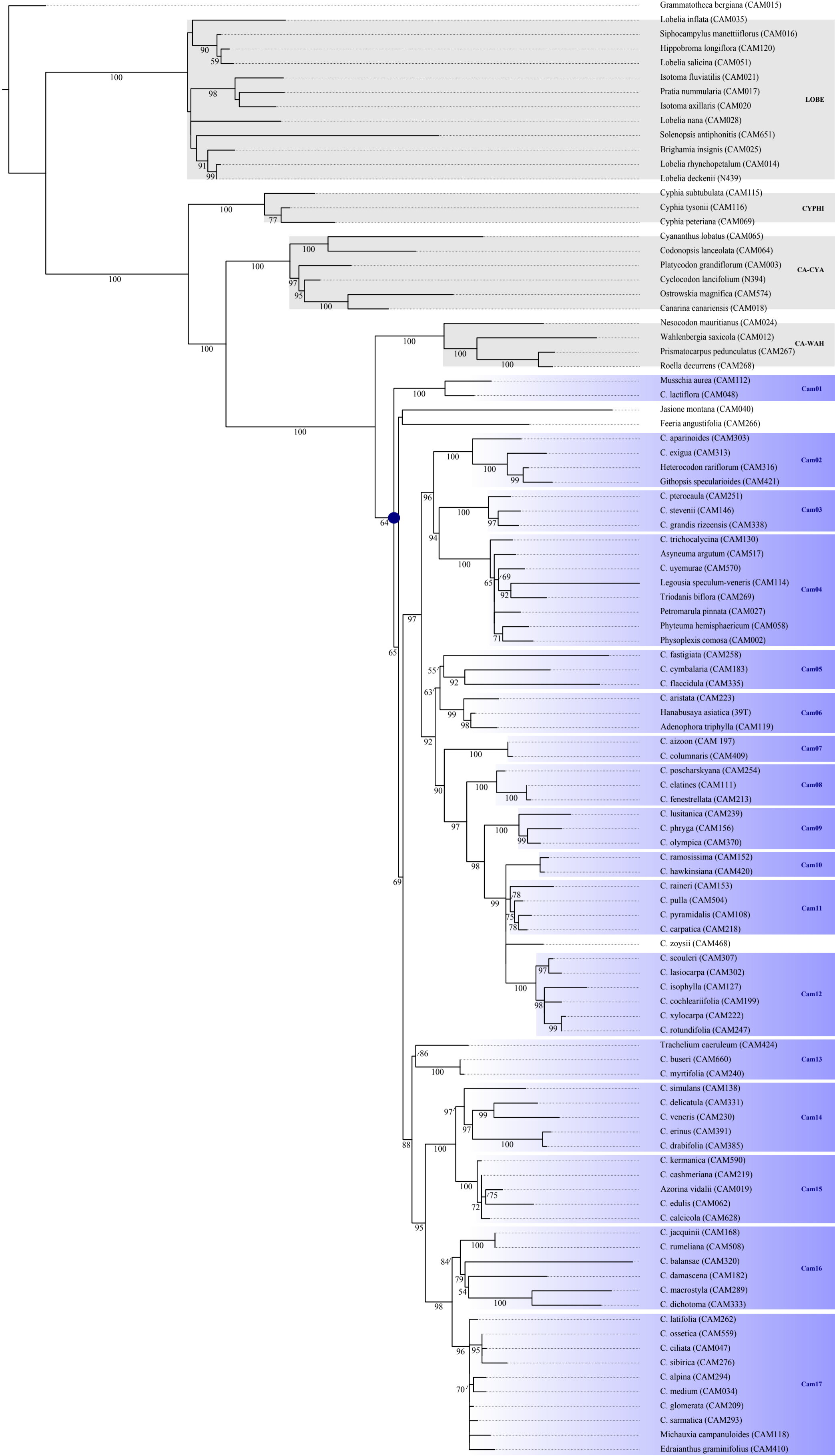

0.02

Supplement: Figure S10 — Best Maximum Likelihood phylogram of Campanula and relatives (D101). Bootstrap support for clades are indicated below branches. Gray boxes indicate the respective outgroup sister clades; blue boxes refer to “Cam” clades containing at least one accession of Campanula (Cam01 to Cam17; see text). A blue dot indicates the crown node of Campanula s.lat. LOBE = Lobelioideae; CYPHI: Cyphioideae; CA-CYA: Campanuloideae-Cyanantheae; CA-WAH: Campanuloideae-Wahlenbergieae. (PDF) [file pone.0050076.s010.pdf]

Figure S11

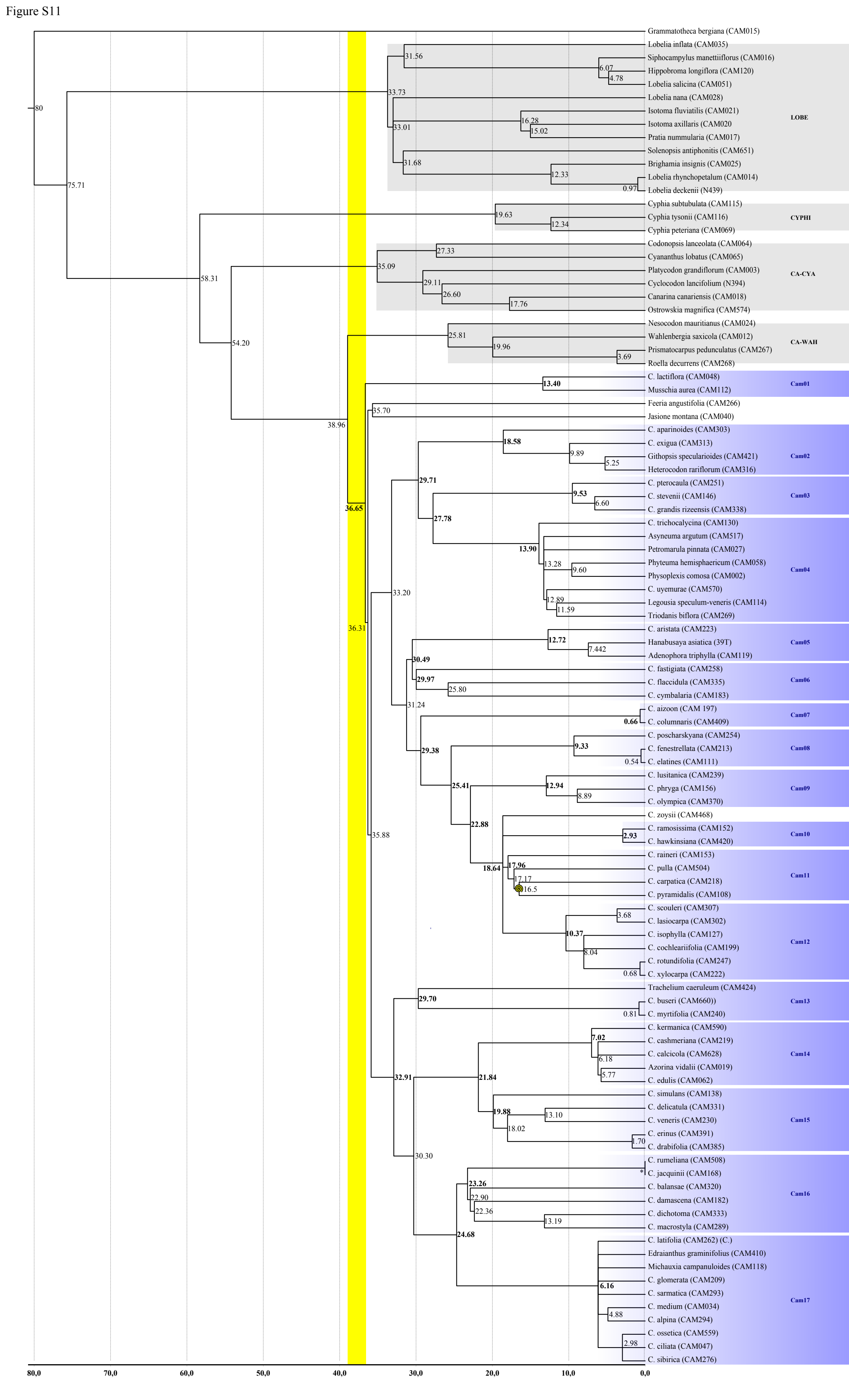

Supplement: Figure S11 — Chronogram of Campanula and relatives (D101) inferred from the penalized-likelihood method implemented in r8s, and dated using one fossil constraint (yellow spiral). The yellow box refers to the time span between the stem and crown node of Campanula s.lat. Gray boxes indicate the respective outgroup sister clades; blue boxes refer to “Cam” clades containing at least one accession of Campanula (Cam01 to Cam17; see text). Ma = Mega Annuum or Million years; LOBE = Lobelioideae; CYPHI: Cyphioideae; CA-CYA: Campanuloideae-Cyanantheae; CA-WAH: Campanuloideae-Wahlenbergieae. (PDF) [file pone.0050076.s011.pdf]
