# Supplementary material for: How to Handle Speciose Clades? Mass Taxon-Sampling as a Strategy towards Illuminating the Natural History of Campanula (Campanuloideae)
Source: PLoS One. 2012 Nov 28;7(11):e50076. doi: 10.1371/journal.pone.0050076 (PMC3509159; doi:10.1371/journal.pone.0050076)
Supplement: Table S1 — List of species, including voucher information and Genbank accessions, used in phylogenetic analyses. An asterisk indicates molecular sequence directly retrieved from Genbank. (PDF) [file pone.0050076.s012.pdf]

Table S1 – List of species, including voucher information and Genbank accessions, used in phylogenetic analyses. An asterisk designs molecular sequence directly retrieved from Genbank. BG: Botanical Garden

Genus, Species	DNA Number and Voucher	GenBank Number
ADENOPHORA		
Adenophora divaricata Franch. & Sav.	Eddie 96086, 1T (EGHB)	JX914669
Adenophora gmelinii (Biehler) Fisch.	Russia, Rupsova 8648.2, CAM511 (LE)	JX914983
Adenophora lamarkii Fisch.	Russia, Altay Republic, Ulagansiy Rayon, Aktash, L. Martins 2393, CAM273 (B)	JX914802
Adenophora liliifolia (L.) A.DC.	Scortegagna 5465, NC3409 (MRSN)	JX915239
Adenophora pereskiifolia Fisch.	Russia, Volkova s.n., CAM512 (LE)	JX914984
Adenophora pereskiifolia Fisch.	Russia, Sakhalin, Pobedinova et al sn, CAM513 (LE)	JX914985
Adenophora pereskiifolia Fisch.	Furuse 6999, NC3408 (MRSN)	JX915238
Adenophora stenanthina (Ledeb.) Kitag.	Without locality, CAM121, BG Bonn 26084 (BONN)	JX914673
Adenophora stenanthina (Ledeb.) Kitag.	Russia, Near Riga, Rukzemin sn, CAM514 (LE)	JX914986
Adenophora stricta Miq.	Without locality, CAM122, BG Bonn 27769 (BONN)	JX914674
Adenophora triphylla (Thunb.) A.DC.	GenBank	FN396976*
ASYNEUMA		
Asyneuma argutum subsp. *baldshuanicum* (Fedtsch.) Damboldt	Russian label, CAM516 (LE)	JX914988
Asyneuma argutum subsp. *baldshuanicum* (Fedtsch.) Damboldt	Russian label, CAM518 (LE)	JX914990
Asyneuma argutum (Regel) Bornm.	Russian label, CAM515 (LE)	JX914987
Asyneuma argutum (Regel) Bornm.	Russian label, CAM517 (LE)	JX914989
Asyneuma campanuloides (M. Bieb. ex Sims) Bornm	GenBank	FN396978*
Asyneuma canescens Griseb. & Schenk	GenBank	FN396979*
Asyneuma lobelioides (Willd.) Hand.-Mazz.	Turkey, C3 Antalya, Saklilent, Döring et al. 421, CAM272 (B)	JX914801
Asyneuma lobelioides (Willd.) Hand.-Mazz.	Iran, W Azarbaijan, Uromieh, S. Zarre et al. 35270, CAM283 (B)	JX914809
Asyneuma michauxioides (Boiss.) Damboldt	Turkey, C2 Denizli, Honaz Dagi, Eren & Parolly 7538, CAM271 (B)	JX914800
Asyneuma virgatum (Labill.) Bornm.	Berlin-Dahlem 0104, 2T (OS)	JX914670
AZORINA		
Azorina vidalii Ferr.	GenBank	FN396980*
BRIGHAMIA		
Brighamia insignis A.Gray	GenBank	FN396981*
CAMPANULA		
Campanula acutiloba Vatke	Turkey, C9 Hakkari, Yüksekova, Daglica-Yesiltas arasi, A. Dönmez 11341, CAM318 (HUB)	JX914841
Campanula afra Cav.	Morocco, Monts des Beni-Snassen, R. Vogt 11008 & C. Oberprieler 5456, CAM491 (B)	JX914970
Campanula aizoides Zaffran ex Greuter	Greece, Crete, Nomos Chania, Sfakia, E. Bergmeier & U. Matthäs 3415, CAM216 (B)	JX914753
Campanula aizoides Zaffran ex Greuter	Avramakis s.n., NC3330 (NHMC)	JX915202
Campanula aizoon Boiss. & Spruner	Greece, Nomos Phocis, Jiona, E. Eisenblätter & E. Willing 47.574, CAM197 (B)	JX914736
Campanula aizoon Boiss. & Spruner	Greece, Sterea Ellas, Nomos Fokidos, Mt Giona, T. Raus et al 32143-7, CAM407 (B)	JX914926
Campanula akguelli Altan	Iran, Y. Altan 4671, CAM581 (TARI)	JX915050
Campanula alaskana (A. Gray) Wight	Canada, British-Columbia, Queen Charlotte Islands, D. Veale s.n., CAM306 (UBC)	JX914830
Campanula alata Desf. [Campanula primulifolia Brot.]	Portugal Algarve, Serra de Monchique, CAM298 (BG Berlin)	JX914823
Campanula albanica Witasek	Greece, Sterea Ellas, Nomos Fokidos, Mt Vardousia, T. Raus et al 32150-1, CAM408 (B)	JX914927
Campanula alliariifolia Willd.	Cosner s. n., 4T (OS)	JX915112
Campanula alliariifolia Willd.	GenBank	FN396987*
Campanula alliariifolia Willd.	GenBank	FN397022*
Campanula alliariifolia Willd.	NC3383	JX915225
Campanula alpestris All.	France, Haute-Alpes, Galibier, CAM292 (BG Berlin)	JX914817
Campanula alpestris All.	Abba 1446, NC3329 (MRSN)	JX915201
Campanula alpina Jacq.	Austria, Steiermark, Wölzer Tauern, CAM294, (BG Berlin)	JX914819
Campanula alsinoides Hook. f.	Pakistan, Karakoram, Nagar village area, O. Polunin 6416, CAM490 (B)	JX914969
Campanula americana L.	USA, Missouri, Jefferson Co, S of Hillsborro, J. Stone & J. Miller 1325, CAM195 (B)	JX914734
Campanula americana L.	Ted Bradley 27643, HAB (GMU)	JX915133
Campanula anchusiflora Salisb. ex Sm.	Greece, Nomos Piraeus, Poros Island, G. Mansion 10027, CAM392 (B)	JX914912
Campanula andina Rupr.	Georgia, Zeylam, K. Kimeridze 460-80, CAM519 (LE)	JX914991
Campanula andrewsii A. DC.	Greece, Nomos Corinthia, Katakali, R. Eisenblätter & E. Willing 41.581, CAM194 (B)	JX914733
Campanula andrewsii A. DC.	Greece, Nomos Argolis, Neo Epidavros, G. Mansion 10022, CAM390 (B)	JX914910
Campanula andrewsii subsp. *hirsutula* Phitos	Greece, Nomos Laconia, Monenvasia, G. Mansion 10049, CAM394 (B)	JX914914
Campanula andrewsii subsp. *hirsutula* Phitos	NC3210	JX915183
Campanula angustiflora Eastw.	USA, California, Mt Tamalpais, J. Howell s.n., CAM578 (B)	JX915047
Campanula angustiflora Eastw.	NC3373	JX915219
Campanula angustiflora Eastw.	NC3377	JX915223
Campanula aparinoides Pursh	Canada, Manitoba, Riding Mountain National Park, W. Wojtas 693, CAM303 (UBC)	JX914828
Campanula aparinoides Pursh	Canada, Ontario, Thunder Bay, C. Garton 22898, CAM305 (UBC)	JX914829
Campanula aparinoides Pursh	Gary Fleming 5739, HAB (GMU)	JX915134
Campanula ardonensis Rupr.	Russia, South Caucasus, Arga, Nehmiski sn, CAM521 (LE)	JX914993
Campanula argaea Boiss. & Balansa	Turkey, C3 Antalya, Cubuk Geçidic, R. Ulrich s.n., CAM193 (B)	JX914732
Campanula argentea Lam.	Turkey, Gümüşhane, Gökdere Köyü, N. & O. İkinci sn, CAM434 (AIBU; B)	JX914944
Campanula ariana Podlech	Afghanistan, Karwan, H. Neubauer 3060, CAM489 (B)	JX914968
Campanula aristata Wall.	China, Qinghai, Menyuan, H. Kürschner & M. Sonnentag 01.366, CAM223 (B)	JX914760
Campanula armena Steven	Armenia, Suynik, Ghurghulagh, G. Fayvush et al. 03-0806, CAM192 (B)	JX914731
Campanula armena Steven	Russia, Czarzogljan s.n., CAM615 (B)	JX915077
Campanula arvatica Lag.	NCC 94003, 5T (EGHB)	JX915113
Campanula arvatica Lag.	Spain, Palencia, Cardano de Arriba, F. Amich et al. 17457, CAM488 (B)	JX914967

Campanula asperuloides (Boiss. & Orph.) Engler	Greece, Nomos Laconia, Sparti, Ulrich s.n., CAM224 (B)	JX914761
Campanula asperuloides (Boiss. & Orph.) Engler	Greece, Nomos Laconia, Elonis Monastery, G. Mansion 10043, CAM395 (B)	JX914915
Campanula asperuloides subsp. *taygetea* (Quezel & Contandr.) Greuter & Burdet	Greece, Nomos Laconia, Langadas gorges, G. Mansion 10075, CAM399 (B)	JX914919
Campanula asperuloides subsp. *taygetea* (Quezel & Contandr.) Greuter & Burdet	Greece, NC3209	JX915182
Campanula aucheri A. DC.	Eddie 95010, 6T (EGHB)	JX915114
Campanula aucheri A. DC.	Turkey, North Anatolia, Cubr 42996, CAM217 (B)	JX914754
Campanula aurita Greene	NC3371	JX915217
Campanula axillaris Boiss. & Balansa	Turkey, C4 Antalya, Alanya, S. Yildirimli 4449, CAM319 (GAZI)	JX914842
Campanula balansae Boiss. & Hausskn.	Turkey, C3 Isparta, Sütcüler, H Pesmen & A. Güner 2117, CAM320 (GAZI)	JX914843
Campanula balfourii Wagner & Vierh.	N. Killian et al. YP3990, CAM287 (B)	JX914812
Campanula barbata L.	Switzerland, Graubuenden, Oberengadin, Rost 48, CAM201 (B)	JX914740
Campanula barbata L.	Italy, Lombardia, Sondrio, Dürbye 2579, CAM487 (B)	JX914966
Campanula barbata L.	GenBank	FN396989*
Campanula barbata L.	Pistarino 1448, NC3327 (MRSN)	JX915199
Campanula baumgartenii Becker	GenBank	FN396991*
Campanula bayerniana Rupr.	Without locality, CAM300 (BG Berlin)	JX914825
Campanula bayerniana Rupr.	Armenia, Caucaus, S. Tamamschian s.n., CAM522 (LE)	JX914994
Campanula bayerniana Rupr.	Iran, N of Savalan, Javanshir 1261, CAM599 (TARI)	JX915068
Campanula bellidifolia Adams	Gaskin 115, 7T (MO)	JX915115
Campanula bellidifolia Adams	Georgia, Caucasus, Kazbegi, J. Stone et al 1893, CAM523 (LE)	JX914995
Campanula bellidifolia subsp. *argunensis* (Rupr.) Viktorov	Russia, Dagestan, Risrueva s.n., CAM524 (LE)	JX914996
Campanula bellidifolia subsp. *besenginica* (Fomin) Viktorov	Russia, Kabardino-Balkaria, Nikite sn, CAM525 (LE)	JX914997
Campanula bellidifolia subsp. *meyerana* (Rupr.) Viktorov	Azerbaijan, Caucasus, Popova et al, sn, CAM540 (LE)	JX915011
Campanula bellidifolia subsp. *saxifraga* (Bieb) Viktorov	GenBank	FN397038*
Campanula bertolae Colla	Italy, G. Bono s.n., CAM608 (FI)	JX915073
Campanula betonicifolia Sm.	Turkey, A2 Bursa, Uludag, Pamirkivogela & Quezel s.n., CAM321 (HUB)	JX914844
Campanula blumelii Halda	Turkey, Taurus Mountains, Bolkardag, JJ. Halda s.n., CAM659 (PR)	JX915098
Campanula blumelii Halda	Turkey, C3 Antalya, ENE Manavgat, R. Ulrich 7.15, CAM661 (B)	JX915100
Campanula bohemica Hruby	USSR, Krkonosa, S. Fröhner 5176, CAM464 (B)	JX914951
Campanula bononiensis L.	Greece, Nomos Pella, Ep. Edhessis, E. Willing 21.242, CAM191 (B)	JX914730
Campanula bononiensis L.	Russia, Altayskiy Kray, Yuzhnyy, Martins 2270, CAM270 (B)	JX914799
Campanula bononiensis L.	GenBank	FN396983*
Campanula bononiensis L.	Pistarino 1859, NC3328 (MRSN)	JX915200
Campanula bornmuelleri Nabelek	Turkey, B9 Van, Yan-Bahcesarai, Güner 7850, CAM383 (HUB)	JX914903
Campanula bornmuelleri Nabelek	Turkey, B9 Bitlis, Tatuan, A. Dönmez 10971, CAM622 (HUB)	JX915081
Campanula bravensis (Bolle) A.Chev.	Cape Verde Islands, Fogo, Cha das Caldeiras, N. Killian 3326, CAM190 (B)	JX914729
Campanula bravensis (Bolle) A.Chev.	Cape Verde Islands, Fogo, Cha das Caldeiras, Killian & Leyens 3278, CAM225 (B)	JX914762
Campanula bravensis (Bolle) A.Chev.	GenBank	FN396993*
Campanula buseri Damboldt	Turkey, C3 Antalya, ENE Manavgat, G. & R. Ulrich 7.15b, CAM660 (B - Herbarium GP)	JX915099
Campanula calaminthifolia Lam.	Greece, Nomos Dodecanese, Koronos, N. Böhling 1798, CAM188 (B)	JX914727
Campanula calcarata Somm. Et Levier	Georgia, Abkhazia Territory, Bramwell sn, CAM527 (LE)	JX914999
Campanula calcicola WW Sm.	China, Yunnan, Dali, Cang-shan, CAM628 (KUN)	JX915084
Campanula californica (Kellog) Heller	USA, California, San Francisco Bay, MG10132, CAM312 (B)	JX914835
Campanula camptoclada Boiss.	Israel, Dead Sea Valley, Mizpe Dragot, Danin et al. 06.056, CAM207 (B)	JX914746
Campanula cana Wall	China, CAM627 (KUN)	JX915083
Campanula cana Wall	China, TT Yu 3329, CAM632 (KUN)	JX915086
Campanula cana Wall	China, CAM633 (KUN)	JX915087
Campanula cana Wall	China, CAM634 (KUN)	JX915088
Campanula candida A. DC.	Iran, Kermanshah, Road to Kamyaran, Savafi 84388, CAM582 (TARI)	JX915051
Campanula candida A. DC.	Iran, Kernanshah, Road to kamyaran, Anonymous 90258, CAM583 (TARI)	JX915052
Campanula carnica Schiede ex Mert. & Koch	Italy, Venetie, Treviso, CAM299 (BG Berlin)	JX914824
Campanula carnica Schiede ex Mert. & Koch	NC3181	JX915166
Campanula carpatha Halácsy	GenBank	FN396995*
Campanula carpatha Halácsy	Avramakis s.n., NC3339 (NHMC)	JX915209
Campanula carpatica Jacq.	Slovakia, Drevenik, Gartenherbar, Cubr 43344, CAM218 (B)	JX914755
Campanula carpatica Jacq.	NC3391	JX915231
Campanula carpatica Jacq	CAM123 (B)	JX914675
Campanula cashmeriana Benth.	Central Asia, Tian-shan, Brichmulla, Vasak s.n., CAM214 (B)	JX914751
Campanula cashmeriana Benth.	India, Kashmir, Lidder Valley, Schwerdtfeger 23932, CAM219 (B)	JX914756
Campanula cashmeriana Benth.	Russia, CAM528 (LE)	JX915000
Campanula caucasica Bieb.	Armenia, CAM485 (B)	JX914965
Campanula caucasica Bieb.	Armenia, Gabrielan et al sn, CAM529 (LE)	JX915001
Campanula celsii A. DC.	Greece, Nomos Attica, Hymettos, U. Raabe s.n., CAM187 (B)	JX914726
Campanula celsii A. DC.	Greece, Nomos Arcadia, Eisenblätter & Willing 43.939, CAM226 (B)	JX914763
Campanula celsii A. DC.	Greece, Nomos Attica, Rd from Cape Sounion to Athen, G. Mansion 10008, CAM384 (B)	JX914904
Campanula celsii subsp. *parnesia* Phitos	Greece, Nomos Attica, Mt Parnitha. G. Mansion 10010, CAM386 (B)	JX914906
Campanula celsii subsp. *spatulifolia* (Turrill) Phitos	Greece, Nomos Corinthia, Before Loutraki, G. Mansion 10017, CAM389 (B)	JX914909
Campanula cenisia L.	Switzerland, Valais, Zermatt, R. Ro 58, CAM186 (B)	JX914725
Campanula cenisia L.	Poggio 1960, NC3342 (MRSN)	JX915211
Campanula cervicaria L.	GenBank	FN396997*
Campanula cervicaria L.	GenBank	FN396998*
Campanula cervicaria L.	GenBank	FN396999*
Campanula cervicaria L.	NC1414	JX915160
Campanula cervicaria L.	NC3229	JX915196
Campanula cespitosa Scop.	GenBank	FN396994*
Campanula cespitosa Scop.	NC0047	JX915155

Campanula cespitosa Scop.	NC3178	JX915163
Campanula chamissonis	USA, Washington, MtTahoma Nursery, Grown from seed, HAB	JX915135
Campanula choruhensis Kit Tan & Sorger	Turkey, N. İkinci & O. İkinci 3710, CAM454	JX914695
Campanula ciliata Stev.	GenBank	FN397001*
Campanula circassica Fomin	Russia, Stravopol, Dolmatova et al,, CAM530 (LE)	JX915002
Campanula cochlearifolia Lam.	NC3176	JX915161
Campanula cochleariifolia Lam.	Italy, Lombardy, Sondrino, Dürbye 2632, CAM199 (B)	JX914738
Campanula collina Sims	GenBank	FN397003*
Campanula collina subsp. *collina*	Turkey, Ag Artuin: Şauşat, Altinozlü 4228, CAM324 (HUB)	JX914845
Campanula collina subsp. *fondervisii* (Albov) Ogan.	Georgia, Abkhazia Territory, Tchitanava sn, CAM531 (LE)	JX915003
Campanula columnaris Contandr. et al.	Greece, Sterea Ellas, Nomos Fokidos, T. Raus et al 32151-5, CAM409 (B)	JX914928
Campanula conferta A. DC.	Turkey, B7 Erzincan, Kenaliya, MUJ 2048, CAM325 (HUB)	JX914846
Campanula constantini Beauverd & Top.	Greece, Sterea Ellas, Nomos Evvias, Central Evvia island, T. Raus et al 32194-05, CAM412 (B)	JX914931
Campanula coriacea PH Davis	Armenia, Oganesian s.n., CAM290 (B)	JX914815
Campanula crassipes Heffel	Greece, Crete, E. Mayer et al. 11968, CAM484 (B)	JX914964
Campanula crenulata Franch.	China, CAM644 (KUN)	JX915093
Campanula cretica (A. DC.) D. Dietr.	Greece, Crete, Nomos Chanion, N. Böhling 8586, CAM483 (B)	JX914963
Campanula cretica (A. DC.) D. Dietr.	Greece, Crete, Cellinese 1031, NC3338 (YU)	JX915208
Campanula creutzburgii Greuter	Greece, Nomos Irakliou, Kefalas, Böhling 7138, CAM227 (B)	JX914764
Campanula creutzburgii Greuter	Cellinese 1003, NC3340 (YU)	JX915210
Campanula crispa Lam.	Turkey, A9 Kars, Ardahan, Demirkus 2275, CAM328 (HUB)	JX914848
Campanula crispa Lam.	WMM Eddie 96067, HAB (TEX)	JX915136
Campanula cymaea Phitos	Greece, Sterea Ellas, Nomos Evvias, Central Evvia island, T. Raus et al 32190-05, CAM411 (B)	JX914930
Campanula cymaea Phitos	Greece, NC3206	JX915181
Campanula cymbalaria Sm.	Turkey, C5 Nigde, Aladaglari, M. Döring 1539, CAM183 (B)	JX914724
Campanula cymbalaria Sm.	NC3396	JX915235
Campanula daghestanica Fomin	Russia, Dagestan, Cheitski et al, CAM532 (1981) (LE)	JX915004
Campanula damascena Labill.	Israel, Mt. Carmel, Neshev, A. Danin 1992, CAM182 (B)	JX914723
Campanula damboldtiana PH Davis & Sorger	Turkey, Ankara, Kazan, Adiguzel 1993, CAM329 (GAZI)	JX914849
Campanula damboldtiana PH Davis & Sorger	Turkey, Ankara, Ayasbeli, M. Yural 4164, CAM619 (GAZI)	JX915079
Campanula dasyantha Bieb.	Russia, khabarovsk, Charkevich s.n., CAM534 (LE)	JX915006
Campanula dasyantha subsp. *chamissonis* (Fed.) Viktorov	Russia, Sakhalin Island, G. Ponomareczuk, CAM533 (LE)	JX915005
Campanula davisii Turrill	Turkey, C4 Antalya, Geyik Daglari, Döring et al. 6744, CAM215 (B)	JX914752
Campanula davisii Turrill	Turkey, C4 Antalya, Gundogmus, Güner 12587, CAM330 (HUB)	JX914850
Campanula delavayi Franch.	China, CAM643 (KUN)	JX915092
Campanula delicatula Boiss.	Turkey, C3 Antalya, Ancient site of Aspendos, G. Mansion 10001 & G. Parolly, CAM277 (B)	JX914805
Campanula delicatula Boiss.	Turkey, C2, Mugla:Dalyon Sülüngür, Gölü Ortaca, 15m sarp güney yenacler, Güner 8540, CAM331 (HUB)	JX914851
Campanula demirsoyi Kandemir	Turkey, B7 Erzincan, Munzur Daglar, Kandemir 6992, CAM332 (HUB)	JX914852
Campanula dichotoma L. f.	Turkey, B6, Göksun, Yildiz 2971, CAM333 (HUB)	JX914853
Campanula dimorphantha Schweinf.	China, H. Li et al 0035, CAM630 (KUN)	JX915085
Campanula divaricata Michx.	GenBank	FN397005*
Campanula dolomitica Busch	Georgien, Dusheti, Greater Caucasus, CAM535 (1998) (LE)	JX915007
Campanula drabifolia Sm.	Greece, Nomos West Attica, before Mytikas, G. Mansion 10014, CAM385 (B)	JX914905
Campanula drabifolia Sm.	Greece, Nomos Laconia, Rd to Gytheio, G. Mansion 10056, CAM396 (B)	JX914916
Campanula drabifolia Sm.	NC3195	JX915174
Campanula dzaaku Albov	Georgia, Abkhazia Territory, Tchitchanova sn, CAM536 (LE)	JX915008
Campanula dzychrica Kolak.	Georgia, Tobova s.n., CAM537 (LE)	JX915009
Campanula edulis Forssk.	GenBank	FN397006*
Campanula edulis Forssk.	NC3397	JX915236
Campanula ekimiana Güner	Turkey, Ankara, Kizikahamam, Mecit Vural 9719, CAM334 (GAZI)	JX914854
Campanula ekimiana Güner	Turkey, A. Güner et al 5629, CAM616 (HUB)	JX915078
Campanula elatines L.	Ayers-88-287, 12T (BH)	JX915116
Campanula elatines L.	Croatia, CAM111 (BG Bonn)	FN397007*
Campanula elatinoides Moretti	GenBank	FN397008*
Campanula erinus L.	Cyprus, Lysos, R. Hand 2284, CAM181 (B)	JX914722
Campanula erinus L.	Greece, Nomos Argolis, Epidavros, Ancient Amphitheater, G. Mansion 10031, CAM391 (B)	JX914911
Campanula erinus L.	Iran, Kermansha, Parow mountain above Bisotun, Hamzehee 87781, CAM585 (TARI)	JX915054
Campanula erinus L.	Cellinese 1028, NC0518 (YU)	JX915158
Campanula erinus L.	Cellinese 1028, NC3334 (YU)	JX915206
Campanula euboica Phitos	Greece, Sterea Ellas, Nomos Evvias, NW Evvia island, T. Raus et al 32244-05, CAM414 (B)	JX914933
Campanula excisa Schleich. ex Murith	Switzerland, Valais, Furgstalden, Van Buggenhout 14512, CAM482 (B)	JX914962
Campanula excisa Schleich. ex Murith	NC3187	JX915169
Campanula exigua Rattan	USA, California, Mt Diablo, MG10133, CAM313 (B)	JX914836
Campanula exigua Rattan	R. Haberle 145, HAB (TEX)	JX915137
Campanula fastigiata Dufour	Cyprus, Aradippou, R. Hand 5450, CAM258 (B)	JX914787
Campanula fenestrellata Feer	Croatia, Ledenik, Velebit montes, Cernoch 38869, CAM213 (B)	JX914750
Campanula fenestrellata Feer	GenBank	FN397007*
Campanula fenestrellata subsp. *istriaca* (Feer) Dambolt	Croatia, Cres, T. Wraber 124260, CAM170 (B)	JX914712
Campanula fenestrellata subsp. *istriaca* (Feer) Dambolt	NC3227	JX915194
Campanula filicaulis Durieu	Morocco, Timahdite, Meknès, Valdes et al. 16-0628, CAM279 (B)	JX914806
Campanula flaccidula Vatke	Turkey, C10 Hakkari: Şemdinli'den Şapatan Geçidine, A. Dönmez 10786, CAM335 (HUB)	JX914855
Campanula floridana Watson	M.Strong & C.L. Kelloff 1337, HAB (GMU)	JX915138
Campanula foliosa Ten.	Greece, Nomos Kastoria, Flambouro, E. Willing 6218, CAM180 (B)	JX914721
Campanula foliosa Ten.	GenBank	FN397010*

Campanula formanekiana Degen & Dörfler	Greece, Nomos Kilkis, Kastaneri, E. Willing 7600, CAM179 (B)	JX914720
Campanula formanekiana Degen & Dörfler	Greece, Nomos Pella, Notia, A. Schuler 99.1024, CAM189 (B)	JX914728
Campanula forsythii (Arcang.) Podl.	Scortegagna 5411, NC057 (MRSN)	JX915159
Campanula fragilis Cirillo	Eddie 98015, 14T (EGHB)	JX915117
Campanula fragilis Cirillo	GenBank	FN397011*
Campanula fragilis Cirillo	NC3398	JX915237
Campanula fruticulosa (O. Schwarz & PH Davis) Damboldt	Turkey, C2 Burdur, Dirmil, Duman 6279, CAM336 (GAZI)	JX914856
Campanula garganica Ten.	Italy, Monte S. Angelo, Aldobrandi & Baldini 18.842, CAM211 (B)	JX914749
Campanula garganica Ten.	NC3192	JX915171
Campanula giesekiana Vest	Greenland, Diako, Disko Fjord,Eqalunguit, Dalgaard 88.175, CAM210 (B)	JX914748
Campanula glomerata L.	Greece, Nomos Ioannina, Metsovo, R. Eisenblätter & E. Willing 49.833, CAM178 (B)	JX914719
Campanula glomerata L.	Russia, Altai Republic, Inya, Raab-Straube 020098, CAM209 (B)	JX914747
Campanula glomerata L.	GenBank	FN397012*
Campanula glomerata L.	NC0058	JX915156
Campanula glomerata L. [var. *cordifolia* Rohlena]	Rumenia, Sibiu, Valea Viilor, Gartenherbar, Cubr 42151, CAM220 (B)	JX914757
Campanula glomerata subsp. *caucasica* (Tratv.) Ogan.	Georgia, kasbegi, Nehmiski sn, CAM541 (LE)	JX915012
Campanula glomerata subsp. *caucasica* (Tratv.) Ogan.	Iran, Gilan, Lahijan, Siahkal, Jamzad & Asri 71774, CAM598 (TARI)	JX915067
Campanula glomerata subsp. *hispida* (Witasek) Hayek	Turkey, A8, Erzurum,Tortum, Güngörmez, Gremineae stebi, G. Akaydin 9561, CAM337 (HUB)	JX914857
Campanula glomerata subsp. *oblongifolioides* (Galushko) Ogan.	Russia, Karachaevo-Cherkessia, Keltman et al, sn, CAM543 (LE)	JX915014
Campanula glomerata subsp. speciosa	Russia, Nedoluchko et al. sn, CAM544 (LE)	JX915015
Campanula goulimyi Turrill	Greece, Sterea Ellas, Nomos Evvias, Central Evvia island, T. Raus et al 32198-05, CAM413 (B)	JX914932
Campanula grandis subsp. *rizeensis* (Gèner) Lammers	Turkey, A8, Trabzon, Sürmene, Güner et al 4917, CAM338 (HUB)	JX914858
Campanula griffinii Morin	USA, California, Bohemian Road, CAM315 (B)	JX914838
Campanula grossheimii Kharadze	Gaskin 206, 15T (MO)	JX915118
Campanula grossheimii Kharadze	GenBank	FN397013*
Campanula hagielia Boiss.	Turkey, C1 Mugla, Dalyan Kaunos, T. Raus 17793, CAM177 (B)	JX914718
Campanula hagielia Boiss.	Turkey, C2 Mugla, Ortaca, A. Güner et al. 10386, CAM620 (HUB)	JX915080
Campanula hagielia Boiss.	NC3204	JX915179
Campanula hawkinsiana Hausskn. & Heldr.	Eddie 94002, 16T (EGHB)	JX915119
Campanula hawkinsiana Hausskn. & Heldr.	Greece, Nomos Ioannina, Driskos E. Willing 158.725, CAM176 (B)	JX914717
Campanula hawkinsiana Hausskn. & Heldr.	Greece, Thessaly, Nomos Trikalon, Mt Pindos, T. Raus et al 32323-14, CAM420 (B)	JX914939
Campanula hawkinsiana Hausskn. & Heldr.	NC3214	JX915186
Campanula hedgei PH Davis	Turkey, B7 Tunceli, Tunceli-ovacih arass, Duman 8752, CAM339 (AEF)	JX914859
Campanula herminii Hoffmanns. & Link	Neves 227, 17T (TEX)	JX915120
Campanula heterophylla L.	Greece, Nomos Kikladhon, Insel Paros, T. Raus 20505 CAM175 (B)	JX914716
Campanula hierapetreae Rech. f.	Greece, Crete, Nomos Lasithiou, Ag. Ioannis, N. Böhling 8949, CAM174 (B)	JX914715
Campanula hierapetreae Rech. f.	GenBank	FN397014*
Campanula hierapetreae Rech. f.	Edwards 27, NC3333 (YU)	JX915205
Campanula hierosolymitana Boiss.	Jordania, Wadi Mujib, Christoph Bayer, s.n., CAM285 (B)	JX914811
Campanula hispanica Willk.	Spain, Tejada, Pico valdosa, Casas et al. 42, CAM206 (B)	JX914745
Campanula hofmannii (Pant.) Greuter & Burdet	GenBank	FN397092*
Campanula humillima A. DC.	Iran, Esfahan, Mountains S. of Damaneh , Assadi & Ranjbar 83065, CAM587 (TARI)	JX915056
Campanula hypopolia Trautv.	GenBank	FN397016*
Campanula iconia Phitos	H. Morath 346, CAM340	JX914860
Campanula incanescens Boiss.	Russian label, CAM539 (LE)	JX915010
Campanula incanescens Boiss.	Russian label, CAM545 (LE)	JX915016
Campanula incanescens Boiss.	Iran, Bandar Abba, Hajiabad, Fareghan, Mozaffarian 74264, CAM588 (TARI)	JX915057
Campanula incanescens Boiss.	Iran, Fars, Shirz, Darengan, Mozaffarian 83653, CAM589 (TARI)	JX915058
Campanula incurva Aucher ex A. DC.	Greece, Nomos Thessaloniki, Magnisia, Tsangaranda, CAM297 (BG Berlin)	JX914822
Campanula incurva Aucher ex A. DC.	GenBank	FN397017*
Campanula incurva Aucher ex A. DC.	NC3215	JX915187
Campanula involucrata Aucher ex A. DC.	Turkey, C4 Antalya, Geyik Daglari, M. Döring et al. 6827, CAM172 (B)	JX914714
Campanula isaurica Contandriopoulos et al.	Turkey, C3 Antalya, Beskonak, P. Hein A 45D-4, CAM171 (B)	JX914713
Campanula isaurica Contandriopoulos et al.	Turkey, C3 Antalya, Beskonak, Hein A56-4, CAM205 (B)	JX914744
Campanula isophylla Moretti	GenBank	FN397018*
Campanula jacobaea C.Sm. ex Webb	Cape Verde Islands, Santo Antao, Ribeira Paul, K. Killian & T. Leyens 2989, CAM169 (B)	JX914711
Campanula jacobaea C.Sm. ex Webb	GenBank	FN397019*
Campanula jacquinii (Sieber) A. DC.	Greece, Crete, Nomos Heraklion, Monofatsiou, R. Jahn s.n., CAM168 (B)	JX914710
Campanula jacquinii (Sieber) A. DC.	Greece, Thessaly, Nomos Pierias, Mt Olympus, T. Raus et al 32260, CAM416 (B)	JX914935
Campanula jacquinii (Sieber) A. DC.	Edwards 41, NC3332 (YU)	JX915204
Campanula kadargavinica Amirkh. & Komzha	Georgia, Ossetia Territory, Amijanov sn, CAM546 (LE)	JX915017
Campanula karakuschensis Grossh.	Azerbaijan, Bazorgan, Rechinger 44029, CAM204 (B)	JX914743
Campanula karakuschensis Grossh.	Masoumi & Safavi 82552, CAM586 (IRAN)	JX915055
Campanula keniensis Thulin	Kenya, Ngong hills, Magadi, Gachathi 76/60, CAM601 (B)	JX915070
Campanula kermanica (Rech. f. et al) Rech. f.	Iran, Kermansha, Jiroft, Foroughi 15962, CAM590 (TARI)	JX915059
Campanula khorasanica (Rech. f. & Aellen) Rech. f.	Iran, Semnan province, Touran protected Area, Freitag 14842, CAM476 (B)	JX914961
Campanula khorasanica (Rech. f. & Aellen) Rech. f.	Iran, Khorosan, Bojnrd, Memariani & Zangooie 37502, CAM591 (TARI)	JX915060
Campanula kirikkaleensis Dönmez & Güner	Turkey, B5 Kirikkale, Delice cevresi, A. Dönmez 3924 [Holotypus], CAM341 (HUB)	JX914861
Campanula kolenatiana C.A.Mey.	Azerbaijan, CAM548 (LE)	JX915019
Campanula kolenatiana C.A.Mey.	Azerbaijan, Korotkov s,n., CAM549 (LE)	JX915020
Campanula kolenatiana C.A.Mey.	GenBank	FN397020*
Campanula komarovii Maleev	Russia, Kelendjik et al, sn, CAM547 (LE)	JX915018
Campanula koyuncui H.Duman	Turkey, C2 Mugla, Babadag, Eren 5154, CAM203 (B)	JX914742
Campanula koyuncui H.Duman	Turkey, C2 Mugla, Fethiye, baba Dag, H. Duman 5728, CAM342 (GAZI)	JX914862

Campanula kremeri Boiss. & Reut.	Morocco, Djebel Israne/ Djebel Achoun E Taforalt, Oued Zegzel, R. Vogt & C. Oberprieler 11475, CAM475 (B)	JX914960
Campanula kryophila Rupr.	Russia, Nehmiski sn, CAM550 (LE)	JX915021
Campanula laciniata L.	Greece, Nomos Dodecanese, Karpathos, Arkasa, Höner & Potthoff 628, CAM202 (B)	JX914741
Campanula laciniata L.	NC3331	JX915203
Campanula lactiflora M.Bieb.	Eddie 95009, 36T (EGHB)	JX915131
Campanula lactiflora M.Bieb.	GenBank	FN397021*
Campanula lamondiae Rech. f.	Persia, Kurdistan, Mount Hamzeh Arab, KH. Rechinger 42629, CAM474 (B)	JX914959
Campanula lamondiae Rech. f.	Iran, Iranshahr & Dezfolian 3590, CAM592 (IRAN)	JX915061
Campanula lanata Friv.	Eddie 96051, 18T (EGHB)	JX915121
Campanula lanata Friv.	Bulgaria, M. Markova & L. Cerneva 1078, CAM466 (B)	JX914953
Campanula lasiocarpa Cham.	Canada, British Columbia, Bella Coola, Volker Michelfelder 1, CAM302 (UBC)	JX914827
Campanula lasiocarpa Cham.	NC3372	JX915218
Campanula lasiocarpa Cham.	NC3375	JX915221
Campanula latifolia L.	Germany, Bavaria, Munich, Muhr et al s.n., CAM262 (B)	JX914791
Campanula latifolia L.	GenBank	FN397022*
Campanula lavrensis (Tocl & Rohlena) Phitos	Greece, Nomos Chalkidiki, Longos, KH. Rechinger 44659, CAM473 (B)	JX914958
Campanula legionensis Pau	Spain, Prov. Santander, La Hermida, Ladero & Valle 12558, CAM246 (B)	JX914778
Campanula lehmanniana Bunge	Russia, CAM552 (LE)	JX915023
Campanula lehmanniana subsp. *pseudohissarica* Kamelin ex Rassulova	Russian label, CAM553 (LE)	JX915024
Campanula leucantha Gilli	Afghanistan, H. Freitag 3031, CAM614	JX915076
Campanula leucoclada Boiss.	Afghanistan, Kabul, Valley of Maidan, KH. Rechinger 35989, CAM471 (B)	JX914957
Campanula leucosiphon Boiss. & Heldr.	Turkey, C4 Antalya, Karaman-ermenek arasi, Quezel et al 223, CAM344 (HUB)	JX914864
Campanula lingulata Waldst. & Kit.	Greece, Nomos Messenia, Kalamon, O Ladhas, R. & E. Willing 92.968, CAM165 (B)	JX914707
Campanula lingulata Waldst. & Kit.	Turkey, A1 Kırklareli: DereKöy'den Kırklareli'ne, A. Dönmez 7030, CAM345 (HUB)	JX914865
Campanula lingulata Waldst. & Kit.	NC3201	JX915176
Campanula lingulata Waldst. & Kit.	NC3228	JX915195
Campanula longistyla Fomin	Russia, Krasnodevi sn, CAM554 (LE)	JX915025
Campanula lourica Boiss.	Persia, Teheran, Mount Elburz, KH. Rechinger 57153, CAM470 (B)	JX914956
Campanula lourica Boiss.	Iran, Golestan, Jahan- Nama, S. M. Jafari 2232, CAM593 (TARI)	JX915062
Campanula luristanica Freyn	Iran, Esfahan, Shelgerd, Daran, Iranshahr & Moussavi 3525, CAM594 (TARI)	JX915063
Campanula lusitanica Loefl.	Neves 226, 20T (TEX)	JX915122
Campanula lusitanica Loefl.	Spain, La Riuoja, Sierra de la Demanda, Loidi & Berastegui 17459, CAM239 (B)	JX914772
Campanula lyrata Lam.	Turkey, C2, Mugla, Agla, Dörning 25, CAM238 (B)	JX914771
Campanula lyrata Lam.	NC3212	JX915185
Campanula lyrata subsp. *icarica* Runemark	NC3205	JX915180
Campanula macrochlamys Boiss. & Huet	Turkey, A9 Erzurum, Kaya 9032, CAM381 (GAZI)	JX914901
Campanula macrochlamys Boiss. & Huet	Turkey, A9 Kars, Göle, Karliyazi, Demikus 1062, CAM625 (HUB)	JX915082
Campanula macrorhiza Gay ex A. DC.	Germany, Botanical Garden Berlin, Schwerdtfeger 8906, CAM465 (B)	JX914952
Campanula macrorhiza Gay ex A. DC.	NC0059	JX915157
Campanula macrorhiza Gay ex A. DC.	NC3189	JX915170
Campanula macrostachya Waldst. & Kit.	Greece, Nomos Thessaloniki, Evangilistria, E. Willing 21.104, CAM164 (B)	JX914706
Campanula macrostachya Waldst. & Kit.	Turkey, A Kirklaeli, Denirköy, Ekici 3324, CAM382 (GAZI)	JX914902
Campanula macrostachya Waldst. & Kit.	NC3219	JX915188
Campanula macrostyla Boiss. & Heldr.	Turkey, C4 Icel, Kirobasi, R.Ulrich s.n., CAM289 (B)	JX914814
Campanula marchesettii Witasek	Croatia, Istria, Ucka, T. Gottschlich 33737, CAM163 (B)	JX914705
Campanula marchesettii Witasek	NC3182	JX915167
Campanula mardinensis Bornm. & Sint.	Turkey, C9, Siirt, A. Dönmez 11212, CAM346 (HUB)	JX914866
Campanula massalskyi Fomin	Armenia, Oganesian s.n., CAM493 (B)	JX914972
Campanula medium L.	GenBank	FN397024*
Campanula mekongensis Diels ex CY Wu	China, CAM641 (KUN)	JX915091
Campanula micrantha Bertol.	Italy, Umbrien, Monte Sibillini, Dürbye 937, CAM236 (B)	JX914770
Campanula micrantha Bertol.	Scaramozzino 1630, NC3326 (MRSN)	JX915198
Campanula mirabilis Albov	Eddie 96056, 22T (EGHB)	JX915123
Campanula mirabilis Albov	Georgia, Abkhazia Territory, n/a, CAM555 (LE)	JX915026
Campanula moesiaca Velen.	Greece, Nomos Dramas, Rhodopi mountains, Strid et al. 19458, CAM235 (B)	JX914769
Campanula mollis L.	Neves 230, 23T (TEX)	JX915124
Campanula mollis L.	Spain, Vites, Valdes et al. 2184 /88, CAM234 (B) (under C. velutina)	JX914768
Campanula morettiana Reichenb.	Italy, Sèdtirol, Schlern, E. Zippel, CAM664 (B)	JX915102
Campanula myrtifolia Boiss. & Heldr.	Turkey, A8 Artvin, Altiparmak, Ulrich s.n., CAM240 (B)	JX914773
Campanula myrtifolia Boiss. & Heldr.	Turkey, Igel, Anamur-Kazanci, Miruval 7143, CAM347 (GAZI)	JX914867
Campanula nuristanica Rech. f. & Schiman-Czeika	Afghanistan, H. Freitag 5975, CAM613	JX915075
Campanula odontosepala Boiss.	Iran, Mazandaran, Chalus, Gabrielian s.n., CAM232 (B)	JX914767
Campanula oligosperma Damboldt	Turkey, B7, Vilayet, Tunceli, Holtz et al. 00.768, CAM231 (B)	JX914766
Campanula oligosperma Damboldt	Turkey, B7 Tunceli, Pulumur, Adiguzel NA4117, CAM675 (GAZI)	JX915106
Campanula olympica Boiss.	Turkey, A4 Cankiri, Küçük Ilgaz Daği, T. Raus 6878, CAM162 (B)	JX914704
Campanula olympica Boiss.	Turkey, A4 Çankırı, Ilgaz, Ilgaz Dağı, A. Dönmez 11713, CAM348 (HUB)	JX914868
Campanula oreadum Boiss. & Heldr.	Greece, Thessaly, Nomos Pierias, Mt Olympus, T. Raus et al 32247-03, CAM415 (B)	JX914934
Campanula orphanidea Boiss.	Greece, Nomos Serres, Spanakokorifi, E. Willing 10.621, CAM160 (B)	JX914702
Campanula orphanidea Boiss.	Greece, Makedonia, Nomos Kavalas, Mt Pangeon, T. Raus et al 32267-10, CAM417 (B)	JX914936
Campanula ossetica Bieb.	Russia, Averianov et al. s.n., CAM558 (LE)	JX915028
Campanula ossetica Bieb.	Russia, CAM559 (LE)	JX915029
Campanula pallida Wall. [var. pallida]	Pakistan, Northern areas, Nanga Parbat, Nüsser 275, CAM161(B)	JX914703
Campanula pallida Wall. [var. pallida]	China, CAM635 (KUN)	JX915089
Campanula pallida Wall. [var. pallida]	China, L. Ende and F. Wei 2162, CAM636 (KUN)	JX915090

Campanula pallida Wall. [var. pallida]	China, Li Heng s.n., CAM646 (KUN)	JX915095
Campanula pallida Wall. [var. tibetica (Hooker f. & Thomson) Hara]	Pakistan, Northern areas, Nanga Parbat, Nüsser 1174, CAM159 (B)	JX914701
Campanula pangea Hartvig	Greece, Makedonia, Nomos Kavalas, Mt Pangeon, T. Raus et al 32268-05, CAM418 (B)	JX914937
Campanula papillosa Halácsy	Greece, Nomos Laconia, Taygetos Mountains, C. Kyriakopoulos T227-1, CAM673 (UPA, B)	JX915104
Campanula papillosa Halácsy	Greece, Nomos Laconia, Taygetos Mountains, C. Kyriakopoulos T227-2, CAM674 (UPA, B)	JX915105
Campanula paradoxa Kolak.	Georgia, Bzyb river, M. Oganisian 7131, CAM560 (1978) (LE)	JX915030
Campanula patula L.	Switzerland, Graubuenden, Puschlav Resena, T. Eckard 2046a, CAM498 (B)	JX914974
Campanula patula L.	GenBank	FN397025*
Campanula patula L.	GenBank	FN397026*
Campanula patula subsp. *costae* (Willk.) Nyman	Italy, Valle d'Aosta, Arnad, CAM296 (BG Berlin)	JX914821
Campanula patula subsp. *epigaea* (Janka) Hyek	Montenegro, Verusa, G. Parolly s.n., CAM497 (B)	JX914973
Campanula pelia (Halacsy) Hausskn. & Sint.	Greece, Nomos Magnisias, Ep. Volou, E. Willing 26.054, CAM148 (B)	JX914689
Campanula pelviformis Lam.	Greece, Crete, Nomos Lasithiou, Thripti, Böhling & Raus 7215, CAM256 (B)	JX914785
Campanula pendula Bieb.	Russia, Stravopol,Piatigorsk, Popova sn, CAM575 (LE)	JX915044
Campanula peregrina L.	Eddie 95007, 25T (TEX)	JX915125
Campanula peregrina L.	Cyprus, Mavrokolympos brook, R. Hand 2190, CAM158 (B)	JX914700
Campanula perpusilla A. DC.	Iran, Khouzestan, Izeh, Abkhogan, Riazi 9484, CAM595 (TARI)	JX915064
Campanula perpusilla A. DC.	Iran, Khouzestan, NE of Dezful, Jamzad & Mordi 79198, CAM596 (TARI)	JX915065
Campanula persicifolia L.	Eddie 95027, 26T (TEX)	JX915126
Campanula persicifolia L.	Greece, Nomos Thessaloniki, Ajios Ioannis, V. Karragiannakidou s.n., CAM157 (B)	JX914699
Campanula persicifolia L.	Greece, Nomos Serres, Pangeon, Aj. Ikosifinissis, E. Willing 22.435, CAM167 (B)	JX914709
Campanula persicifolia L.	NC3386	JX915226
Campanula persicifolia L. subsp. *sessiliflora* (K.Koch) Fed.	Greece, Nomos Kozani, Vourinos, Höner & Potthoff 764a, CAM257 (B)	JX914786
Campanula peshmenii Güner	Turkey, B6 Malatya, Dogansehir, H. Pesmen 2717 (Holotypus), CAM351 (HUB)	JX914871
Campanula petraea L.	Daccordi 6347, NC3335 (MRSN)	JX915207
Campanula petraea L.	Italy, Veneto, Madonna della Corona, M. Tamanini, CAM611 (FIR)	JX915074
Campanula petrophila Rupr.	Russia, T. Monoba s.n., CAM520 (LE)	JX914992
Campanula petrophila Rupr.	GenBank	FN397029*
Campanula phrygia Jaub. & Spach	Greece, Nomos Pella, SO Promahi, R. & E. Willing 128.966, CAM156 (B)	JX914698
Campanula phyctidocalyx Boiss. & Noë	Azerbaijan, Urmia, Pesan, Marmishu valley, Mozaffarian 87231, CAM597 (TARI)	JX915066
Campanula pinatzii Greuter & Phitos	Greece, Nomos Dodecanese, Palatia, N. Böhling 8244, CAM155 (B)	JX914697
Campanula pinnatifida Huber-Morath	Turkey, B6, Kayseri: Pinarbasi-sanz yolu, G. Akaydin 10419, CAM352 (GAZI)	JX914872
Campanula pinnatifida Huber-Morath	Turkey, A4, Cankiri: Günclogdu mevkii, Cankiri ünv. Kampüs alani, Jipsli aragi, G. Akaydin 6579, CAM353 (HUB)	JX914873
Campanula pinnatifida Huber-Morath	Turkey, B6 Kayseri, Pinabasi-Sariz, Adiguzel 4192, CAM354 (GAZI)	JX914874
Campanula piperi Howell	USA, Washington, MtTahoma Nursery, Grown from seed, HAB	JX915139
Campanula podocarpa Boiss.	Turkey, C2 Mugla, Kéycegiz, A. Güner 8613, CAM676 (HUB)	JX915107
Campanula polyclada Rech. F. & Schiman-Czieka	Afghanistan, Panjao, KH. Rechinger 36652, CAM501 (B)	JX914975
Campanula pontica Albov	Turkey, A7 Trabrom, Macka, Eyaboglu 1030, CAM356 (GAZI)	JX914876
Campanula portenschlagiana Schultes	GenBank	FN397030*
Campanula portenschlagiana Schultes	NC3222	JX915190
Campanula portenschlagiana Schultes	NC3390	JX915230
Campanula poscharskyana Degen	Yugoslavia, Dalmatia, Cavtatcca, Mayer 11152, CAM254 (B)	JX914784
Campanula prenanthoides Durand	USA, California, Ponderosa Way, MG10138, CAM311 (B)	JX914834
Campanula propinqua Fischer & C. A. Meyer	Turkey, B7, Malatya: Malatya-Pötürge, 26km nach Abzweigung, Nydegger 17211, CAM357 (HUB)	JX914877
Campanula pseudostenocodon Lacaita	Italy, Prov. L Aquila, Arischia, Merxmüller C220, CAM503 (B)	JX914976
Campanula psilostachya Boiss. & Kotschy	Turkey, C5, Adana: Karsanti, Acima, CAM358 (HUB)	JX914878
Campanula ptarmicifolia Lam.	Turkey, B7 Ercincan, Kemah, A. Dönmez 5373, CAM359 (HUB)	JX914879
Campanula ptarmicifolia Lam.	Turkey, B7 Tunceli, Ovacik, Yildirimli 3448, CAM678 (HUB)	JX915108
Campanula pterocaula Hausskn.	Turkey, A3 Bolu, Yenicaga, Nydegger 19005, CAM251 (B)	JX914783
Campanula pubicalyx (PH Davis) Damboldt	Turkey, Icel, Karanci-Abanos, Vurnl 7164, CAM360 (GAZI)	JX914880
Campanula pubicalyx (PH Davis) Damboldt	Turkey, Mitural 7164, CAM679	JX915109
Campanula pulla L.	Austria, Steiermark, Hochschwabgruppe, U. & D. Mueller-Doblies 8159, CAM504 (B)	JX914977
Campanula pulvinaris Hausskn. & Bornm.	Turkey, B6, Sivas: Gemerek; Kayababa Dagi, O. Zudogun 1507, CAM361 (HUB)	JX914881
Campanula punctata Lam.	Russia, Vrisch sn, CAM561 (LE)	JX915031
Campanula pyramidalis L.	Eddie 96089, 27T (EGHB)	JX915127
Campanula pyramidalis L.	GenBank	FN397031*
Campanula pyramidalis L.	NC3224	JX915192
Campanula pyramidalis L.	NC3387	JX915227
Campanula quercetorum Huber-Morath & C. Simon	Turkey, B7 Tunceli, Tunceli-Ovacik yolu, Albinozlü 3801, CAM362 (HUB)	JX914882
Campanula radchensis Kharadze	Georgia, Distr. Ratscha, R. Gagnidze, I. Mikeladze, CAM562 (1966) (LE)	JX915032
Campanula raddeana Trautv.	Without locality, CAM301 (BG Berlin)	JX914826
Campanula radicosa Bory & Chaub.	Greece, Nomos Korinthias, Ziria Massiv, E. Willing 5113, CAM154 (B)	JX914696
Campanula radula Fisch	Turkey, B9 Mu-odiye-Böküh, T. Ekim 7719, CAM363 (GAZI)	JX914883
Campanula raineri Perpenti	Italy, Lombardy, E Laggo di Lecco, Dürbye 2517, CAM153 (B)	JX914694
Campanula ramosissima Sm.	Greece, Nomos Arta, S Kapsala, R. & E. Willing 138.546, CAM152 (B)	JX914693
Campanula ramosissima Sm.	Greece, Nomos Laconia, Gheraki, G. Mansion 10047, CAM393 (B)	JX914913
Campanula ramosissima Sm.	Greece, Nomos Laconia, Mystra Fortress, G. Mansion 10069, CAM397 (B)	JX914917
Campanula ramosissima Sm.	Greece, Nomos Corinthia, Road to Trikala, G. Mansion 10091, CAM556 (B)	JX915027
Campanula rapunculoides L.	Jansen 8/00-007, 28T (TEX)	JX915128
Campanula rapunculoides L.	GenBank	FN397032*
Campanula rapunculoides L.	NC3223	JX915191
Campanula rapunculoides L.	NC3393	JX915233

Campanula rapunculus L.	Greece, Nomos Evrou, Kallithea, T. Raus et al. 21517, CAM166 (B)	JX914708
Campanula reiseri Halácsy	Greece, Nomos Dodecanese, Yioura, between Skala and Vala, D. Phitos & G. Kamari 20333, CAM200 (B)	JX914739
Campanula reuteriana Boiss. & Balansa	Turkey, B7, Erzincan: Kemah, Kömürköy yukarisi, Aslan 3389, CAM364 (GAZI)	JX914884
Campanula reuteriana Boiss. & Balansa	Iran, kermanshah, Polezahab, Anonymous 2273, CAM584 (TARI)	JX915053
Campanula reverchonii A. Gray	Eddie 00004, 29T (TEX)	JX915129
Campanula rhodensis A. DC.	Greece, Nomos Dodekanisou, Rhodos, Böhling 9695, CAM249 (B)	JX914781
Campanula rhomboidalis L.	GenBank	FN397032*
Campanula robinsiae Small	Bok Gardens, Cheryl Peterson s.n., HAB	JX915140
Campanula romanica Savul	Romenia, Baltagesti, Dealul Allah-Bair, Cristurean s.n., CAM248 (B)	JX914780
Campanula rotundifolia L.	USA, New Mexico, Carson National Forest, Schmidt et al 2709, CAM247 (B)	JX914779
Campanula rotundifolia L.	Russia, Altai Republic, Yaylyu, L. Martins 2526, CAM275 (B)	JX914803
Campanula rotundifolia L.	Russia, Khabarovsk territory, Ayan-Maysk, S. Kharkevich 172668, CAM308 (UBC)	JX914832
Campanula rotundifolia L.	Canada, British Columbia, Sparwood area, O. Lee V209011, CAM309 (UBC)	JX914833
Campanula rotundifolia L.	Russia, Koroleva s.n.. CAM551 (LE)	JX915022
Campanula rotundifolia L.	NC3179	JX915164
Campanula rotundifolia L.	NC3180	JX915165
Campanula rotundifolia L.	NC3389	JX915229
Campanula rumeliana (Hampe) Vatke	Greece, Nomos Dramas, Mt. Orvilos, T. Raus et al. 21276, CAM133 (B)	FN397035*
Campanula rumeliana (Hampe) Vatke	Bulgaria, Slavjanka mt., B. Kuzmanov 802845, CAM508 (B)	JX914980
Campanula rumeliana (Hampe) Vatke	NC3211	JX915184
Campanula rumeliana susbp. *chalcidica* (Buser) Greuter & Burdet	NC3202	JX915177
Campanula rupestris Sm.	Greece, Nomos Fokis, Ep. Parnassidhos, E. Willing 15.358a, CAM144 (B)	JX914686
Campanula rupestris Sm.	Greece, Nomos Phocis, Parnassos Mountains, ski resort, G. Mansion 10105, CAM401 (B)	JX914921
Campanula rupestris Sm.	NC3226	JX915193
Campanula rupicola Boiss. & Spruner	Greece, Nomos Phthiotis, Parnassos, R. Eisenblätter & E. Willing 46.300 CAM143 (B)	JX914685
Campanula rupicola Boiss. & Spruner	Greece, Sterea Ellas, Nomos Fthiotidos, Mt Parnassos,T. Raus et al 32083, CAM404 (B)	JX914924
Campanula sabatia De Not.	Italy, Prov. Savona, Capo di Noli, L. Poldini 12559, CAM505 (B)	JX914978
Campanula sabatia De Not.	Italy, Prov. Savona, Alpi Liguri Meridionali, Rocca Barbena, E. Martini s.n., CAM604 (FIR)	JX915072
Campanula samothracica (Degen) Greuter & Burdet	Greece, Nomos Evrou, Eparchia Samothrakis, T. Raus 21347, CAM142 (B)	JX914684
Campanula sarmatica Ker Gawl.	Gaskin 458, 31T (MO)	JX915130
Campanula sarmatica Ker Gawl.	Without locality, CAM293 (BG Berlin)	JX914818
Campanula sarmatica subsp. *woronowii* (Kharadze) Ogan.	Russian label, CAM563 (LE)	JX915033
Campanula sartorii Boiss. & Heldr.	GenBank	FN397037*
Campanula saxatilis L.	Greece, Nomos Irakliou, Eparchia Kenourgiou, Lendas, Böhling & Greuter 7828, CAM244 (B)	JX914777
Campanula saxifragoides Doumergue	Morocco, High Atlas, CAM295 (BG Berlin)	JX914820
Campanula saxonorum Gand.	Turkey, Usak, Hagemann et al 2652, CAM243 (B)	JX914776
Campanula saxonorum Gand.	Turkey, C4, Antalya: Alanya-Manavgat yolu, Manavgata, G. Akaydin 10265, CAM355 (HUB)	JX914875
Campanula saxonorum Gand.	Turkey, C8 Siirt, Saglarca, Aslan 3644, CAM365 (GAZI)	JX914885
Campanula scabrella Engelm.	NC3378	JX915224
Campanula scheuchzeri Vill.	GenBank	FN397039
Campanula scheuchzeri Vill.	NC3177	JX915162
Campanula sciathia Phitos	NC3392	JX915232
Campanula sclerotricha Boiss.	Azerbaijan, Sardasht, Rechinger 49075, CAM242 (B)	JX914775
Campanula sclerotricha Boiss.	Turkey, C6 Kahramanmaras, Engizek & Duman 426, CAM366 (GAZI)	JX914886
Campanula scoparia (Boiss. & Hausskn.) Damboldt	Turkey, C6, Malatya, Doganshir, Erkenek, Aktoglu 861, CAM367 (INU)	JX914887
Campanula scoparia (Boiss. & Hausskn.) Damboldt	Turkey, B7, Ercincan, Kenalyie, Kirkgoz, Ekim 8133, CAM680 (GAZI)	JX915110
Campanula scopelia Phitos	Greece, Nomos Magnisias, Ep. Skopelou, Skopelos, S. & B. Snogerup 5587, CAM141 (B)	JX914683
Campanula scouleri Hooker ex A. DC.	Canada, British Columbia, Vancouver Island, CAM307 (UBC)	JX914831
Campanula scutellata Griseb.	Greece, Nomos Kilkis, Ep. Peonias, Piji, E. Willing 17.880, CAM140 (B)	JX914682
Campanula seraglio Kit Tan & Sorger	Turkey, A8 Artvin, Altiparmak, Döhring et al 7291, CAM241 (B)	JX914774
Campanula serrata (Kit.) Hendrych	No information, CAM403 (BG Berlin)	JX914923
Campanula serrata subsp. *recta* (Dulac) Podlech	France, Dép. Puy-de-Dome, Super Besse, Hand 1033, CAM250 (B)	JX914782
Campanula shetleri Heckard	USA, California, Crags Trail, MG10119, CAM314 (B)	JX914837
Campanula sibirica L.	Rumania, Valea Lui David, P. Anastasiu s.n., CAM139 (B)	JX914681
Campanula sibirica L.	Russia, Altai Republic, Onguday, Martins 2362, CAM276 (B)	JX914804
Campanula sibirica subsp. *brassicifolia* (Sommier & Levier) Ogan.	Georgia, Svaneti, Mestia, Kolchicova sn, CAM564 (LE)	JX915034
Campanula sibirica subsp. *charadzae* (Grossh.) Ogan.	Russia, Chechnya, Geltman et al sn, CAM565 (LE)	JX915035
Campanula sibirica subsp. *ciscaucasica* (Kharadze) Ogan.	Russia, Elbroz, Nehmiski sn, CAM566 (LE)	JX915036
Campanula sibirica subsp. *divergens* (Waldst.) Nyman	Bulgaria, NE of Nessebar, C. Gussev & R. Rutherford, CAM284 (B)	JX914810
Campanula sibirica subsp. *hohenackeri* (Fisch. & Mey.) Dambolt	Turkey, A8, Erzurum, Ispir, G. Akaydin 9813, CAM368 (HUB)	JX914888
Campanula similans Carlström	Turkey, C1 Mugla, near Kapikiri/ Herakleia, T. Raus 17792, CAM138 (B)	JX914680
Campanula sparsa Friv.	Greece, Nomos Trikala, Kalambakas, Nozia Pindhos, R. Eisenblätter & E. Willing 59.434, CAM137 (B)	JX914679
Campanula sparsa Friv.	NC3203	JX915178
Campanula spatulata Sm.	Greece, Nomos Karditsa, NW Rendina, R. & E. Willing 163.005-163.03, CAM132 (B)	FN397040*
Campanula spatulata Sm.	Greece, Nomos Corinthia, Sofiko, G. Mansion 10021, CAM387 (B)	JX914907
Campanula spatulata Sm.	Cellinese 1005, NC3230 (YU)	JX915197
Campanula spatulata subsp. *filicaulis* (Halacsy) Phitos	Greece, Crete, Nomos Lasithiou, Sitias, Lenika, N. Böhling 5076, CAM134 (B)	JX914676
Campanula spatulata subsp. *spruneriana* (Hampe) Hayek	Cellinese 1005, NC3193 (YU)	JX915172
Campanula speciosa Pourret	Spain, Prov. Tarragona, Ports de Tortosa, Romo 9008, CAM288 (B)	JX914813
Campanula speciosa subsp. *affinis* (Schult.) Font Quer	Spain, Barcelona, Anoia, C. benedi & J. Moleno 18, CAM492 (B)	JX914971
Campanula spicata L.	GenBank	FN397044
Campanula stellaris Boiss.	Israel, Kinnrot Valley, NE Kibbutz Haon, Danin et al. 39.045, CAM198 (B)	JX914737

Campanula stenosiphon Boiss. & Heldr.	Greece, Nomos Laconia, Langadas gorges, G. Mansion 10074, CAM398 (B)	JX914918
Campanula stevenii Bieb.	Turkey, Agri, Tendürek Dagh, T. Raus 4372, CAM146 (B)	JX914687
Campanula stevenii Bieb.	NC3394	JX915234
Campanula stevenii subsp. *alberti* (Trauv.) Viktorov	Russian label, CAM567 (LE)	JX915037
Campanula stevenii subsp. *altaica* (Ledeb.) Fed.	Russia, Altai Republic, Pereval Seminskiy, E. Raab-Straube 020031, CAM196 (B)	JX914735
Campanula stevenii subsp. *stevenii*	Georgia, Gardabani district, Environs Kojori, Zanojurashrili 3059, CAM372 (HUB)	JX914892
Campanula stevenii subsp. *wolgensis* (P.A. Smirn.) Fed.	Russian Label, CAM265 (B)	JX914794
Campanula stevenii susbp. *beauverdiana* (Fomin) Rech. F. & Schima-Czeika	Turkey, A8, Erzurum, Tortum, Yagcilar yaylasi, G. Akaydin 9582, CAM371 (HUB)	JX914891
Campanula stevenii susbp. *turczaninovii* (Fed.) Viktorov	GenBank	FN397053*
Campanula stricta L.	Turkey, C2 Mugla, Sandras Dagi, M. Döring et al. 81, CAM151 (B)	JX914692
Campanula stricta L.	Turkey, B5 Nigda, Gardak-ürgüp yolu, 10km voltanik tüflü arazi, G. Akaydin 6672b, CAM373 (HUB)	JX914893
Campanula stricta L. [var. libanotica]	Turkey, C3 Antalya, Kerner, M. Döring et al. 420, CAM147 (B)	JX914688
Campanula strigillosa Boiss.	Turkey, B6 Sivas, Gök Pinar, Sorger 762111, CAM264 (B)	JX914793
Campanula strigillosa Boiss.	Turkey, B6, Sivas, Sarkisla, Karababa Dagi, KO. Zudogun 1404, CAM374 (HUB)	JX914894
Campanula strigosa Banks & Solander	Turkey, B7, Erzincan, Kenallye, Gesobell-Venk yolu, Hareketti, yanaclo, HA 5300, CAM375 (HUB)	JX914895
Campanula suanetica Rupr.	Georgia, Svaneti, Lachveti & Kimeritze s.n., CAM568 (LE)	JX915038
Campanula suanetica Rupr.	Georgia, S. Stekeranzi s.n., CAM657 (LE)	JX915097
Campanula takesimana Nakai	Without locality, CAM291 (BG Berlin)	JX914816
Campanula takesimana Nakai	No Information, CAM681 (BG Berlin)	JX915111
Campanula tanfanii Podl.	Italy, Prov L Aquila Abruzzo, Colle Caciaro, M. Iberite et al. 17461, CAM263 (B)	JX914792
Campanula telephioides Boiss. & Hausskn.	Turkey, C6, Kahramanmaras, Aher Dagi, Aytan 1992, CAM376 (GAZI)	JX914896
Campanula telmessi Huber-Morath & Phitos	Turkey, C3 Antalya, ENE Manavgat, R. Ulrich 7.8, CAM663 (B)	JX915101
Campanula teucrioides Boiss.	Turkey, B2 İzmir: Bozdağ, Kayak merkezinden zirveye tırmanış, A. Dönmez 11499, CAM377 (HUB)	JX914897
Campanula thyrsoides L.	GenBank	FN397046*
Campanula thyrsoides L.	GenBank	FN397047*
Campanula tokurii Ocak	Turkey, B3 Afyon, Suhut, E. Akacek 3694 (Paratypus), CAM349 (GAZI)	JX914869
Campanula tokurii Ocak	Turkey, B3 Kütahya: Sabuncu, Fındık-İncik Köyleri arası, A. Dönmez 9983, CAM350 (HUB)	JX914870
Campanula tokurii Ocak	Turkey, B3 Eskisehir, Tèrkmen Daglari, Ocak 9051 (Isotypus), CAM378 (GAZI)	JX914898
Campanula tomentosa Lam.	Turkey, C1 - Aydin, Priene, T. Raus 14578, CAM149 (B)	JX914690
Campanula tomentosa Lam.	GenBank	FN397048*
Campanula tommasiniana Koch	Croatia, Istria, Opatija, Ucka, G. Gottschlich 33738, CAM150 (B)	JX914691
Campanula topaliana Beauverd	Greece, Nomos Argolis, Rd to Epidavros, G. Mansion 10020, CAM388 (B)	JX914908
Campanula topaliana Beauverd subsp. *cordifolia* Phitos	Greece, Nomos Laconia, Kilikini Plateau, G. Mansion 10096, CAM602 (B)	JX915071
Campanula topaliana Beauverd subsp. *delphica* Phitos	Greece, Nomos Fokidos, Sterea Ellas, T. Raus 24307, CAM221 (B)	JX914758
Campanula topaliana Beauverd subsp. *delphica* Phitos	Greece, Nomos Phocis, Delphi Sanctuary, G. Mansion 10103, CAM400 (B)	JX914920
Campanula trachelium L.	GenBank	FN397049*
Campanula trachelium L.	GenBank	FN397050*
Campanula trachelium L. subsp. *athoa* (Boiss. & Heldr.) Hayek	Greece, Nomos Larissa, Dimos Efrimeno, Stomio, A. Schuler 480, CAM136 (B)	JX914678
Campanula trachyphylla Schott & Kotschy ex Boiss.	Turkey, C5 Nigde, W Meydan, P. Hein A 244-4, CAM135 (B)	JX914677
Campanula trichocalycina	Greece, Nomos Karditsa, R. & E. Willing 142.206, CAM130 (B)	FN397051
Campanula tridentata Schreber	Turkey, A8 Erzurum: Kayak merkezi yukarısı, bozkır, A. Dönmez 12323, CAM343 (HUB)	JX914863
Campanula tridentata Schreber	GenBank	FN397052*
Campanula tridentata subsp. *biebersteiniana* (Schult.) Ogan.	Russia, Dagestan, Galushko s.n., CAM526 (LE)	JX914998
Campanula trista Kitam	Pakistan, Swat, between Barikot and Mingora, KH. Rechinger 30491, CAM506 (B)	JX914979
Campanula troegerae Damboldt	Turkey, Artvin, Yusufeli, Öğdem yolu üzeri, N. & O. İkinci 3700, CAM444 (AIBU, B)	JX914945
Campanula troegerae Damboldt	Turkey, Artvin, Yusufeli, Öğdem yolu üzeri, N. & O. İkinci 3703, CAM447 (AIBU, B)	JX914946
Campanula tubulosa Lam.	Greece, Nomos Lasithiou, Eparchia Ierapetras, Ag. Ioannis, N. Böhling 8316, CAM461 (B)	JX914949
Campanula tymphaea Hausskn.	Greece, Nomos Ioannina, Metsovou, Willing 83481, CAM462 (B)	JX914950
Campanula uniflora L.	Russia, Chukotka, Petrovski sn, CAM569 (LE)	JX915039
Campanula uyemurae (Kudo) Miyabe & Tatew.	Russia, Sakhalin, Sea of Okhotsk, Penomatchuk s,n,, CAM570 (LE)	JX915040
Campanula velebitica Borbás	Greece, Nomos Pellis, Eparchia Almopias, B. Pirker et al. 142, CAM469 (B)	JX914955
Campanula veneris Carlström	Cyprus, Christodoulou s.n., CAM230 (B)	JX914765
Campanula versicolor Andrews	GenBank	FN397054*
Campanula versicolor Andrews	NC3194	JX915173
Campanula versicolor Andrews	NC3388	JX915228
Campanula waldsteiniana Schult.	Croatia, Tulove Grede, Mount Velebit australes, Cernoch 43753, CAM261 (B)	JX914790
Campanula wilkinsiana Greeene	NC3374	JX915220
Campanula wilkinsiana Greeene	NC3376	JX915222
Campanula willkommii Witasek	Spain, Sierra de Baza, Valdes et al 132488, CAM260 (B)	JX914789
Campanula witasekiana Vierh.	Austria, Ostalpen, Steiermark, between Glashuetten and Steinberg, Widder s.n., CAM259 (B)	JX914788
Campanula witasekiana Vierh.	NC3186	JX915168
Campanula witasekiana Vierh.	NC3221	JX915189
Campanula xylocarpa Kovanda	Slovakia, Cubr 41318, CAM222 (BG Berlin)	JX914759
Campanula yaltirikii Duman	Turkey, C2 Antalya, Elmali, Ciglikara mevkii,, Duman 5977 (Isotypus), CAM379 (GAZI)	JX914899
Campanula yildirimlii Kit Tan & Sorger	Turkey, B7, Ercincan, Kenalyie, Kirkgoz, Syo 2980, CAM380 (HUB)	JX914900
Campanula zangezura (Lipsky) Kolak.	Without locality, CAM402 (BG Berlin)	JX914922
Campanula zangezura (Lipsky) Kolak.	Georgia, Abkhazia Territory, Nehmiski sn, CAM576 (LE)	JX915045
Campanula zoysii Jacq.	Slovenia, Juliske Alpe, M. Lovka et al. 974814, CAM468 (B)	JX914954
CANARINA		

Canarina canariensis (L.) Vatke	GenBank	FN397055*
Canarina eminii Aschers.et Schweinf.	GenBank	FN397056*
CODONOPSIS		
Codonopsis lanceolata (Siebold & Zucc.) Trautv.	GenBank	FN397057*
CYANANTHUS		
Cyananthus lobatus Wall. ex Benth.	GenBank	FN397058*
CYCLOCODON		
Cyclocodon lancifolius (Roxb.) Kurz	GenBank	FN397059*
CYPHIA		
Cyphia lisiandra Diels	GenBank	FN397060*
Cyphia subtubulata Wimm.	GenBank	FN397061*
Cyphia tysonii Phillips	GenBank	FN397062*
EDRAIANTHUS		
Edraianthus graminifolius (L.) A.DC.	Greece, Thessaly, Nomos Pierias, Mt Olympus, T. Raus et al 32248-04, CAM410 (B)	JX914929
Edraianthus pumilio (Port.) A.DC.	GenBank	FN397063*
Edraianthus tenuifolius (Waldst. & Kit.) A. DC.	GenBank	FN397064*
Edrianthus graminifolius	Italy, Sicily, Strada Quacella, s.n., NC3351 (YU)	JX915215
FEERIA		
Feeria angustifolia (Schousb.) Buser	Morocco, Beni Mellal, road 508 between Afrouer and Bir-el-Quidane, R. Vogt 11946, CAM266 (B)	JX914795
GITHOPSIS		
Githopsis diffusa A. Gray	N. Morin s.n., HAB	JX915141
Githopsis diffusa subsp. *robusta* Morin	USA, California, La Moine, MG10120, CAM422 (B)	JX914941
Githopsis pulchella Vatke	Morin s. n., 38T (OS)	JX915132
Githopsis pulchella Vatke	USA, California, Schott Rd, MG10139, CAM423 (B)	JX914942
Githopsis specularioides Nutt.	USA, California, Billger Creek Rd, MG10115, CAM421 (B)	JX914940
GRAMMATOTHECA		
Grammatotheca bergiana (Cham.) Presl.	GenBank	FN397066*
HANABUSAYA		
Hanabusaya asiatica (Nakai) Nakai	Eddie 95018, 39T (EGHB)	JX915148
HETEROCODON		
Heterocodon rariflorus Nutt.	Haberle 149, 40T (TEX)	JX915149
Heterocodon rariflorus Nutt.	USA, California, Schott Road, MG10144, CAM316 (B)	JX914839
HIPPOBROMA		
Hippobroma longiflora G.Don	Without locality, N. Korotkova s.n., CAM120 (BG BONN)	JX914672
ISOSTOMA		
Isotoma axillaris Lindl.	GenBank	FN397067*
Isotoma fluviatilis F.Muell. ex Benth.	GenBank	FN397068*
JASIONE		
Jasione crispa	Eddie 95083, 41T (EGHB)	JX915150
Jasione heldreichii Boiss. & Orph.	T. Ayers 88-208, 42T (BH)	JX915151
Jasione heldreichii Boiss. & Orph.	Greece, Makedonia, Nomos Kavalas, Keramoti, T. Raus et al 32282, CAM419 (B)	JX914938
Jasione laevis Lam.	GenBank	FN397069*
Jasione montana L.	Sales and Hedge 9898, 44T (EGHB)	JX915152
Jasione montana L.	Pistarino 1738, NC3352 (MRSN)	JX915216
Jasione montana L.	GenBank	FN397070*
Jasione montana subsp. *echinata* (Boiss. & Reut.) Nyman	Morocco, Tazzeka, bab-Bou-Idir, Valdes et al., CAM280 (B)	JX914807
LEGOUSIA		
Legousia falcata (Ten.) Fritsch	Cosner 143, 45T (OS)	JX915153
Legousia falcata (Ten.) Fritsch	Morocco, Middle Atlas, Azrou Ain-Leuh, Meknès, Valdes et al., CAM281 (B)	JX914808
Legousia pentagonia (L.) Thell.	R. Haberle 130, HAB (TEX)	JX915142
Legousia speculum-veneris (L.) Chaix	GenBank	FN397071*
LOBELIA		
Lobelia deckenii (Asch.) Hemsl.	GenBank	FN397072*
Lobelia inflata L.	GenBank	FN397073*
Lobelia nana Kunth	GenBank	FN397074*
Lobelia rhynchopetalum Hemsl	GenBank	FN397075*
Lobelia salicina Lam.	GenBank	FN397065*
MICHAUXIA		
Michauxia campanuloides L'Hér.	GenBank	FN397076*
Michauxia laevigata Vent.	Russia, CAM573	JX915042
Michauxia nuda A. DC.	Turkey, C8 Siirt, Eruh-yolu, Aslan 3802, CAM665 (B)	JX915103
Michauxia tchihatcheffii Fisch. & C.A.Mey.	Turkey, C5 Nigde, Ala Daglari, P. Hein A297-1, CAM509 (B)	JX914981
MUSSCHIA		
Musschia aurea (L.) Dum.	T. Ayers 88-274, 48T (BH)	JX915154
Musschia aurea (L.) Dum.	GenBank	FN397077*
Musschia wollastoni Lowe	GenBank	FN397078*
Musschia wollastoni Lowe	R. Haberle 227 (cultivar), HAB (PLU)	JX915143
NESOCODON		
Nesocodon mauritianus (I.B.K.Richardson) Thulin	GenBank	FN397079*
OSTROWSKIA		
Ostrowskia magnifica Regel	Russian label, CAM574 (LE)	JX915043
PETROMARULA		
Petromarula pinnata A.DC.	Agrini 038, NC3343 (MRSN)	JX915212
Petromarula pinnata A.DC.	GenBank	FN397081*
PHYSOPLEXIS		
Physoplexis comosa (Endl.) Schur.	Eddie 95008, 49T (EGHB)	JX915240
Physoplexis comosa (Endl.) Schur.	GenBank	FN397082*

PHYTEUMA		
Phyteuma betonicifolium Vill.	GenBank	FN397084*
Phyteuma hemisphaericum L.	GenBank	FN397085*
Phyteuma humile	Ferraris 5785, NC3345 (MRSN)	JX915214
Phyteuma orbiculare L.	GenBank	FN397083*
Phyteuma ovatum	Pistarino 5385, NC3344 (MRSN)	JX915213
Phyteuma scheuchzeri All.	GenBank	FN397086*
PLATYCODON		
Platycodon grandiflorum A.DC.	GenBank	FN397087*
PRATIA (LOBELIA)		
Pratia nummularia (Lam.) A.Braun & Aschers.	GenBank	FN397088*
PRISMATOCARPUS		
Prismatocarpus pedunculatus (P.J.Bergius) A.DC.	South Africa, Cape Province, Worcester, Goldblatt & Manning 8588, CAM267 (B)	JX914796
ROELLA		
Roella decurrens L'Hér.	South Africa, Cape Province, Cape town district, Table Mountains, Balkwill et al 8732, CAM268 (B)	JX914797
SIPHOCAMPYLUS		
Siphocampylus manettiiflorus Hook.	GenBank	FN397089*
SOLENOPSIS		
Solenopsis anthiphonitis	Cyprus, Division 7, Kalograia, R. Hand 5340, CAM651 (B)	JX915096
TRACHELIUM		
Trachelium caeruleum L.	Morocco, Aknoul, Taineste, Taza, Valdes et al 39-1346, CAM424 (B)	JX914943
TRIODANIS		
Triodanis coloradoensis (Buckley) McVaugh	R. Haberle 138, HAB (TEX)	JX915145
Triodanis leptocarpa (Nutt.) Nieuwl.	R. Haberle 132, HAB (TEX)	JX915146
Triodanis perfoliata (Nutt.) Nieuwl.	USA, Oregon, Eugene Arboretum, MG10111, CAM317 (B)	JX914840
Triodanis perfoliata (Nutt.) Nieuwl.	R. Haberle 167, HAB (TEX)	JX915147
Triodanis perfoliata subsp. *biflora* (Ruiz. & Pav.) Lammers	Argentinia, Faxinal, Parana, Cordeiro et al 1599, CAM269 (B)	JX914798
Triodanis perfoliata subsp. *biflora* (Ruiz. & Pav.) Lammers	R. Haberle 134, HAB (TEX)	JX915144
WAHLENBERGIA		
Wahlenbergia hederacea	Eddie & Wicks, 56T	JX915241
Wahlenbergia saxicola (Brown) A.DC.	GenBank	FN397093*
Wahlenbergia sp.	NC3198	JX915175
