# Supplementary material for: How to Handle Speciose Clades? Mass Taxon-Sampling as a Strategy towards Illuminating the Natural History of Campanula (Campanuloideae)
Source: PLoS One. 2012 Nov 28;7(11):e50076. doi: 10.1371/journal.pone.0050076 (PMC3509159; doi:10.1371/journal.pone.0050076)
Supplement: Table S2 — Overview of a potential infra-genetic classification of Campanula L. Type species used for the classification-guided sampling are indicated in bold green. (PDF) [file pone.0050076.s013.pdf]

**Genus**			**Campanula**	
	**Subgenus**		**Brachycodonia (Fed.) Damboldt (1976)**	
			***Campanula fastigiata* Dufour (*)**	*Campanula sivasica* Kit Tan & Yildiz
	**Subgenus**		**Campanula**	
		Section	Campanula = C. sect. Medium A. DC. Subsect. Eucodon (A. DC.) Fed. (1957) = C. sect. Trachelioideae (Boiss.) Kharadze (1949)	
			Campanula bononiensis L.	*Campanula rapunculoides* L.
			Campanula grossekii Heuffel	*Campanula trachelium* subsp. *athoa* (Boiss. & Heldr.) Hayek
			***Campanula latifolia* L. (*)**	*Campanula trachelium* subsp. *mauritanica* (Pomel) Quézel
			Campanula latifolia subsp. *megrelica* (Manden. & Kuth.) Ogan.	*Campanula trachelium* L. subsp. *trachelium*
			Campanula odontosepala Boiss.	
		Section	Cordifoliae (Fomin) Kharadze (1949) = C. sect. Medium A. DC. subsect. Cordifoliae (Fomin) Fed. (1957)	
			Campanula alliariifolia subsp. *letschchumensis* (Kem.-Nat.) Ogan.	*Campanula dolomitica* Busch
			***Campanula alliariifolia* Willd. (*)**	*Campanula sclerotricha* Boiss.
			Campanula axillaris Boiss. & Balansa (?)	
		Section	Elatines (Wohlfarth) Trinajstić	
			***Campanula elatines* L. (*)**	*Campanula garganica* Ten.
			Campanula elatinoides Moretti	*Campanula isophylla* Moretti
			Campanula fenestrellata Feer	*Campanula portenschlagiana* Schult.
			Campanula fenestrellata subsp. istriaca (Feer) Damboldt	*Campanula poscharskyana* Degen
			Campanula fragilis Cirillo	*Campanula reatina* Lucchese
			Campanula garganica subsp. *cephallenica* (Feer) Hayek	
		Section	Favratia (Feer) Kuntze	
			***Campanula zoysii* Wulfen (*)**	
		Section	Heterophylla (Witasek) Săvul. (1916) = C. sect. Medium A. DC. subsect. Heterophylla (Nyman) Fed. (1957)	
			Campanula alaskana (A. Gray) Wight	*Campanula marcenoi* Brullo
			Campanula albanica Witasek	*Campanula marchesettii* Witasek
			Campanula baumgartenii Becker	*Campanula micrantha* Bertol.
			Campanula baumgartenii subsp. *beckiana* (Hayek) Podlech	*Campanula moravica* (Spitzn.) Kovanda
			Campanula bertolae Colla	*Campanula petiolata* A. DC.
			Campanula bohemica Hruby	*Campanula pindicola* Aldén
			Campanula cantabrica Feer	*Campanula pollinensis* Podlech
			Campanula carnica Schiede ex Mert. & W.D.J. Koch	*Campanula praesignis* Beck
			Campanula carnica subsp. *puberula* Podlech	*Campanula precatoria* Timb.-Lagr.
			Campanula cespitosa Scop.	*Campanula pseudostenocodon* Lacaita
			Campanula cochleariifolia Lam.	*Campanula pulla* L.
			Campanula crassipes Heuffel	*Campanula raineri* Perpenti
			Campanula excisa Schleich. ex Murith	*Campanula rhomboidalis* L.
			Campanula ficarioides Timb.-Lagr.	*Campanula romanica* Săvul.

Campanula forsythii (Arcang.) Podlech
Campanula fritschii Witasek
Campanula gentilis Kovanda
Campanula giesekiana Vest
Campanula gracillima Podlech
Campanula hercegovina Degen & Fiala
Campanula herminii Hoffmanns. & Link
Campanula hispanica Willk.
Campanula intercedens Witasek
Campanula jaubertiana Timb.-Lagr.
Campanula jurjurensis Pomel
Campanula justiniana Witasek
Campanula longisepala Podlech
Campanula macrorrhiza Gay ex A. DC.
***Campanula rotundifolia* L. (*)**
Campanula ruscinonensis Timb.-Lagr.
Campanula sabatia De Not.
Campanula scheuchzeri Vill.
Campanula serrata (Kit.) Hendrych
Campanula serrata subsp. *recta* (Dulac) Podlech
Campanula stenocodon Boiss. & Reuter
Campanula tanfanii Podlech
Campanula tatrae Borbás
Campanula trojanensis Kovanda & Ančev
Campanula velebitica Borbás
Campanula willkommii Witasek
Campanula witasekiana Vierh.
Campanula xylocarpa Kovanda

Section Hypopolion (Fed.) Ogan. (1995)

***Campanula hypopolia* Trautv. (*)**

Section Involucratae (Fomin) Kharadze (1949) = C. sect. Medium A. DC. subsect. Involucratae (Fomin) Fed. (1957)

Campanula cervicaria L.
Campanula foliosa Ten.
***Campanula glomerata* L. (*)**
Campanula glomerata subsp. *caucasica* (Trautv.) Ogan.
Campanula glomerata subsp. *hispida* (Witasek) Hayek
Campanula glomerata subsp. *oblongifolioides* (Galushko) Ogan.
Campanula glomerata subsp. *speciosa*
Campanula glomerata subsp. *symphytifolia* (Albov) Ogan.
Campanula involucrata Aucher ex A. DC.
Campanula lingulata Waldst. & Kit.
Campanula macrochlamys Boiss. & A. Huet
Campanula macrostachya Waldst. & Kit.
Campanula moesiaca Velen.
Campanula pangea Hartvig
Campanula spicata L.
Campanula thyrsoides L.

Section Latilimbus (Fed.) Kharadze (1976) = C. sect. Medium A. DC. subsect. Latilimbus Fed. (1957)

Subsection Latilimbus Fed. (1957) = C. subsect. Latilimbus Fed. ser. Collinae Kolak. (1951)

***Campanula collina* Sims (*)**
Campanula collina subsp. *fondervisii* (Albov) Ogan.
Campanula collina subsp. *sphaerocarpa* (Kolak.) Ogan.
Campanula sclerophylla (Kolak.) Czer.

Subsection Sarmaticae (Kharadze) Victorov (2002) = C. subsect. Latilimbus Fed. ser. Sarmaticae Kharadze (1976)

***Campanula sarmatica* Ker Gawl. (*)**
Campanula sarmatica subsp. *calcarea* (Albov) Ogan.
Campanula sarmatica subsp. *ramosissima* (Sommier & Levier) Ogan.
Campanula sarmatica subsp. *woronowii* (Kharadze) Ogan.

Subsection Trigonophyllon Fed. (1957)

***Campanula dzychrica* Kolak. (*)**
Campanula jadvigae Kolak.
Campanula kolakovskyi Kharadze

Section Megalocodon Damboldt

Campanula incurva* Aucher ex A. DC.

Section Oreocodon (Fed.) Ogan. (1995) = C. sect. Medium A. DC. Subsect.Oreocodon Fed. (1957) = C. sect. Saxicolae (Boiss.) Kharadze (1949), p. p.

Campanula acutiloba Vatke
Campanula calycialata Randjel & Zlatkovic
Campanula cana Wall
Campanula candida A. DC.
Campanula cashmeriana Benth.
Campanula chinensis D.Y. Hong
Campanula conferta A. DC.
Campanula coriacea P.H. Davis
Campanula cymbalaria Sm.
Campanula damascena Labill.
Campanula escalerae Rech. f. & Schiman-Czeika
Campanula euclasta Boiss.
Campanula eugeniae Fed.
Campanula hedgei P.H. Davis
Campanula hermanii Rech. f.
Campanula humillima A. DC.
Campanula hystricula Pau
***Campanula incanescens* Boiss. (*)**
Campanula kachetica Kantsch.
Campanula kantschavelii Zagar.
Campanula kermanica (Rech. f. & al.) Rech. f.
Campanula khorasanica (Rech. f. & Aellen) Rech. f.
Campanula kiharae Kitam.
Campanula lehmanniana Bunge
Campanula lehmanniana subsp. *capusii* (Franch.) Viktorov
Campanula lehmanniana subsp. *pseudohissarica* Kamelin ex Rassulova
Campanula leucoclada Boiss.
Campanula lourica Boiss.
Campanula luristanica Freyn
Campanula mardinensis Bornm. & Sint.
Campanula massalskyi Fomin
Campanula nuristanica Rech. f. & Schiman-Czeika
Campanula pallida Wall. [var. pallida]
Campanula pallida Wall. [var. tibetica (Hooker f. & Thomson) Hara]
Campanula perpusilla A. DC.
Campanula persepolitana Kotschy ex Boiss.
Campanula polyclada Rech. f. & Schiman-Czeika
Campanula psilostachya Boiss. & Kotschy
Campanula quercetorum Huber-Morath & C. Simon
Campanula radicosa Bory & Chaub.
Campanula robertsonii Gamble
Campanula sartorii Boiss. & Heldr.
Campanula scoparia (Boiss. & Hausskn.) Damboldt
Campanula staintonii Rech. f. & Schiman-Czeika
Campanula sylvatica Wall
Campanula telephioides Boiss. & Hausskn.
Campanula trichopoda Boiss.
Campanula tristis Kitam.
Campanula versicolor Andrews

Section Paradoxae (Kolak.) Kolak. (1991) = C. sect. Medium A. DC. subsect. Paradoxae Kolak. (1976)

***Campanula paradoxa* Kolak. (*)**

Section Petrocodonia (Fed.) Ogan. (1995) = Symphyandra A. DC. Sect. Petrocodonia Fed. (1957)

Campanula lezgina (Alex.) Kolak. & Serdyuk

Section Phasadianthe (Fed.) Kharadze

Campanula imeretina Rupr.

Section Platysperma Damboldt

***Campanula oligosperma* Damboldt (*)**
Campanula ptarmicifolia Lam.
Campanula trachyphylla Schott & Kotschy ex Boiss.
Campanula munzurensis P.H. Davis

Section Sibiricae (Fomin) Kharadze (1949) = C. sect. Medium A. DC. subsect. Triloculares Boiss. ser. Sibiricae Fomin (1904)

Campanula caucasica M. Bieb.
Campanula daghestanica Fomin
Campanula komarovii Maleev
Campanula longistyla Fomin
***Campanula sibirica* L. (*)**
Campanula sibirica subsp. *brassicifolia* (Sommier & Levier) Ogan.
Campanula sibirica subsp. *charadzae* (Grossh.) Ogan.
Campanula sibirica subsp. *charkeviczii* (Fed.) Fed.
Campanula sibirica subsp. *ciscaucasica* (Kharadze) Ogan.
Campanula sibirica subsp. *divergens* (Waldst.) Nyman
Campanula sibirica subsp. *elatior* (Fomin) Fed.
Campanula sibirica subsp. *hohenackeri* (Fisch. & C.A. Mey.) Dambolt
Campanula sibirica L. subsp. *sibirica*
Campanula sibirica subsp. *talievii* (Juz.) Fed.
Campanula sibirica subsp. *taurica* (Juz.) Fed.

Section — Spinulosae (Fomin) Kharadze (1949) = C. sect. Medium A. DC. subsect. Triloculares Boiss. ser. Spinulosae Fomin (1904)

***Campanula mirabilis* Albov (*)**

Section — Symphyandriformes (Fomin) Kharadze (1949) = C. sect. Medium A. DC. subsect Symphyandriformes (Fomin) Fed. (1957)

Subsection — Osseticae Kharadze & Serdjukova (1973)

***Campanula ossetica* M. Bieb. (*)**

Subsection — Otocalyx (A. DC.) Victorov (2002) = Symphyandra A. DC. Sect. Otocalyx A. DC. (1839)

***Campanula armena* Steven (*)**
Campanula lazica (Boiss. & Balansa) Kharadze
Campanula pendula M. Bieb.
Campanula zangezura (Lipsky) Kolak.

Subsection — Symphyandriformes (Fomin) Fed. (1957) = C. sect. Medium A. DC. subsect. Triloculares Boiss. ser. Symphyandriformes Fomin (1905)

Campanula andina Rupr.
Campanula autraniana Albov
Campanula bayerniana Rupr.
Campanula betulifolia K. Koch
Campanula calcarata Sommier & Levier
Campanula choruhensis Kit Tan & Sorger
Campanula engurensis Kharadze
***Campanula kolenatiana* C.A. Mey. (*)**
Campanula raddeana Trautv.
Campanula seraglio Kit Tan & Sorger
Campanula suanetica Rupr.
Campanula troegerae Damboldt

Section — Tracheliopsis (Buser) Kuntze (1976)

***Campanula buseri* Damboldt (*)**
Campanula fruticulosa (O. Schwarz & P.H. Davis) Damboldt
Campanula myrtifolia Boiss. & Heldr.
Campanula postii (Boiss.) Engler
Campanula pubicalyx (P.H. Davis) Damboldt

Section — Trachelium (L.) Kuntze (1904)

Campanula asperuloides (Boiss. & Orph.) Engl.
Campanula asperuloides subsp. *taygetea* (Quezel & Contandr.) Greuter & Burdet
Campanula jacquinii (Sieber) A. DC.
Campanula rumeliana (Hampe) Vatke
***Trachelium caeruleum* L. (*)**

Section — Tulipella (Fed.) Victorov (2002) = C. sect. Medium A. DC. subsect. Tulipella Fed. (1957)

Campanula microdonta Koidz.
***Campanula punctata* Lam. (*)**
Campanula takesimana Nakai

Section — African group = Campanula s.l.

Campanula afganica Pomel
Campanula afra Cav.
Campanula atlantis Gattef. & al.
Campanula baborensis Quézel
Campanula bordesiana Maire
Campanula bravensis (Bolle) A. Chev.
Campanula dimorphantha Schweinf.
Campanula edulis Forssk.
Campanula embergeri Litard. & Maire
Campanula filicaulis Durieu
Campanula guinochetii Quézel
Campanula hypocrateriformis Dobignard
Campanula jacobaea C. Sm. ex Webb
Campanula jurjurensis Pomel
Campanula keniensis Thulin
Campanula kremeri Boiss. & Reut.
Campanula mairei Pau ex Maire
Campanula mollis L.
Campanula monodiana Maire
Campanula numidica Durieu
Campanula occidentalis Nyman
Campanula robertsonii Gamble
Campanula sauvagei Quézel
***Campanula saxifragoides* Doumergue (*)**
Campanula serhouchensis Dobignard
Campanula vaillantii Quézel
Campanula velata Pomel

Subgenus **Gadellia (Schulkina) Victorov (2002)**

***Campanula lactiflora* M. Bieb. (*)**

Subgenus **Megalocalyx Damboldt (1976)**

***Campanula balansae* Boiss. & Hausskn. (*)**
Campanula balfourii Wagner & Vierh.
Campanula camptoclada Boiss.
Campanula dichotoma L. f.
Campanula hierosolymitana Boiss.
Campanula hypocrateriformis Dobignard
Campanula propinqua Fisch. & C.A. Mey.
Campanula reuteriana Boiss. & Balansa
Campanula rimarum Boiss.
Campanula saxonorum Gand.
Campanula semisecta Murb.
Campanula stellaris Boiss.
Campanula strigosa Banks & Sol.
Campanula sulphurea Boiss.

Subgenus **Melanocalyx (Fed.) Victorov (2002)**

Campanula tschuktschorum Jurtz & Fed.
***Campanula uniflora* L. (*)**

Subgenus **Odontocalyx (Fed.) Victorov (2002)**

***Campanula lasiocarpa* Cham. (*)**

Subgenus **Pseudocampanula (Kolak.) Ogan. (1995) = Gen. Pseudocampanula Kolak.**

***Campanula dzaaku* Albov (*)**

Subgenus **Quinqueloculares (Boiss.) Phitos in Damboldt (1978) = C. sect. Medium A. DC. subsect. Quinqueloculares Boiss. (1875)**

Campanula anchusiflora Salisb. ex Sm.
Campanula andrewsii A. DC.
Campanula andrewsii subsp. *hirsutula* Phitos
Campanula betonicifolia Sm.
Campanula bordesiana Maire
Campanula carpatha Halácsy
Campanula celsii A. DC.
Campanula celsii subsp. *carystea* Phitos
Campanula lanata Friv.
Campanula lavrensis (Tocl & Rohlena) Phitos
Campanula lyrata Lam.
***Campanula medium* L. (*)**
Campanula merxmuelleri Phitos
Campanula nisyria Papatsou & Phitos
Campanula orphanidea Boiss.
Campanula pelviformis Lam.

Campanula celsii subsp. *parnesia* Phitos
Campanula celsii subsp. *spathulifolia* (Turrill) Phitos
Campanula crispa Lam.
Campanula davisii Turrill
Campanula euboica Phitos
Campanula goulimyi Turrill
Campanula hagielia Boiss.
Campanula iconia Phitos
Campanula karadjana Bocquet
Campanula laciniata L.
Campanula rechingeri Phitos
Campanula reiseri Halácsy
Campanula saxatilis L.
Campanula sorgerae Phitos
Campanula telmessi Huber-Morath & Phitos
Campanula tomentosa Lam.
Campanula topaliana Beauverd subsp. *topaliana*
Campanula topaliana subsp. *cordifolia* Phitos
Campanula topaliana subsp. *delphica* Phitos
Campanula tubulosa Lam.

Subgenus **Rapunculus (Boiss.) Kharadze (1976)**

Section Annae (Kolak.) Victorov (2002)

Campanula hieracioides Kolak.

Section Ponticae (Kharadze) Victorov (2002)

***Campanula pontica* Albov (*)**

Section Rapunculus Boiss. (1875)

Campanula aizoides Zaffran ex Greuter
Campanula aizoon Boiss. & Spruner
Campanula aristata Wall.
Campanula arvatica Lag.
Campanula cenisia L.
Campanula columnaris Contandr. & al.
Campanula decumbens A. DC.
Campanula expansa Rudolph
Campanula flaccidula Vatke
Campanula ghilanensis Pall.
Campanula grandis Fisch & C.A. Mey.
Campanula grandis subsp. *rizeensis* (Gèner) Lammers
Campanula haradjanii Rech. f.
Campanula hawkinsiana Hausskn. & Heldr.
Campanula kotschyana A. DC.
Campanula lusitanica Loefl.
Campanula mairei Pau ex Maire
Campanula olympica Boiss.
Campanula *patula* L. subsp. *patula*
Campanula *patula* subsp. *abietina* (Griseb. & Schenk) Simonk.
Campanula *patula* subsp. *alekovyi* Ančev
Campanula *patula* subsp. *costae* (Willk.) Nyman
Campanula *patula* subsp. *epigaea* (Janka) Hayek
Campanula *patula* subsp. *jahorinae* (Malý) Greuter & Burdet
Campanula *persicifolia* L.
Campanula *phrygia* Jaub. & Spach
Campanula *phyctidocalyx* Boiss. & Noë
Campanula *ramosissima* Sm.
***Campanula rapunculus* L. (*)**
Campanula *rapunculus* subsp. *lambertiana* (A. DC.) Rech. f.
Campanula *retrorsa* Labill.
Campanula *sidonensis* Boiss. & Blanche
Campanula *sparsa* Friv.
Campanula *spatulata* Sm. subsp. *spatulata*
Campanula *spatulata* subsp. *filicaulis* (Halácsy) Phitos
Campanula *spatulata* subsp. *spruneriana* (Hampe) Hayek

Section Rotula (Fed.) Victorov (2002)

***Campanula carpatica* Jacq. (*)**

Section Stevenianae (Fed.) Victorov (2002)

***Campanula stevenii* M. Bieb.**
Campanula stevenii subsp. *alberti* (Trauv.) Viktorov
Campanula stevenii subsp. *altaica* (Ledeb.) Fed.
Campanula stevenii M. Bieb. subsp. *stevenii* (*)
Campanula stevenii subsp. *wolgensis* (P.A. Smirn.) Fed.
Campanula stevenii subsp. *beauverdiana* (Fomin) Rech. F. & Schima-Czeika
Campanula stevenii subsp. *turczaninovii* (Fed.) Viktorov

**Subgenus**	**Roucela (Dumort.) Damboldt (1976) = C. sect. Annuae Kharadze (1949)**	
	Campanula creutzburgii Greuter	*Campanula podocarpa* Boiss.
	Campanula delicatula Boiss.	*Campanula raveyi* Boiss.
	Campanula drabifolia Sm.	*Campanula rhodensis* A. DC.
	***Campanula erinus* L. (*)**	*Campanula scutellata* Griseb.
	Campanula kastellorizana Carlström	*Campanula simulans* Carlström
	Campanula pinatzii Greuter & Phitos	*Campanula veneris* Carlström
**Subgenus**	**Scapiflorae (Boiss.) Ogan. (1995) = C. sect. Medium A. DC. subsect. Scapiflorae (Boiss.) Fed. (1957) = Gen. Hemisphaera Kolak. 1984**	
Section	Dasystigma (Fed.) Victorov (2002)	
	***Campanula alpina* Jacq. (*)**	
Section	Scapiflorae (Boiss.) Kharadze (1949)	
	Campanula aldanensis Fed. & Karav.	*Campanula dasyantha* subsp. *chamissonis* (Fed.) Viktorov
	Campanula ardonensis Rupr.	*Campanula kadargavinica* Amirkh. & Komzha
	Campanula bellidifolia Adams	*Campanula kryophila* Rupr.
	Campanula bellidifolia subsp. *argunensis* (Rupr.) Viktorov	*Campanula ledebouriana* Trautv.
	Campanula bellidifolia subsp. *aucheri* (A. DC.) Viktorov	*Campanula petrophila* Rupr.
	Campanula bellidifolia subsp. *besenginica* (Fomin) Viktorov	*Campanula pulvinaris* Hausskn. & Bornm.
	Campanula bellidifolia subsp. *meyerana* (Rupr.) Viktorov	*Campanula radchensis* Kharadze
	Campanula bellidifolia subsp. *saxifraga* (M. Bieb.) Viktorov	*Campanula songutica* Amirkh.
	Campanula bornmuelleri Náb.	*Campanula tridentata* Schreb.
	***Campanula ciliata* Stev. (*)**	*Campanula tridentata* subsp. *biebersteiniana* (Schult.) Ogan.
	Campanula circassica Fomin	*Campanula zeyensis* Amirkh. & Tavasiev
	Campanula dasyantha M. Bieb.	
Aggregat	Rupestres	
	Campanula aghrica Kit Tan & Sorger	*Campanula leucantha* Gilli
	Campanula argentea Lam.	*Campanula leucosiphon* Boiss. & Heldr.
	Campanula ariana Podlech	*Campanula oreadum* Boiss. & Heldr.
	Campanula bipinnatifida P.H. Davis	*Campanula pelia* (Halácsy) Hausskn. & Sint.
	Campanula calaminthifolia Lam.	*Campanula peshmenii* Güner
	Campanula constantini Beauverd & Topali	*Campanula pinnatifida* Hub.-Mor.
	Campanula cymaea Phitos	*Campanula radula* Fisch.
	Campanula demirsoyi Kandemir	***Campanula rupestris* Sm. (*)**
	Campanula dulcis Decne.	*Campanula rupicola* Boiss. & Spruner
	Campanula ekimiana Güner	*Campanula schimaniana* Rech. f.
	Campanula isaurica Contandr. & al.	*Campanula sciathia* Phitos
	Campanula kirikkaleensis Dönmez & Güner	*Campanula scopelia* Phitos
	Campanula koyuncui H. Duman	*Campanula strigillosa* Boiss.
	Campanula lamondiae Rech. f.	*Campanula teucrioides* Boiss.
**Subgenus**	**Theodorovia (Kolak.) Ogan. (1995) = Theodorovia Kolak (1995)**	
	***C. karakuschensis* Grossh. (*)**	

Unclassified	
America	
Campanula angustiflora Eastw.	*Campanula piperi* Howell
***Campanula americana* L. (*)**<br>**= *Campanulastrum americanum* (L.) Small**	*Campanula prenanthoides* Durand
Campanula aparinoides Pursh	*Campanula reverchonii* A. Gray
Campanula aurita Greene	*Campanula robinsiae* Small
Campanula californica (Kellog) Heller	*Campanula scabrella* Engelm.
Campanula divaricata Michx.	*Campanula scouleri* Hooker ex A. DC.
***Campanula exigua* Rattan (*)**	*Campanula sharsmithiae* Morin
Campanula floridana Watson	*Campanula shetleri* Heckard
Campanula griffinii Morin	*Campanula wilkinsiana* Greene
Campanula parryi A. Gray	
Eurasia	
Campanula akhdarensis A.G. Mill. & Whitc.	*Campanula hofmannii* (Pant.) Greuter & Burdet
Campanula alphonsii Wall	*Campanula jordanovi* Ančev & Kovanda
Campanula alsinoides Hook. f.	*Campanula rashtiana* Parsa
Campanula antalyensis Ayasligil & Kit Tan	*Campanula samothracica* (Degen) Greuter & Burdet
Campanula antilibanotica (P.H. Davis) Greuter & Burdet	*Campanula sulaimanii* Nasir
Campanula argyrotricha Wall. ex A. DC.	*Campanula tenuissima* Dunn
Campanula cretica (A. DC.) D. Dietr.	***Campanula uyemurae* (Kudo) Miyabe & Tatew. (*)**<br>**(= *Popoviocodonia uyemurae* (Kudô) Fed.)**
Campanula euxina (Velen.) Ančev	*Campanula wanneri* Rochel
Campanula heterophylla L.	*Campanula wattiana* Nayar & Babu
Campanula hissarica Kamelin	*Campanula yildirimlii* Kit Tan & Sorger
