# Supplementary material for: How to Handle Speciose Clades? Mass Taxon-Sampling as a Strategy towards Illuminating the Natural History of Campanula (Campanuloideae)
Source: PLoS One. 2012 Nov 28;7(11):e50076. doi: 10.1371/journal.pone.0050076 (PMC3509159; doi:10.1371/journal.pone.0050076)
Supplement: Figure S1 — Bayesian majority-rule phylogram of Campanula and relatives (D680). Posterior probability values are indicated below branches. Gray boxes indicate the respective outgroup sister clades; blue boxes refer to “Cam” clades containing at least one accession of Campanula (Cam01 to Cam17; see text). A blue dot indicates the crown node of Campanula s.lat. LOBE = Lobelioideae; CYPHI: Cyphioideae; CA-CYA: Campanuloideae-Cyanantheae; CA-WAH: Campanuloideae-Wahlenbergieae. (PDF) [file pone.0050076.s001.pdf]

Figure S1

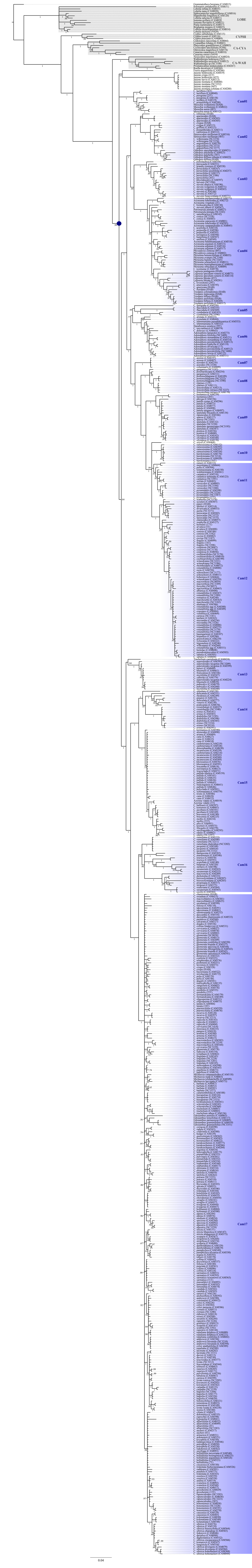

LOBE
CYPH
CA-CYA
CA-WAH
Cam01
Cam02
Cam03
Cam04
Cam05
Cam06
Cam07
Cam08
Cam09
Cam10
Cam11
Cam12
Cam13
Cam14
Cam15
Cam16
Cam17
0.04
